# Type and timing of menopausal hormone therapy and breast cancer risk: individual participant meta-analysis of the worldwide epidemiological evidence

**DOI:** 10.1016/S0140-6736(19)31709-X

**Published:** 2019-09-28

**Authors:** Collaborative Group on Hormonal Factors in Breast Cancer

## Abstract

**Background:**

Published findings on breast cancer risk associated with different types of menopausal hormone therapy (MHT) are inconsistent, with limited information on long-term effects. We bring together the epidemiological evidence, published and unpublished, on these associations, and review the relevant randomised evidence.

**Methods:**

Principal analyses used individual participant data from all eligible prospective studies that had sought information on the type and timing of MHT use; the main analyses are of individuals with complete information on this. Studies were identified by searching many formal and informal sources regularly from Jan 1, 1992, to Jan 1, 2018. Current users were included up to 5 years (mean 1·4 years) after last-reported MHT use. Logistic regression yielded adjusted risk ratios (RRs) comparing particular groups of MHT users versus never users.

**Findings:**

During prospective follow-up, 108 647 postmenopausal women developed breast cancer at mean age 65 years (SD 7); 55 575 (51%) had used MHT. Among women with complete information, mean MHT duration was 10 years (SD 6) in current users and 7 years (SD 6) in past users, and mean age was 50 years (SD 5) at menopause and 50 years (SD 6) at starting MHT. Every MHT type, except vaginal oestrogens, was associated with excess breast cancer risks, which increased steadily with duration of use and were greater for oestrogen-progestagen than oestrogen-only preparations. Among current users, these excess risks were definite even during years 1–4 (oestrogen-progestagen RR 1·60, 95% CI 1·52–1·69; oestrogen-only RR 1·17, 1·10–1·26), and were twice as great during years 5–14 (oestrogen-progestagen RR 2·08, 2·02–2·15; oestrogen-only RR 1·33, 1·28–1·37). The oestrogen-progestagen risks during years 5–14 were greater with daily than with less frequent progestagen use (RR 2·30, 2·21–2·40 *vs* 1·93, 1·84–2·01; heterogeneity p<0·0001). For a given preparation, the RRs during years 5–14 of current use were much greater for oestrogen-receptor-positive tumours than for oestrogen-receptor-negative tumours, were similar for women starting MHT at ages 40–44, 45–49, 50–54, and 55–59 years, and were attenuated by starting after age 60 years or by adiposity (with little risk from oestrogen-only MHT in women who were obese). After ceasing MHT, some excess risk persisted for more than 10 years; its magnitude depended on the duration of previous use, with little excess following less than 1 year of MHT use.

**Interpretation:**

If these associations are largely causal, then for women of average weight in developed countries, 5 years of MHT, starting at age 50 years, would increase breast cancer incidence at ages 50–69 years by about one in every 50 users of oestrogen plus daily progestagen preparations; one in every 70 users of oestrogen plus intermittent progestagen preparations; and one in every 200 users of oestrogen-only preparations. The corresponding excesses from 10 years of MHT would be about twice as great.

**Funding:**

Cancer Research UK and the Medical Research Council.

## Introduction

A previous meta-analysis of the worldwide evidence found that current and recent users of menopausal hormone therapy (MHT) were at an increased risk of breast cancer, but little information was available about the effects of different types of MHT, or about long-term risks after MHT use had ceased.^1^ Since then, much new information has become available, including results from randomised trials (appendix pp 6–15), generally showing greater risks of breast cancer with preparations containing both oestrogen and progestagen than with preparations containing oestrogen alone, but published information on long-term effects of past use has remained limited.

**Figure 1:**
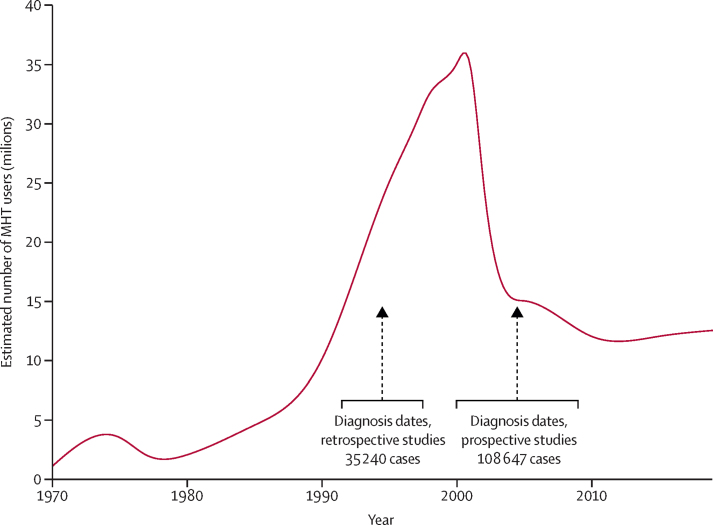
Estimated number of current MHT users in western countries in the 50 years since 1970, and the distribution of the dates of diagnosis of breast cancer in the retrospective and the prospective studies Vertical lines give median dates of diagnosis, and horizontal lines give IQRs. MHT=menopausal hormone therapy.

MHT has been used mostly in western countries, with about 600 million woman-years of use since 1970 (figure 1; appendix p 16, 27).^2^ Use increased rapidly during the 1990s, halved abruptly in the early 2000s, and stabilised during the 2010s with about 12 million current users. Women tend to begin MHT at around the time of the menopause and can continue for several years. At these ages, breast cancer is the most common malignancy in western countries; almost 3% of women are diagnosed with it during their 50s (appendix p 17).^3^ While regulatory bodies in Europe and the USA recommend that MHT be used for the shortest time that it is needed,^4^, ^5^ some clinical guidelines recommended less restrictive prescribing.^6^

**Research in context**

**Evidence before this study**

Reliable assessment of the association between menopausal hormone therapy (MHT) use and breast cancer requires avoidance of appreciable confounding or bias, with important features of MHT use (type used, age at first use, duration of use, and time since last use) classified similarly. Review of published data cannot provide this. We reported in 1997 that current and recent users of MHT were at increased risk of breast cancer, but little information was then available about different types of MHT or about their long-term effects. Systematic searches for published and unpublished studies have been done regularly since 1992 from collaborators and from review articles and reference lists, augmented by periodic searches of MEDLINE and PubMed; search terms included combinations of “breast cancer risk”, “cohort”, “prospective”, “case-control”, “HT”, “hormonal contra*”, “hormone replacement”, “menopaus*”, “reproduc*”, “hormon*”, and “HRT”. The cutoff date for searches was Jan 1, 2018. The randomised evidence, also reviewed here, is largely for hormone use starting after age 60 years.

**Added value of this study**

In an individual participant meta-analysis of all eligible prospective studies, conclusions can now be based on over 100 000 postmenopausal women who developed breast cancer. Half of these women had used MHT, most starting around the time of the menopause at ages 40–59 years. Prospective studies avoid the potential biases in retrospective studies, and the large number of cases limits the play of chance. All types of MHT, except vaginal oestrogens, were associated with increased risks of developing breast cancer. The risks were greater for oestrogen-progestagen than for oestrogen-only preparations, particularly if the progestagen was given daily rather than intermittently. The associations with MHT were much stronger for oestrogen-receptor-positive tumours than for oestrogen-receptor-negative tumours. Among current users, there were definite excess risks even during 1–4 years (mean: 3 years) of MHT use, and progressively greater risks with longer use. During 5–14 years of MHT use, the relative risks (RRs) were similarly increased if MHT use had started at ages 40–44, 45–49, 50–54, and 55–59 years; RRs appeared to be attenuated if MHT use had started after age 60 years. They were also attenuated by adiposity, particularly for oestrogen-only MHT (which had little effect in obese women). After MHT use ceased, some excess risk of breast cancer persisted for more than a decade, which was greater with longer durations of prior MHT use. There was little risk following less than 1 year of use.

**Implications of all the available evidence**

If the associations are largely causal, MHT use in western countries has already caused about 1 million breast cancers, out of a total of about 20 million since 1990. For women of average weight in western countries, 5 years use of oestrogen plus daily progestagen MHT, starting at age 50 years, would increase 20-year breast cancer risks at ages 50–69 years from 6·3% to 8·3%, an absolute increase of 2·0 per 100 women (one in every 50 users). Similarly, 5 years use of oestrogen plus intermittent progestagen MHT would increase the 20-year risk from 6·3% to 7·7%, an absolute increase of 1·4 per 100 women (one in 70 users). Finally, 5 years use of oestrogen-only MHT would increase the 20-year risk from 6·3% to 6·8%, an absolute increase of 0·5 per 100 women (one in 200 users); this excess would be greater in lean women, but in obese women oestrogen-only MHT is associated with little excess risk. For 10 years of use, the 20-year increases in incidence would be about twice as great as for 5 years of use.

Most individual studies were too small to assess reliably the long-term breast cancer risk associated with just a few years of MHT use. Furthermore, some epidemiological evidence remains unpublished, and studies completed before the 2000s necessarily had limited information about long-term effects of past use.^1^ Reliable assessment of the association between breast cancer risk and current and past use of MHT requires review of the totality of the worldwide evidence, with careful control of potential sources of appreciable bias and confounding. Reviews of published data cannot provide this.

The Collaborative Group on Hormonal Factors in Breast Cancer has therefore sought to bring together for central analysis published and unpublished individual participant data from eligible epidemiological studies with information on the type and timing of MHT use. Bringing these studies together has yielded extremely large numbers of cases of breast cancer among women who started MHT in their 40s or 50s.

Although relatively few women in the prospective studies had started MHT in their 60s, most women in the randomised trials of these same preparations were older than 60 years when recruited, as these trials were designed primarily to evaluate possible protective effects against vascular disease (which is less common at younger ages). These and other relevant randomised trials are reviewed separately here.

## Methods

### Study identification and data collection

This collaboration began in 1992.^1^, ^7^, ^8^, ^9^ Since then, potentially eligible epidemiological studies have been sought regularly by computer-aided literature searches, manual searches of review articles, written communications, and discussions at scientific meetings (appendix p 6). Eligible studies, published and unpublished, had sought individual information for postmenopausal women on the type and timing of MHT use and on body-mass index (BMI); and, after 2001, included at least 1000 cases. By Jan 1, 2018, 59 eligible studies had been identified and 58 are included (one retrospective, but no prospective, study was unable to provide data on individual participants; appendix pp 6–15). Randomised trials involved too few cases to be eligible by these criteria, but trials of the main MHT preparations are reviewed separately, as are some trials of anti-oestrogen drugs (appendix pp 28–30).

Prospective studies were included using a nested case-control design, with up to four randomly selected controls per case of invasive breast cancer matched on age, year of birth, and region (appendix p 7). All analyses included only postmenopausal women, defined as known age at natural menopause (or at bilateral oophorectomy) or unknown age at menopause but age of at least 55 years (since 90% with a natural menopause were postmenopausal by 55 years^9^) and excluded younger women with a hysterectomy but unknown age at menopause.

Individual information was sought on sociodemographic, reproductive, and anthropometric factors, and on last reported MHT use prior to the date of cancer diagnosis for cases and the equivalent date for matched controls (hereafter called the index date). Women were classified, using as similar definitions as possible across studies, by age at first MHT use, duration of use, time since last use, and preparation last used. Results for the two common MHT categories, oestrogen-only and oestrogen-progestagen preparations, are examined separately; few women switched between them (appendix p 7).

In prospective studies, information on whether women had been using MHT was necessarily recorded some time before the index date. Some never users or past users might have started after information was last recorded, but this would be uncommon, as the average age at last reported non-use was 63 years, and few women start MHT after age 60 years (appendix p 8). Analyses therefore assumed that never users would not start and past users would not restart (and, for past users, the time since last use was increased by 1 year annually until the index date).

Conversely, women who last (before the index date) reported that they were using MHT might have stopped before the index date. They were included as current users only if the index date was less than 5 years after the last report. Breast cancers that arose within this period did so after mean 1·4 years (SD 1·4). Based on typical annual continuation rates, an estimated 1·1 years of additional use would have accrued among them, far less than the average of 8·9 years (SD 6·5) up to the last reported use. In current users, duration of use was the last-reported duration plus the estimated small additional duration up to the index date (appendix p 8). About 90% of current users at last report would still have been users 1 year before the index date. Sensitivity analyses explored other cutoffs and other assumptions about MHT continuation (appendix pp 20, 42–44).

Breast cancers were classified, where possible, as oestrogen-receptor-positive or oestrogen-receptor-negative (ER+ or ER–); as ductal or lobular; and as localised to the breast or not (appendix p 8).

### Statistical methods

A draft protocol was circulated to collaborators and preliminary results were discussed at a meeting of investigators in 2011 and, after additional data collection and analyses, by correspondence with them in 2017 and 2018. During this collaborative process, the analysis plans evolved.

Conditional logistic regression models comparing particular groups of MHT users with never users yielded odds ratios (equivalent to incidence rate ratios), which are described as relative risks (RRs). To ensure that women in one study were compared directly only with similar women in the same study, all analyses were routinely stratified by study, centre within study, BMI, and fine divisions of age at index date, and adjusted for alcohol consumption, family history of breast cancer, parity, and age at first birth (appendix p 9). To preserve the total number analysed, for each such variable unknowns were assigned to a separate group. Sensitivity analyses restricted analyses to women with complete information for all adjustment variables or examined the effect of adjusting for additional factors. Separate analyses of the prospective and of the retrospective studies were performed.

Age at menopause, an important determinant of breast cancer risk^9^ (and of age at first MHT use), was unknown for about half the cases, but was known for sufficient numbers to estimate with negligible random error any differences between groups in mean age at menopause (appendix p 9). As breast cancer incidence rates in never users increase by a factor of 1·029 per year older at menopause,^9^ any such differences were allowed for not by regression but by increasing or decreasing the RR of breast cancer in each group by a factor of 1·029 for every year of difference in mean age at menopause between that group and the corresponding group of never users.

To help assess the clinical relevance of the RRs, they were combined with the 2015 age-specific breast cancer rates in England, which are typical of many western countries,^3^ to describe the absolute breast-cancer risks at ages 50–69 years that would, in the absence of other causes of death, be associated with starting various types of MHT at age 50 years and continuing for 5 years or for 10 years before stopping. Stata (version 15.0) was used for analyses.

### Role of the funding source

The funders of the study had no role in study design, data analysis, data collection, data interpretation, manuscript preparation, or decision to publish. The writing committee had full access to all the data in the study and had final responsibility for the decision to submit for publication.

## Results

Anonymised information on individual participants was obtained from 58 studies (appendix pp 10, 11), including 143 887 postmenopausal women with invasive breast cancer (cases) and 424 972 without breast cancer (controls). The 24 prospective studies contributed three-quarters (108 647) of the cases, diagnosed in median year 2005 (IQR 2000–09) at mean age 65 years (SD 7); 55 575 (51%) had ever used MHT. The 34 retrospective studies contributed a quarter (35 240) of the cases, diagnosed in median year 1995 (IQR 1992–98); 15 642 (44%) had ever used MHT (figure 1).

Overall, breast cancer risk was greater in MHT ever users than never users (appendix p 31). Since the aim is to assess risks associated with specific types of MHT in relation to the timing of use, further analyses are restricted to 128 435 cases, three-quarters (95 717) in prospective studies, and 366 965 controls with complete information on the type last used, duration of use, and time since last use. With this restriction (appendix p 32) or without it (appendix p 31), the study-specific RRs for ever users versus never users of MHT differed significantly between retrospective and prospective studies, as did the RRs for current users versus never users (appendix p 33). This difference was largely due to lower RRs in North American retrospective studies. The RRs for a particular duration of use of a particular type of MHT were about one fifth lower in the aggregated retrospective than in the aggregated prospective studies (appendix p 19, 34). Given that some MHT users in retrospective studies might have been more willing than non-users to participate as controls, or that there may have been differential recall of use between cases and controls, the main analyses include only the prospective studies, but corresponding analyses for the retrospective studies are in the appendix (pp 50–55). As the prospective studies include three-quarters of the cases, they would have dominated the overall evidence if both study types had been combined.

Among women who developed breast cancer in the prospective studies, mean age at menopause was 50 years (SD 5), mean age at first MHT use was 50 years (SD 6), and mean duration of MHT use was 10 years (SD 6) in current users and 7 years (SD 6) in past users. Hysterectomy largely determined MHT type; the proportions of women reported to have had the procedure were 2710 (7%) of 37 951 for oestrogen-progestagen users, but 31 187 (84%) of 37 213 for oestrogen-only users.

**Figure 2:**
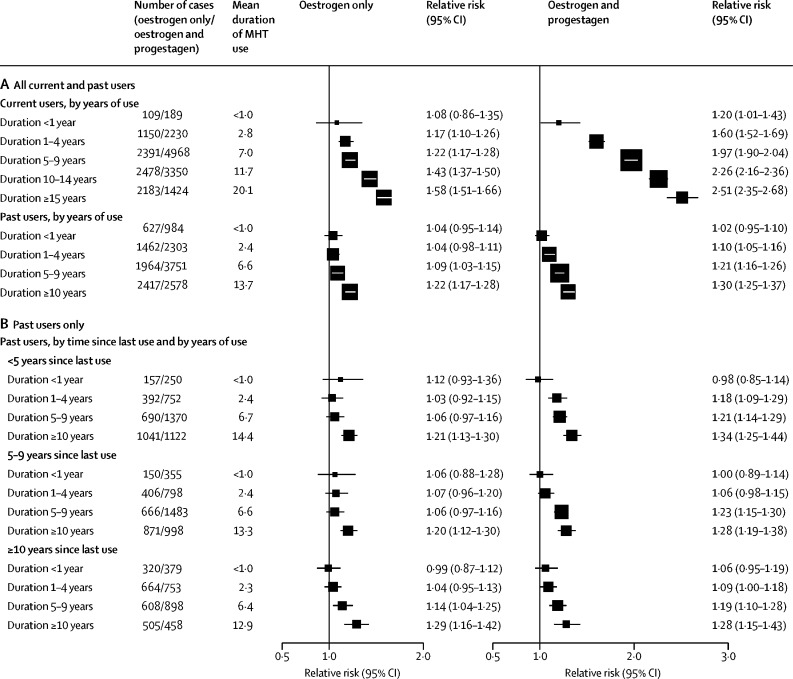
Type and timing of MHT use in current users and past users (A) All current and past users. (B) Past users only, by time since last use of MHT. Fully adjusted relative risks for current versus never users by years of current use, and for past users versus never users by years of use and time since cessation of use (prospective studies). MHT=menopausal hormone therapy.

RRs were consistently greater for oestrogen-progestagen than oestrogen-only preparations, were greater in current than in past users, and (in both current and past users) increased steadily with duration of use (figure 2). For each MHT type, there was a significant excess risk even during years 1–4 (mean: year 3) of current use: the RRs were 1·60 (95% CI 1·52–1·69) for oestrogen-progestagen and 1·17 (1·10–1·26) for oestrogen-only MHT. During years 5–14 (mean: year 9) current use the RRs were 2·08 (2·02–2·15) for oestrogen-progestagen and 1·33 (1·28–1·37) for oestrogen-only MHT. In past users, excess duration-dependent risks persisted for more than a decade after stopping MHT use. There is little information about breast cancer risk associated with past use that had ceased more than 15 years previously.

**Figure 3:**
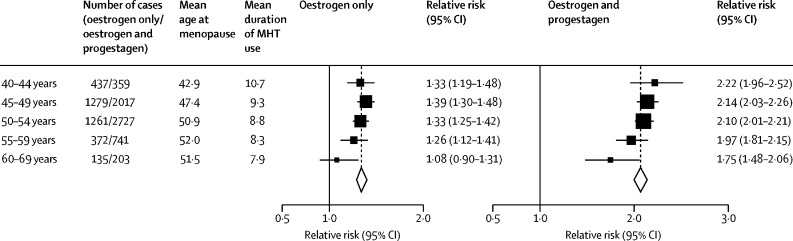
Age at first use: relative risks during years 5–14 of current MHT use Fully adjusted relative risks for current versus never users during years 5–14 of current use, subdivided by age at first use of MHT, giving in each subgroup a mean age at menopause and mean duration of MHT use in cases (prospective studies). Tests for trend in RR with age of start of MHT use (χ^2^, 1 degree of freedom): oestrogen-only MHT 4·04, p=0·044; oestrogen-progestagen MHT 6·44, p=0·011. MHT=menopausal hormone therapy.

Few women had started MHT at ages 30–39 years, but among those who were still current users 15 years later, there were significant risks with oestrogen-progestagen and oestrogen-only preparations (appendix p 40). Substantial numbers of women, however, had started MHT in each of the age groups 40–44, 45–49, 50–54, and 55–59 years, and for each group the RRs were similar (figure 3; appendix pp 35–39). Few women had started MHT at ages 60–69 years, and their excess risks during years 5–14 of current use were significant for oestrogen-progestagen (RR 1·75, 95% CI 1·48–2·06) but not for oestrogen-only MHT (RR 1·08, 0·90–1·31, based on only 135 exposed cases). For comparability, the RRs in figure 3 and in subsequent figures are based on cases arising during years 5–14 of MHT use (which includes most cases in current users).

Sensitivity analyses left the main findings largely unchanged (appendix pp 20, 21, 43, 44). The RRs were little altered by additional adjustment for ethnicity, education, age at menarche, height, and oral contraeptive use, restriction to women with data on all adjustment variables, variation of the 5–year cutoff, exclusion of the largest or the smaller studies, or exclusion of women with an index date of less than 1 year after the last report. Failure to account for any additional use between last-recorded use and cancer diagnosis would, however, have increased duration-specific RRs, especially for less than 1 year MHT use.

**Figure 4:**
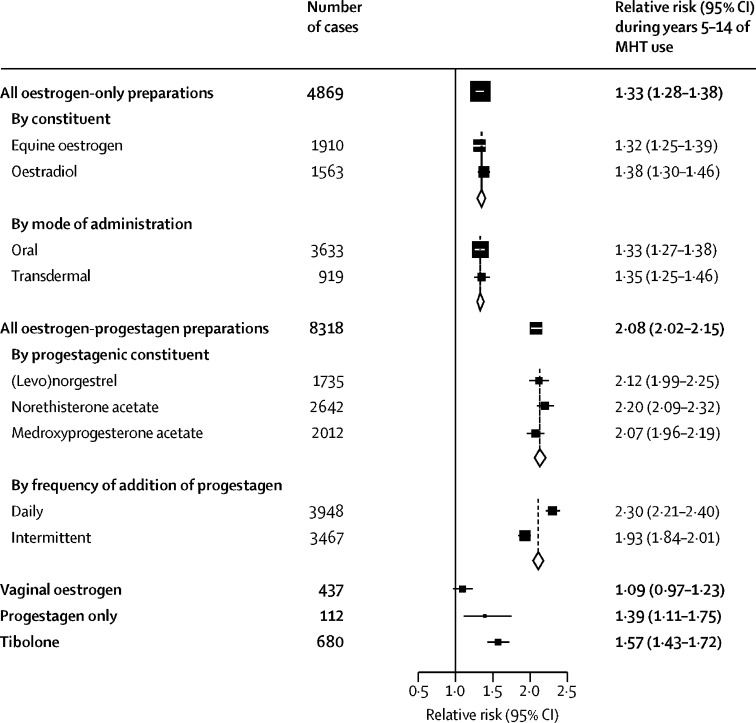
Main types of MHT: relative risks during years 5–14 of current use Fully adjusted relative risks for current versus never users during years 5–14 (mean: year 9) of use. MHT=menopausal hormone therapy. Appendix p 45 gives results for uncommon constituents.

Figure 4 (and appendix pp 22, 23) show breast cancer RRs during years 5–14 (mean: year 9) of current use according to the main constituents, doses, and modes of delivery of the last-used MHT. For oestrogen-only preparations, there was no heterogeneity of risk between equine oestrogen and oestradiol, or between oral administration and transdermal. By contrast, there appeared to be little risk for topical vaginal oestrogens (RR 1·09, 0·97–1·23; p=0·15).

During years 5–14 of use of an oestrogen-progestagen combination, the RR was greater for oestrogen plus daily progestagen than for oestrogen plus intermittent progestagen (which usually involved 10–14 days of progestagen per month); RR 2·30 (2·21–2·40) and RR 1·93 (1·84–2·01), respectively, heterogeneity p<0·0001. In general, the RR did not differ substantially by the progestagenic constituent of the combinations, including rarely used hormones, such as micronised [natural] progesterone (RR 2·05, 1·38–3·56), although the RR appeared to be somewhat lower for oestrogen plus dydrogesterone (appendix p 45). The RR was significantly increased during years 5–14 of progestagen-only MHT (1·39, 1·11–1·75; p=0·0055) and of tibolone (1·57, 1·43–1·72; p<0·0001).

**Figure 5:**
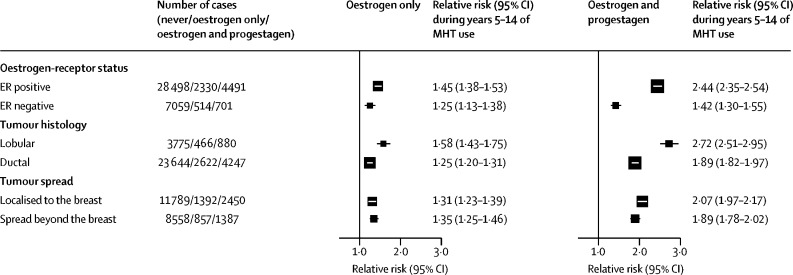
Tumour characteristics: relative risks during years 5–14 of current use Fully adjusted relative risks for current versus never users during years 5–14 (mean: year 9) of use. MHT=menopausal hormone therapy.

Most studies provided data on tumour characteristics. RRs in current users during years 5–14 were substantially greater for ER+ than ER– tumours and for lobular than ductal tumours, but were similar for localised tumours and tumours that had spread beyond the breast (figure 5). The appendix (pp 46–47) gives further results by ER status.

**Figure 6:**
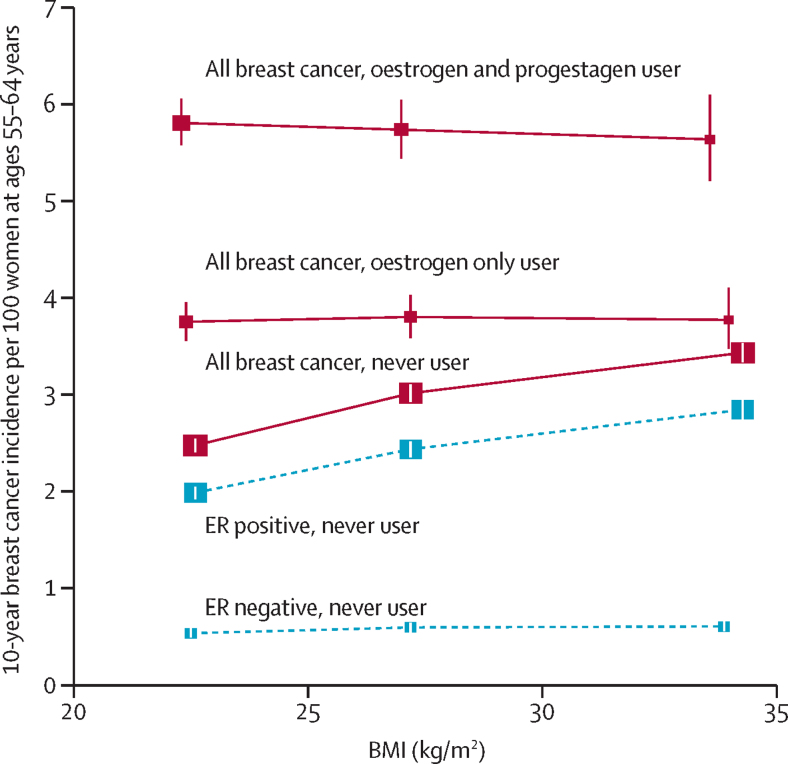
Relevance of BMI to the absolute 10 year breast cancer incidence rate per 100 women at ages 55–64 years in never users and in current users of MHT Adjusted relative risks for all cancers during 5–14 years of current use were calculated taking never users with a BMI of 25–29 kg/m^2^ as the reference, and then standardising to the incidence rate of breast cancer in never users aged 55–64 years of average weight in western countries (ie, 3 per 100 women; appendix p 17). Separate results for ER+ and ER– breast cancer are shown (both with broken lines) only for never users of MHT (but are shown for current users in the appendix, p 48). BMI groups: <25 kg/m^2^ (lean); 25–30 kg/m^2^ (overweight); and ≥30 kg/m^2^ (obese); incidence is plotted against mean BMI values. BMI=body-mass index. ER+=oestrogen-receptor positive. ER–=oestrogen-receptor negative. MHT=menopausal hormone therapy.

The RRs during years 5–14 of MHT use varied little with any personal characteristics except BMI (appendix p 49). Figure 6 shows the absolute breast cancer incidence rates per decade in the age range 55–64 years among never users and among current users during years 5–14 of MHT use. Risk increased with increasing BMI among never users (an association that did not weaken with age, appendix p 26), but not among current users. Hence, the absolute and relative excess risks associated with MHT use were greater for lean women than for obese women. For oestrogen-only MHT, the RRs comparing current users versus never users in the same BMI category were 1·52 (1·45–1·60) for lean, 1·26 (1·19–1·34) for overweight, and 1·10 (1·02–1·20) for obese women. For oestrogen-progestagen MHT, the RRs were 2·36 (2·28–2·45) for lean, 1·92 (1·82–2·02) for overweight, and 1·65 (1·52–1·79) for obese women. Among never users, the incidence of ER– disease was low and little related to BMI, but the incidence of ER+ disease increased with BMI. Hence, ER+ disease accounted for almost all of the association of breast cancer risk with BMI in never users and of the MHT-associated excess risk in users (figure 6; appendix p 48).

To estimate the absolute risks of breast cancer by age 70 years that would be associated with starting various types of MHT at age 50 years and continuing for 5 years or for 10 years, we combined prospective study RRs for women who began any oestrogen-progestagen MHT at ages 45–54 years (appendix p 41) with estimates of the breast cancer incidence rates at ages 50–69 years among never users of average weight in western countries (appendix pp 24–27). The excess risks associated with daily and with intermittent progestagen use were estimated as being, respectively, about one-sixth greater and one-sixth less than this overall excess risk, and the excess risks for oestrogen-only MHT were estimated as being about one-third as great as it (figure 4).

**Figure 7:**
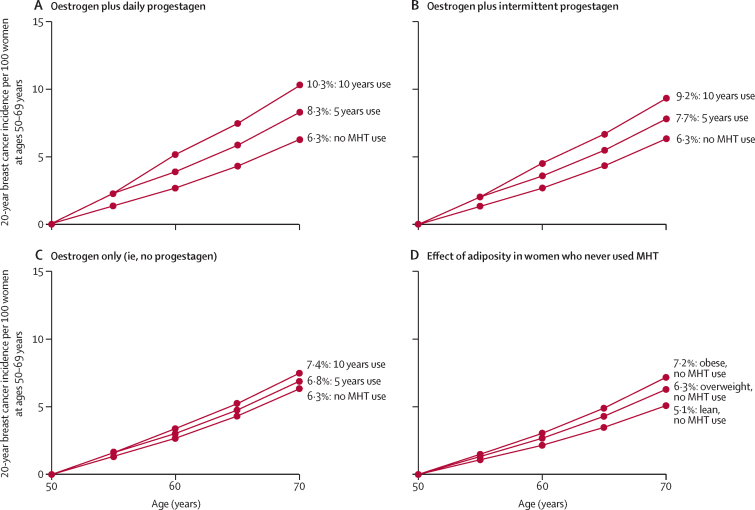
Effect of 5 years or of 10 years of MHT use, starting from age 50 years, on 20-year breast cancer incidence rates Relevance of MHT use and of adiposity to the absolute 20-year risk of developing breast cancer from ages 50–69 years inclusive, assuming that the relative risks found in the prospective studies would apply to women of average weight in a typical developed country in which the absolute 20-year risk of developing breast cancer in never users is 6·3 per 100 women (appendix pp 17, 18, 24, 25, 26). Results for women of average weight are shown for never users and for various categories of MHT use from age 50 years. A) Effects of 5 years or of 10 years of oestrogen-plus-daily-progestagen MHT. B) Effects of 5 years or of 10 years of oestrogen-plus-intermittent-progestagen MHT. C) Effects of 5 years or of 10 years of oestrogen-only MHT. D) Effects of adiposity among women who never use MHT, comparing those of average weight with those who were lean or obese. MHT=menopausal hormone therapy.

These absolute risks of developing breast cancer during the 20-year age range 50–69 inclusive are shown in figure 7. They assume that 6·3% of never users of average weight (ie, 6·3 in every 100 never users of MHT) would develop breast cancer during this 20-year period (appendix pp 17, 18, 24, 25, 26). With 5 years of MHT use followed by 15 years of past use, the 20-year risk for oestrogen-plus-daily-progestagen would then become about 8·3%, an absolute increase of 2·0 per every 100 women (one in every 50 users). For oestrogen-plus-intermittent-progestagen, the risk would become about 7·7%, an absolute increase of 1·4 per 100 women (one in every 70 users). For oestrogen-only the risk would become about 6·8%, an absolute increase of 0·5 per 100 women (one in every 200 users; this excess would be greater in lean women, but slight in obese women). About half the excess would be during the first 5 years of current use of MHT, and half would be during the next 15 years of past use. The 20-year excess risks with 10 years of use from age 50 years would be about double those with 5 years of use.

## Discussion

Users of systemic hormone therapy who started around the time of menopause were at greater risk of invasive breast cancer than apparently similar never users. Excess risks were greater among current than past users, but some risk persisted for more than a decade after MHT use ceased. There was little excess risk after use of MHT for less than 1 year, but there were definite excess risks associated with just 1–4 years of use, and progressively greater risks with longer use. For a given duration of use, the excess risk among current users of MHT was greater for ER+ than for ER– disease. The risk was greater for oestrogen-progestagen than for oestrogen-only preparations, particularly if progestagen use was daily rather than intermittent. Risks did not differ substantially between the main oestrogenic constituents, or by whether oestrogens were administered orally or transdermally. There appeared to be little risk, however, from topical vaginal oestrogen preparations, which limit systemic exposure.^10^ For the oestrogen-progestagen preparations, risks did not generally differ between different progestagenic constituents, including micronised [natural] progesterone, but appeared to be somewhat lower for combinations containing dydrogesterone.^11^

Ovarian function ceases with menopause; thereafter, oestrogen levels fall substantially and progesterone levels fall to near zero. Endogenous oestrogen synthesis in post-menopausal women occurs mainly in adipose tissue, catalysed by the enzyme aromatase. Postmenopausal oestrogen levels therefore correlate strongly with the amount of adipose tissue, and hence with BMI,^12^ and can be reduced to nearly zero by treatment with aromatase inhibitors.^13^

Among postmenopausal women in western countries, ER+ breast cancer accounts for about three-quarters of all breast cancer cases and deaths, and postmenopausal oestrogenic activity is a strong determinant of the incidence of ER+ breast cancer. Among postmenopausal women, the incidence of ER+ breast cancer correlates with age at menopause^9^ and with blood oestrogen levels,^12^ and randomised trials have shown that the incidence of ER+ breast cancer can be greatly reduced by anti-oestrogen drugs such as an aromatase inhibitor, or tamoxifen. In these trials (appendix p 28), allocation to the anti-oestrogen treatment halved the incidence of ER+ breast cancer in postmenopausal women during the scheduled treatment period (RR 0·51, 99% CI 0·39–0·65, Z=–6·9, p<10^−10^). Hence, among postmenopausal women oestrogen-only MHT might be expected to increase ER+ breast cancer incidence rates, as is indicated by the prospective studies.

As postmenopausal adiposity increases endogenous oestrogenic activity and increases ER+ breast cancer incidence, the magnitude of any effect of MHT on breast cancer incidence might be expected to differ by adiposity, and it does. BMI is associated with increased incidence of ER+ disease in postmenopausal never users of MHT (an association that does not weaken with age), but not in women who have been using MHT for some years (figure 6). Obesity therefore attenuates the absolute and the relative excess breast cancer risk associated with MHT. Indeed, for women who are obese, use of oestrogen-only MHT adds little to their already elevated breast cancer risk, suggesting that this addition of an exogenous oestrogen adds relatively little to the adiposity-associated oestrogenic stimulation of their breast tissue. Lean and obese women were, however, similar in the absolute increase in risk produced by addition of a progestagen (ie, in the difference in risk between oestrogen-only MHT and oestrogen plus progestagen MHT).

MHT use is associated with much greater proportional increases in ER+ than ER– disease. Moreover, the excess breast cancer risks in MHT users are strongly duration dependent. Although MHT users differ in various ways from non-users, these biologically plausible findings suggest that the excess of ER+ breast cancer associated with MHT use, which accounts for most of the overall excess of breast cancer associated with MHT use, is largely causal (ie, that some years of MHT use, starting at around the time of the menopause, increases the probability of developing ER+ breast cancer among otherwise similar women of the same age). Although it is unclear why oestrogen-progestagen preparations would have a greater effect than oestrogen-only MHT, there is no good reason to distrust this statistically reliable finding.

There was extensive information in the prospective studies on the effects of starting MHT use at various ages throughout the range 40–59 years. For women who had started anywhere in this age range the RRs among current users were similar, and were all highly significant. Comparatively few women, however, had started such treatment well after the menopause, for example at ages 60–69 years. Among them, the excess risk was statistically definite for oestrogen-progestagen but not for oestrogen-only preparations, although this non-significant result should not be considered in isolation from the highly significant excess risks in the younger age groups.

Further evidence about the effects of starting the use of such treatments well after the menopause is provided by the randomised trials (appendix pp 29, 30), in which the main aim was to assess the effects of such treatments on vascular disease, which is uncommon before age 60 years. Much the largest were the two Women's Health Initiative (WHI) trials, one of oestrogen-only, the other of oestrogen-progestagen hormonal treatment.^14^, ^15^, ^16^ Both prespecified heart disease as the primary outcome, bone fracture as the main secondary outcome, and breast cancer as the main potential adverse outcome; their original power calculations considered the ability to detect a 15–22% increase in breast cancer.^14^

In the oestrogen-only trials the average age at randomisation was 64 years and the mean between-group difference in the duration of hormone treatment during the scheduled intervention phase but before breast cancer onset was about 3·9 years, after allowing for non-compliance (appendix p 29). During or after the intervention phase a total of 434 women developed breast cancer (including 384 in the WHI trial,^17^ of whom 327 [85%] were overweight or obese^18^). The aggregated results suggested an unexpected decrease in risk (RR 0·77, 99% CI 0·60–0·99, Z=–2·7, p=0·01), possibly because starting oestrogen-only therapy well after the menopause does not have the same effect on breast cancer risk as starting at around the time of the menopause.^19^, ^20^ Another possibility, given the epidemiological evidence that oestrogenic stimulation can increase risk and the strong randomised evidence that anti-oestrogen drugs can reduce risk in postmenopausal women, is that the apparently protective effect of hormone treatment in the oestrogen-only trials arose mainly by the play of chance, perhaps augmented by changes in breast density^21^, ^22^, ^23^ somewhat reducing the sensitivity of mammographic screening.

In the oestrogen-progestagen trials (appendix p 30) a total of 864 women developed breast cancer, including 757 in the WHI trial,^17^ and there was no apparent discrepancy with the prospective studies.^14^ Although the between-group difference in the duration of hormone treatment was only about 3·6 years at cancer diagnosis, there was an increase in risk about as great as in the WHI power calculations, albeit with wide 99% confidence limits (RR 1·26, 99% CI 1·06–1·51; *Z*=3·4; p=0·0007).

One limitation of all the available epidemiological evidence is that there is still not long enough follow-up after cessation of prolonged MHT use by women who had started some years of hormonal treatment at around the time of menopause. If excess risks in past users are real and persist much longer than 15 years, there will be some additional hazard after age 70 years. Another limitation is that the collaboration sought information only on breast cancer incidence, not mortality, and incidence can depend on the sensitivity and frequency of mammographic screening. Breast cancer detection rates could have been reduced somewhat by the increase in breast density that is caused by hormonal treatment,^21^, ^22^, ^23^ or increased somewhat in populations where screening frequency is associated with MHT use, as in the US.^24^ The largest prospective study was, however, from the UK, where there is a nationwide mammographic screening programme. In that study, MHT use was not materially associated with screening uptake but was associated with an increase not only in breast cancer incidence (of similar magnitude to that in all prospective studies; appendix p 34) but also in 20-year breast cancer mortality. This increase in breast cancer mortality was greater for oestrogen-progestagen than for oestrogen-only preparations; of the fatal breast cancers, three-quarters with known receptor status were ER+.^25^

Cases continue to accrue in the prospective studies, but there is no good reason to expect further follow-up would materially alter the main findings for current users. To limit biases, the main analyses were restricted to prospective studies, allowed for early menopause decreasing breast cancer risk and bringing forward the age when MHT is started, and adjusted for various other potential confounding factors (as other correlates of breast cancer risk might also affect the type or timing of MHT use). Current users were included up to no more than 5 years after their last reported MHT use; among them there were, on average, only 1·4 years between the last report about their MHT use and their index date. Some additional use must have occurred during that period, as must some discontinuation, but both have been allowed for (although RRs in current users must still have been slightly weakened by discontinuation before the index date). With all these safeguards, the findings are trustworthy for the main patterns of use in the prospective studies.

The clinical relevance of the main findings lies in the magnitude of the absolute risks during and after MHT use for women who start MHT at ages 40–59 years (figure 7; appendix pp 24–27), but the public health relevance depends additionally on the numbers of women previously and currently exposed. Although use of either type of MHT for less than 1 year was associated with little subsequent risk, for women of average weight in developed countries 5 years of use, starting at age 50 years, would cause an appreciable increase in the probability of developing breast cancer at ages 50–69 years. About half the excess would be during the first 5 years of current use of MHT, and half would be during the next 15 years of past use. The absolute increase would be about 2·0 per 100 women (one in every 50 users) for oestrogen-plus-daily-progestagen MHT, 1·4 per 10 women (one in 70 users) for oestrogen-plus-intermittent-progestagen MHT, and 0·5 per 100 women (one in 200 users) for oestrogen-only MHT. There is little difference in the absolute excess incidence by age 70 associated with starting 5 years of MHT use at ages 45 years, 50 years, or 55 years. Thus, addition of a daily progestagen increases the excess risk of breast cancer from one in 200 users to one in 50 users.

The corresponding risks with 10 years of use starting at age 50 years would be about twice as great. In western countries there have been about 20 million breast cancers diagnosed since 1990, of which about 1 million would have been caused by MHT use.

Correspondence to: Secretariat, Cancer Epidemiology Unit, Nuffield Department of Population Health, University of Oxford, Oxford OX37LF, UK **ceu.collaborations@ndph.ox.ac.uk**

## Data sharing

The principal investigators of each contributing study are responsible for access to the data.
