# Supplementary material for: Type and timing of menopausal hormone therapy and breast cancer risk: individual participant meta-analysis of the worldwide epidemiological evidence
Source: Lancet. 2019 Sep 28;394(10204):1159–68. doi: 10.1016/S0140-6736(19)31709-X (PMC6891893; doi:10.1016/S0140-6736(19)31709-X)
Supplement: Supplementary appendix [file mmc1.pdf]

THE LANCET

Supplementary appendix

This appendix formed part of the original submission and has been peer reviewed. We post it as supplied by the authors.

Supplement to: Collaborative Group on Hormonal Factors in Breast Cancer. Type and timing of menopausal hormone therapy and breast cancer risk: individual participant meta-analysis of the worldwide epidemiological evidence. *Lancet* 2019; published online Aug 29. http://dx.doi.org/10.1016/S0140-6736(19)31709-X.

Type and timing of menopausal hormone therapy and breast cancer risk: individual participant meta-analysis of the worldwide evidence

Collaborative Group on Hormonal Factors in Breast Cancer

Contents: CLICK on any page or item to jump to it

Page, Content

Collaborators and Methods

3 List of collaborators
6 Aims, search strategy, and eligibility criteria
7 Data collection and definitions
9 Statistical methods
10 Studies included in this meta-analysis
12 References to epidemiological studies, randomised trials, and methods
16 Trends in MHT use in Western countries
17 Breast cancer risk in never-users of MHT
18 Method of calculating absolute excess risks of breast cancer in MHT users

Supplementary Tables

9	Table S1	Characteristics of MHT users by age at first use
10	Table S2	Summary of participating studies
17	Table S3	Estimated breast cancer risk for women in Western countries in 2015
19	Table S4	Prospective vs retrospective study RRs, current vs never MHT users
20	Tables S5-8	Sensitivity analyses in prospective studies
22	Tables S9	Results in prospective studies by dose of oral oestrogen-only constituents
23	Table S10	Results in prospective studies by oestrogen-progestagen constituents
24	Tables S11-12	Absolute breast cancer risk by age 70 in Western countries with 5 or 10 years of any oestrogen-progestagen MHT, starting at age 45, 50, or 55
26	Table S13	Data for Figure 7
26	Table S14	Effects of obesity on breast cancer incidence in never-users of MHT
27	Table S15	Estimated excess breast cancers resulting from MHT use, 1970-2019
28	Table S16	Meta-analyses of randomised trials of postmenopausal anti-oestrogen
29	Table S17	Meta-analyses of randomised trials of oestrogen-only MHT
30	Table S18	Meta-analyses of randomised trials of oestrogen-progestagen MHT

Supplementary Figures

31	Figs. S1-4	Study-specific RRs comparing users vs never-users of MHT
35	Figs. S5-11	Prospective study RRs by various ages at first use
42	Fig. S12	Prospective study RRs by time from menopause to first use
43	Figs. S13-14	Sensitivity analyses in prospective studies
45	Fig. S15	Prospective study RRs by various hormonal constituents
46	Fig. S16	Prospective study RRs for ER-positive breast cancer
47	Fig. S17	Prospective study RRs for ER-negative breast cancer
48	Fig. S18	Relevance of body mass index and MHT use to ER+ and ER- disease
49	Fig. S19	Prospective study RRs for various subgroups of women
50	Fig. S20	RETROSPECTIVE study RRs, current vs never-users of MHT
51	Fig. S21	RETROSPECTIVE study RRs, by age at first use of MHT
52	Fig. S22	RETROSPECTIVE study RRs, by time from menopause to use
53	Fig. S23	RETROSPECTIVE study RRs, for different MHT preparations
54	Fig. S24	RETROSPECTIVE study RRs, for various tumour characteristics
55	Fig. S25	RETROSPECTIVE study RRs, for various subgroups of women
56	Fig. S26	Menopausal hormone therapy and 20-year breast cancer mortality (Lancet, 29 August 2019)

Writing committee: V Beral, R Peto, K Pirie, G Reeves
Nuffield Department of Population Health, University of Oxford, UK

Collaborators (listed alphabetically by institution or group)

Aichi Cancer Research Institute, Nagoya, Japan: N Hamajima, K Hirose, K Tajima
Albert Einstein College of Medicine, NY, USA: T Rohan
Alberta Health Services, Alberta, Canada: CM Friedenreich
American Cancer Society, GA, USA: SM Gapstur, MM Gaudet
Atlanta, Emory University, GA, USA: RJ Coates, JM Liff
Bank of Cyprus Oncology Center, Nicosia, Cyprus: E Kakouri, Y Marcou
Barts and The London School of Medicine and Dentistry, UK: SW Duffy
Breast Tumor Collaborative Study, Johns Hopkins University, USA: A Morabia, L Schuman, W Stewart, M Szklo
British Columbia Cancer Agency, BC, Canada: P Band, AJ Coldman, RP Gallagher, TG Hislop, P Yang
California Pacific Medical Center, San Francisco, USA: SR Cummings
Canadian Cancer Registries Epidemiology Research Group, Canada: J Hu, KC Johnson, Y Mao
Cancer Center and M. Sklodowska-Curie Institute of Oncology, Warsaw, Poland: P Chao, J Lissowska
Cancer Council NSW, Sydney, Australia: K Canfell
Cancer Prevention Institute of California, Fremont, CA, USA: PL Horn-Ross
Cancer Research Center, University of Hawaii, Hawaii, USA: LM Kolonel, AMY Nomura
Cancer Research UK Genetic Epidemiology Laboratory, Leeds, UK: T Bishop
Cancer Risk Factors and Life-Style Epidemiology Unit, Institute for Cancer Research, Prevention and Clinical Network – ISPRO, Italy: D Palli
Catalán Institut of Oncology, Barcelona, Spain: S De Sanjosé, CA Gonzalez
Centers for Disease Control & Prevention, GA, USA: N Lee, P Marchbanks, HW Ory, HB Peterson, P Wingo
Central Institute of Cancer Research, Berlin, Germany: K Ebeling, D Kunde, P Nishan
Centre for Clinical Research, Uppsala University, Sweden: L Bergkvist
Centre for Genetic Epidemiology, University of Melbourne, Melbourne, Australia: JL Hopper
Centre for Primary Health Care and Equity, University of New South Wales, Sydney, Australia: F Sitas
Centro di Riferimento Oncologico IRCCS, Aviano, Italy: S Franceschi, L Dal Maso
Channing Division of Network Medicine, Brigham and Women's Hospital, Harvard TH Chan School of Public Health, MA, USA: AH Eliassen, RM Tamimi, WC Willett, for Nurses' Health Study I & II Research Group (funded by NCI CA186107 & CA176726)
Chennai Cancer Institute, Madras, India: V Gajalakshmi
Chiang Mai University, Chiang Mai, Thailand: N Martin, T Pardthaisong, S Silpisornkosol, C Theetranont
Chulalongkorn University, Bangkok, Thailand: B Boosiri, S Chutivongse, P Jimakorn, P Virutamasen, C Wongsrichanalai
Columbia University, NY, USA: A Neugut, R Santella
Cyprus Institute of Neurology and Genetics, Nicosia, Cyprus: A Hadjisavvas, K Kyriacou, M Loizidou
Dalla Lana School of Public Health, Toronto, Canada: CJ Baines, N Kreiger, AB Miller, C Wall
Danish Cancer Society Research Center, Copenhagen, Denmark: A Tjonneland
Danish Nurse Cohort Study, Research Centre for Prevention and Health, Capital Region, Denmark: T Jorgensen, C Stahlberg, A Tønnes Pedersen
Department of Biomedical and Clinical Sciences, University of Milan, Italy: E Negri
Department of Biostatistics and Epidemiology, School of Public Health and Health Sciences, University of Massachusetts, Amherst, MA, USA: SE Hankinson
Department of Cancer Epidemiology/ Clinical Cancer Registry, University Cancer Centre Hamburg, Germany: D Flesch-Janys
Department of Clinical Sciences and Community Health, University of Milan, Italy: C La Vecchia
Department of Environmental Medicine, Karolinska Institute, Stockholm, Sweden: N Hakansson
Department of Mathematics, University of Bergen, Norway: I Heuch
Department of Medical Epidemiology and Biostatistics, Karolinska Institutet, Stockholm, Sweden: HO Adami, E Weiderpass
Department of Medicine, Karolinska Institutet, Stockholm, Sweden: A Ekbom, G Erlandsson, I Persson
Department of Public Health Sciences, Karolinska Institute, Stockholm, Sweden: C Magnusson
Department of Public Health, Oxford, UK: L Jones, K McPherson, A Neil, M Vessey, D Yeates
Department of Public Health, Sydney, Australia: G Berry, J Cooper Booth, T Jelihovsky, R MacLennan
Division of Cancer Epidemiology, German Cancer Research Center (DKFZ), Heidelberg, Germany: J Chang-Claude, R Kaaks

Epidemiology and Prevention Division, Center for Public Health Sciences, National Cancer Center, Tokyo, Japan: M Iwasaki, S Tsugane, for the Japan Public Health Center-based Prospective Study Group
Fred Hutchinson Cancer Research Center, WA, USA: GL Anderson, JR Daling, WB Hutchinson, CI Li, KE Malone, M Mandelson, PA Newcomb, EA Noonan, RM Ray, JL Stanford, MTC Tang, DB Thomas, NS Weiss, E White
Girona Cancer Registry, Girona, Spain: A Izquierdo, P Viladiu
H. Lee Moffitt Cancer Center and Research Institute, Tampa, FL, USA: KM Egan
Hospital General de Mexico, Mexico City, Mexico: HR Cuevas, P Ontiveros, A Palet, SB Salazar
Hospital Universitario, Cali, Colombia: N Aristizabal, A Cuadros
Icelandic Cancer Registry, Icelandic Cancer Society, Reykjavik, Iceland: L Tryggvadottir, EJ Olafsdottir
Imperial College, London, UK: E Riboli
INSERM, Institut Gustave-Roussy, Villejuif, France: N Andrieu, A Bachelot, A Brêmond, B Gairard, J Lansac, MG Lê, L Piana, R Renaud
INSERM, Paris South University, Institut Gustave-Roussy, E3N-EPIC Group, Villejuif, France: MC Boutron-Ruault, N Chabbert-Buffet, F Clavel-Chapelon, A Fournier, S Mesrine
Institut Universitaire de Medecine Sociale et Preventive, Lausanne, Switzerland: F Levi
Institute of Cancer Research, London, UK: M Jones, AJ Swerdlow (funded by Breast Cancer Now and ICR)
Institute of Environmental Medicine, Karolinska Institutet, Stockholm, Sweden: A Wolk
Institute of Oncology, Ljubljana, Slovenia: K Kosmelj, M Primic-Zakelj, J Stare
Instituto Nacional de Salud Pública, Morelos, Mexico: G Torres-Mejia
International Agency for Research on Cancer, Lyon, France: I Romieu, E Weiderpass*
International Prevention Research Institute, Lyon, France: P Boyle
Israel Chaim Sheba Medical Centre, Tel-Hashomer, Israel: F Lubin, B Modan, E Ron, Y Wax
Istituto di Statistica Medica e Biometria, Milan, Italy: M Ferraroni
Istituto Nazionale Tumori, Divisione di Statistica Medica e Biometria, Milan, Italy: E Marubini
Julius Centre for Health Sciences and Primary Care, University Medical Center Utrecht, Netherlands: EM Monninkhof, NC Onland-Moret, PHM Peeters
Kaiser Permanente, CA, USA: GD Friedman, RA Hiatt
Léon Bérard Cancer Center, Lyon, France: M Touillaud
Loma Linda University, CA, USA: WL Beeson, G Fraser
London School of Hygiene and Tropical Medicine, UK: J Peto
Long Island Breast Cancer Study, NY, USA: RL Hanson, MC Leske, MC Mahoney, PC Nasca, AO Varma, AL Weinstein
Maastricht University, Maastricht, The Netherlands: RA Goldbohm, PA van den Brandt
Mahidol University, Bangkok, Thailand: N Chantarakul, S Koetsawang, D Rachawat
Memorial Sloan-Kettering Cancer Center, New York, USA: MC Pike
Montpellier Cancer Centre & INSERM, Montpellier, France: M Gerber, S Richardson, C Segala
Mount Sinai School of Medicine, NY, USA: S Teitelbaum
Nairobi Centre for Research in Reproduction, Nairobi, Kenya: D Gatei, P Kenya, A Kungu, JG Mati
National Cancer Institute, MD, USA: LA Brinton, M Freedman, R Hoover, C Schairer, RG Ziegler
National Centre for Epidemiology and Population Health, Canberra, Australia: E Banks
National Institute of Child Health & Human Development, MD, USA: R Spirtas
National University of Singapore, Singapore: HP Lee
Netherlands Cancer Institute, Amsterdam, The Netherlands: MA Rookus, FE van Leeuwen
New Jersey State Department of Health, NJ, USA: JA Schoenberg
Norwegian Institute of Public Health, Oslo, Norway: S Graff-Iversen, R Selmer
Nuffield Department of Population Health, Cancer Epidemiology Unit, Oxford, UK (Secretariat): S Atkinson, I Barnes, V Beral, D Bull, T Gathani, A Goodill, J Green, C Hermon, T Key, K Pirie, G Reeves, K Shaw, TO Yang
Nuffield Department of Population Health, Clinical Trial Service Unit & Epidemiological Studies Unit, Oxford, UK: R Collins, HC Pan, R Peto
Oslo Centre for Biostatistics and Epidemiology, University of Oslo, Oslo, Norway: R Ghiasvand
Radiation Effects Research Foundation, Hiroshima, Japan: K Mabuchi, D Preston
Roswell Park Comprehensive Cancer Center, New York, USA: SE McCann
Royal College of General Practitioners Oral Contraception Study, UK: P Hannaford, C Kay
School of Medicine and Public Health, University of Wisconsin, WI, USA: JM Hampton, LJ Titus, A Trentham-Dietz
School of Public Health and Community Medicine, University of New South Wales, Australia: B Liu
Shanghai Cancer Institute, Shanghai, China: YT Gao, F Jin, J-M Yuan
Shanghai Institute of Planned Parenthood Research, Shanghai, China: HY Wei, T Yun, C Zhiheng
Slone Epidemiology Center, Boston University, USA: PF Coogan, JR Palmer, L Rosenberg
Stanford Cancer Institute and Department of Medicine (Oncology), Stanford University School of Medicine, CA, USA: EM John
Tianjin Cancer Institute, Tianjin, China: Q-S Wang

Tohoku University Graduate School of Medicine, Sendai, Japan: M Kawai, Y Minami, I Tsuji
Tromso University, Tromso, Norway: M Kumle, H Stalsberg
UiT The Arctic University of Norway, Tromso, Norway: E Lund
UKCTOCS, University College London, UK: EO Fourkala, I Jacobs, U Menon, A Ryan
University Hospital, Lund, Sweden: L Hartman, H Olsson
University of Athens Medical School, Athens, Greece: K Katsouyanni, A Trichopoulou, A Tzonou
University of California, San Francisco, CA, USA: KA Baltzell
University of Chile, Santiago, Chile: A Dabancens, L Martinez, R Molina, O Salas
University of Costa Rica, San Jose, Costa Rica: L Rosero-Bixby
University of Edinburgh, Edinburgh, UK: FE Alexander
University of Minnesota School of Public Health, MN, USA: K Anderson, AR Folsom, on behalf of the Iowa Women's Health Study
University of North Carolina at Chapel Hill, Gillings School of Global Public Health, NC, USA: MD Gammon, BS Hulka, R Millikan
University of Nottingham, Nottingham, UK: CED Chilvers
University of Otago, Dunedin, New Zealand: M McCredie, C Paul, DCG Skegg, GFS Spears
University of Padua, School of Medicine, Padova, Italy: F Lumachi
University of Pennsylvania School of Medicine, PA, USA: TR Rebbeck
University of Philippines, Manila, Philippines: RA Apelo, J Baens, JR de la Cruz, B Javier, LB Lacaya, CA Ngelangel
University of Pittsburgh, Pittsburgh, PA, USA: J Cauley, for the Study of Osteoporotic Fractures
University of Queensland, Brisbane, Australia: C Bain, F Schofield, V Siskind
University of Southern California, CA, USA: L Bernstein, S Enger, RW Haile, A Paganini-Hill, G Ursin, AH Wu, MC Yu
University of Southern Denmark, Odense University Hospital, Denmark: M Ewertz
University of Toronto, Canada: EA Clarke
Vanderbilt University, TN, USA: XO Shu, W Zheng
Women's Health Initiative: GL Anderson, M Gass, MJ O'Sullivan, for the WHI investigators**
World Health Organisation, Geneva, Switzerland: A Kalache
World Health Organisation, UNDP/UNFPA/WHO/World Bank Special Programme of Research, Development and Research Training in Human Reproduction, Geneva, Switzerland: TMM Farley, S Holck, O Meirik*
Yamagata University Graduate School of Medicine, Yamagata, Japan: A Fukao
Zhongnan Hospital, Wuhan University, China: X Zhou

***DISCLAIMER:** Where authors are identified as personnel of the International Agency for Research on Cancer / World Health Organization, the authors alone are responsible for the views expressed in this article and they do not necessarily represent the decisions, policy or views of the International Agency for Research on Cancer / World Health Organization.

** The WHI program is funded by the National Heart, Lung, and Blood Institute, National Institutes of Health, and U.S. Department of Health and Human Services through contracts HHSN268201600018C, HHSN268201600001C, HHSN268201600002C, HHSN268201600003C, and HHSN268201600004C.
The authors thank the WHI investigators and staff for their dedication, and the study participants for making the program possible. A full listing of WHI investigators can be found at:
http://www.whi.org/researchers/Documents%20%20Write%20a%20Paper/WHI%20Investigator%20Long%20List. pdf

Aims, search strategy, eligibility criteria, data collection and definitions

This review of epidemiological evidence by the Collaborative Group on Hormonal Factors in Breast Cancer aims to assess the association between use of different types of menopausal hormonal therapy (MHT) and the timing of their use and the risk of incident invasive breast cancer in postmenopausal women. The totality of the worldwide epidemiological evidence, published and unpublished, was sought, and individual participant datasets were obtained and brought together centrally for checking and detailed analysis. A draft protocol for this meta-analysis was circulated to collaborators in 2011, and draft reports were circulated for comment in 2017 and 2018.

The Collaborative Group on Hormonal Factors in Breast Cancer was initiated in 1992 and reported preliminary results on MHT use in 1997 (*Lancet* 1997;**350**:1047–59). From 1 January 1992 until now, potentially eligible epidemiological studies of MHT have continued to be sought regularly by personal contacts, by correspondence with collaborators both at meetings and electronically, by searches of review articles, by searches of references in journal articles, and, after 1997, by frequent searches of MEDLINE and PubMed. Search terms varied, but eventually included a combination of 'breast cancer risk', 'cohort', 'prospective', 'case-control', 'hormonal contracep', 'hormone replacement', 'menopaus*', 'reproduct*' 'hormon*', 'HRT', 'HT' and 'MHT'. In addition, epidemiological studies that had collected relevant data, including those that had not yet published on breast cancer in relation to MHT use, were sought by correspondence with colleagues and by discussions at meetings of the collaborators in 2000, 2005 and 2011. The major studies would all have been identified by more than one of these methods.

To be eligible, epidemiological studies had to have sought individual data for postmenopausal women on the duration and time since last use of MHT, the type of MHT last used, and body mass index. After the year 2001, eligible studies needed to have accrued at least 1000 women with incident invasive breast cancer (not necessarily all in postmenopausal women), whereas earlier studies were eligible with fewer cases.

By 1 January 2018, 59 eligible studies[1-59] had been identified and principal investigators from each had been invited to participate in the collaboration. Datasets from 58 of the 59 eligible studies are included in these analyses.[1-58] The remaining eligible study[59] was retrospective in design, included 3593 cases, and reported a relative risk of 1.2 (95%CI 1.1-1.3) for current or recent use of MHT versus never-use; because of its retrospective design the inclusion or exclusion of this study would not have affected the main findings of the present report, which are based on the studies with information collected prospectively.

Women's Health Initiative randomised trials, and other randomised trials of MHT

When this collaboration began there was little randomised evidence about the effects of menopausal hormone therapy on breast cancer risk. In 1993 two Women's Health Initiative randomized trials began, one of equine oestrogen plus medroxyprogesterone acetate versus placebo and another of equine oestrogen versus placebo. As the principal aim was to assess effects on heart disease, both trials recruited women who were, on average, many years beyond their menopause. Both trials were stopped prematurely, in 2002 and 2004 respectively,[60-61] but follow-up continued during the post-intervention period until 2010.[62] Several smaller randomised trials of an oestrogen with/without a progestagen have also reported on breast cancer incidence.[63-69] The totality of the evidence from the aggregate of all randomised trials is wholly unbiased (except by any effects of treatment on the sensitivity of mammographic screening – see below), so even though each of the meta-analyses of randomised trials involved fewer than 1000 invasive breast cancers their published findings are provided on pp29-30 of this Appendix.

Ineligible studies

Epidemiological studies that had collected information on MHT use and breast cancer risk, but not on the type or timing of MHT used, were ineligible for this meta-analysis. Principal investigators from 31 such studies have contributed to this collaboration but, while their data had been included in the Collaborative Group's 1997 report[70] on breast cancer risk associated with use of any type of MHT, they are not eligible to be included here. Studies with no controls were also ineligible, for example if expected breast cancer cases were calculated from national statistics[71], as were studies that selectively recruited women with screen-detected breast cancer[72] (as MHT reduces the sensitivity and specificity of mammographic screening, so restricting analyses to screen-detected tumours could attenuate the magnitude of any association[73]).

Unpublished data and duplicate data

The Clinical Practice Research Datalink (CPRD, formerly known as GPRD) is a primary care database which includes longitudinal electronic data on drug prescribing and general practitioner (GP) consultations from selected GP practices in the UK.[48,92] It was established in 1987 and covered about 8% of the UK population in 2017. Data on 30,121 women aged 55-74 years with a diagnosis of breast cancer recorded in CPRD in 1995-2016 (and 2 closely matched controls per case) were provided to this collaboration. Most of this CPRD dataset is unpublished, although some of these CPRD cases were included in reports by Opatrny L et al (*BJOG* 2008; **115**: 169-175) and Schneider C et al (*Climacteric* 2009; **12**: 514-524).

Electronic linkage of CPRD and Million Women Study[54] was done in 2014 for 102,000 Million Women Study participants registered at participating CPRD practices in England. To avoid duplication of data, these women (including about 4000 cases) are included only in the CPRD for the present analyses. No information on MHT prescriptions was available in CPRD prior to 1995 or before women were registered in a GP practice that was designated 'up-to-standard'. To allow for any missing data on MHT use, the linked CPRD-Million Women Study data was used to estimate the duration of prior MHT use for all CPRD cases and controls who had an MHT prescription in the first year of available data; this was then added to the available information on MHT use up to the index date. Without allowance for prior MHT use, the total duration of MHT use in CPRD would have been slightly underestimated, so the relative risks of breast cancer for a given duration of MHT use would have been slightly overestimated.

In Norway, 143 women with breast cancer diagnosed in 2006-2007 in the NOWAC (Norwegian Women and Cancer) study[42] had the same year of birth and year of diagnosis as women in the Norwegian Breast Cancer Screening cohort study.[57] For the present analyses, these women were excluded from the Norwegian Breast Cancer Screening cohort data, because of the likelihood of duplication.

The dataset provided by the prospective European EPIC study[50] included some, but not all, of the women who participated in the French E3N study (Fournier et al, *Breast Cancer Res. Treat*. 2008, **107**: 103-11).

Data collection and definitions

For every participating study anonymised information was sought from principal investigators for every case (women with incident invasive breast cancer) and control (women without breast cancer) on socio-demographic, reproductive, anthropometric, and other potential confounding factors. The individual participant datasets contributed by principal investigators were checked and collated centrally so that the present analyses used definitions that were as similar as possible across studies. Apparent inconsistencies were rectified, where possible, by correspondence with the investigators. After the records had been checked and corrected, investigators were sent summary tables and listings of the variables from their study that were to be used in analyses, for final confirmation.

Prospective and retrospective studies

Studies contributing data to this meta-analysis were defined as "prospective" if information on MHT use and other factors was recorded before the diagnosis of breast cancer (and sensitivity analyses explored the effects of excluding from the prospective studies cases with onset less than 1 year after the last report about MHT use; Appendix p21). Prospective studies were included using a nested case-control design: cases were postmenopausal women with incident invasive breast cancer and up to 4 controls were selected for each case, matched by age, year of birth, and broad geographical region, as appropriate. Information on MHT use and other factors relates to that recorded up to the index date, ie the date of diagnosis for cases or the equivalent date for controls. Every prospective study contributed information on the cases and controls, but not on the populations at risk from which the cases and controls were drawn.

Studies were defined as "retrospective" if information on MHT use and other factors was recorded after the diagnosis of breast cancer. In the retrospective studies exposure information was recorded at a time when the cases would have known that they had been diagnosed with breast cancer and the controls would have known that they did not have breast cancer. In some retrospective studies the information on MHT use was not objectively verified (or not objectively verified in comparable ways). Hence, in some retrospective studies there is the potential not only for differential participation but also for differential recording of MHT exposure or of confounding factors between women already diagnosed with breast cancer and unaffected controls.

Defining MHT use and other exposures

Information sought from principal investigators about every woman's use of MHT included: ever-use, current use, age at first use, total duration of use, constituents of each preparation used and duration of use of each preparation. In prospective studies where data on MHT use was updated, information on the last recorded MHT use prior to the index date was sought. Women were excluded from the main analyses if they were recorded as being MHT ever-users, but it was not known whether they were current users or past users.

Systemic MHT was subdivided into oestrogen-only, oestrogen-progestagen, progestagen-only, tibolone, other, or unknown constituents. Most of the present analyses looked separately at women who last used systemic oestrogen-only or oestrogen-progestagen MHT. There is little switching between these two categories of MHT use: in the linked CPRD-Million Women Study data <1% changed each year from one to the other and, if typical, this would have negligible effect on the findings for each category. Oestrogens administered vaginally (as creams

or pessaries, with little systemic exposure) were treated as a group separate from the systemic oestrogen-only preparations.

Users of systemic oestrogen-only preparations were further subdivided by the oestrogenic constituent (equine oestrogen, oestradiol, oestriol, estropipate, or other), by dose, and by whether the oestrogen was given orally or transdermally. Users of oestrogen-progestagen preparations were further subdivided by the progestagenic constituent (medroxyprogesterone acetate, nor-ethisterone, [levo]-norgestrel, dydrogesterone, promegestone, nomegestrol, micronised [natural] progesterone, or other) and by whether the progestagen component was administered daily or intermittently (eg, for 10-14 days per month). No information about use of hormonal intrauterine devices was routinely collected, but in the CPRD dataset <1% of those last prescribed an oestrogen-only MHT had also been prescribed a progestagen-releasing intrauterine device in the 5 years prior to the index date. If this is typical, then few oestrogen-only MHT users would also have been using a progestagen-releasing intrauterine device.

In the prospective studies, current users of MHT were included up to 5 years after MHT use was last recorded. To estimate any additional MHT use during this period we used prescribing data from the linked CPRD-Million Women Study database, which suggested annual MHT continuation rates in current users of MHT to be 90% before 2003 and 70% from 2003 onwards. Based on these assumptions and a cut-off of 5 years after last recorded use for current users, on average about 1.1 (SD 1.1) years of additional duration of MHT use would have accrued during an average of 1.4 (SD 1.4) years between last recorded MHT use and the index date. This is small compared the 8.9 (SD 6.5) years of use that current users had accrued at the time of last recorded use.

Although this average is 1.1 years, our estimate of extra use for each individual depended on her individual index date (which, although on average only 1.4 years after last recorded use, could be up to 5 years after it). About 90% of current users at the time of last recorded use are estimated to have still been current users one year prior to their index date. Sensitivity analyses explored other cut-off times and assumptions about continuation rates (Appendix pp21 and 44).

Some never-users and past users of MHT in the prospective studies may subsequently have started MHT. Both in never-users and in past users, however, the mean age at which they last reported their history of MHT use was 63, ie, well after the menopause, so the present analyses cannot have been materially biased by MHT uptake among never or past users. In confirmation of this for UK women, the annual proportion of never-users of MHT aged 60+ at recruitment into the Million Women Study who became current users was about 1% and 0.1%, respectively, before and after 2003; and among past users the corresponding percentages were about 4% and 0.2%, respectively. Sensitivity analyses explored including never-users and past users only up to 5 years after information on them was last recorded (Appendix p43).

Classification of breast cancers

The present analyses include only invasive breast cancers. Information was sought for every case about tumour characteristics. Most studies reported whether tumours were ductal, lobular, or of other histological types (study references 1, 3, 5, 6, 8, 11, 13-15, 17-18, 20-21, 23-33, 35-43, 46-47, 49-54, 56, 58) and many also provided information on oestrogen receptor status (ER-positive or ER-negative: 12, 15, 19, 23, 25-26, 28-32, 35-37, 41, 43-44, 47-54, 56-58) and/or on whether the tumour had spread past the breast (1, 3, 5, 8, 14-15, 17-25, 28, 30-32, 35-41, 43, 45, 47, 49-54, 56, 58); where tumour stage rather than spread was provided, Stage 1 tumours were classified as localised to the breast, and Stage 3 tumours were included with the Stage 2 or Stage 4 tumours that had spread past the breast.

Statistical Methods

Reliable assessment of any association of MHT use with breast cancer by the type of MHT used, as well as by the duration and time since last use, requires large numbers and reliable allowance for potential sources of appreciable bias. To evaluate these associations with little bias, findings in the aggregated prospective and retrospective studies were presented separately and compared but, if different, the main analyses emphasised evidence from only the prospective studies. The types of MHT used differed by region, eg., the proportion of oestrogen-progestagen users was greater in Europe than in North America, whereas the proportion of oestrogen-only users was greater in North America. Hence, some results are presented separately for studies in Europe and in North America.

To ensure women in one study were compared directly only with similar women in the same study, analyses were routinely stratified by study, centre or region within study, age (<40, single years of age from 40 to 69, 70-74, 75-79 and 80-89), body mass index (BMI <25, 25-29, 30+ kg/m^2; 'lean' = <25 and 'obese' = 30+ kg/m^2); adjusted for family history (first degree relative with breast cancer, yes or no), alcohol consumption (0, <10, 10+ g/week), and reproductive history (nulliparous, and, among parous women, by parity [1-2, 3+] and age at first birth [<20, 20-29, 30+ years]). They also allowed for age at menopause, but in a different way (see below), as this important information was not always available. For all stratification or adjustment factors the data provided by the principal investigators were used, with definitions as similar as possible across studies.

Most women begin taking MHT at around the time of their menopause. Among current users in the prospective studies, the earlier their menopause, the younger they were when they started MHT, and the longer their duration of use (Table S1). Age at menopause was unknown for about half the exposed cases, but this sufficed to estimate with negligible random error any differences between categories of MHT use in their mean age at menopause. As breast cancer risks in never-users are known to increase by a factor of 1.029 per year older at menopause,[74] any such differences were allowed for not by regression but by increasing or decreasing the RR of breast cancer in each exposure group by a factor of 1.029 for every year of difference in mean age at menopause between that group and the corresponding group of never-users.

Table S1: MHT use by age at first use

Age at first MHT use	Mean age at menopause	Mean duration of use (years)
30-39	37	19
40-44	43	16
45-49	47	11
50-54	51	8
55-59	52	7
60-69	51	5

As the reference group of never-users is large, allowance for statistical variation in it makes no material difference to the confidence intervals for any of the relative risks (RRs). Hence, the terms "confidence interval (CI)" and "group-specific (g-s) CI" can be used interchangeably, and although group-specific CIs were calculated for some analyses they are referred to simply as CIs.

Statistical detail: When several groups are compared, one being the large reference group with RR=1, the variance of the log risk in each, including the reference group, can be calculated from the variances and covariances of the log RR values in all other groups (Plummer M. Improved estimates of floating absolute risk. *Stat Med* 2004; **23**: 93-104). For each group the variance of the log risk yields the group-specific confidence limits for log RR, exponentiation of which yields the group-specific CI for that RR. Unless otherwise indicated, 95% CIs are used in the text, tables and figures, but where 99% CIs are given this is indicated.

Comparisons across different subgroups were made using chi-squared tests for heterogeneity of the effect size.

Prospective and retrospective epidemiological studies in the present meta-analyses

The 58 studies included in this meta-analysis are listed in Table S2, by study design (prospective or retrospective) and, within each design, by region (Europe or North America). Within each design and region studies are ordered by the median year when the breast cancers were diagnosed. Data from 20 (5 prospective and 15 retrospective) of the 58 studies marked with an asterisk (*) in the table below had been included in the 1997 publication by this collaboration.[70] Current users in prospective studies were included only up to 5 years after MHT use was last recorded. The 58 studies included a total of 143,887 postmenopausal women with incident invasive breast cancer. Overall about half the cases came from Europe and half from North America. The prospective studies included about three-quarters (108,647) of the cases.

Further details the about individual studies can be found in reference numbers 1-58 (listed on pages 12-14 in this appendix; and in Breast cancer and hormonal contraceptives: further results. *Contraception* 1996; **54**: 1S-106S).

Table S2: Summary of participating studies (Superscripts correspond to the reference list on pp 12-14)

	Number of cases	Number of controls	Mean age (years) at diagnosis of cases	Median (IQR) year of diagnosis of cases
**All studies**	**143,887**	**424,972**	**64.3**	**2002 (1997,2008)**
**All prospective studies**	**108,647**	**382,347**	**65.0**	**2005 (2000,2009)**
**All retrospective studies**	**35,240**	**42,625**	**62.3**	**1995 (1992,1998)**
**12 prospective studies in Europe**				
*Persson [9]	156	468	60.1	1981 (1979,1982)
*Oxford FPA [10]	11	57	50.8	1985 (1983,1986)
Danish Nurses [36]	156	616	63.2	1995 (1994,1997)
EPIC [50]	3,286	13,821	63.9	1999 (1997,2002)
NOWAC [42]	613	2,389	62.5	2001 (1999,2004)
Southern Sweden [33]	418	1,751	66.0	2001 (1996,2004)
Icelandic cohort [58]	542	2196	67.2	2002 (1997,2005)
Norway/Sweden Women's Health [35]	212	871	57.7	2005 (2004,2007)
Million Women Study [54]	43,022	169,041	63.9	2006 (2002,2009)
CPRD [48†]	30,121	60,242	66.0	2008 (2004,2012)
Generations Study [56]	701	2,860	63.6	2009 (2008,2011)
Norwegian Breast Screening Study [57]	3,496	16,048	61.9	2011 (2009,2013)
**All prospective studies, Europe**	**82,734**	**270,360**	**64.6**	**2007 (2002,2010)**
**12 prospective studies in North America**				
*Kaiser Permenante [4]	99	93	50.5	1974 (1972,1976)
Baltzell [7]	48	182	67.1	1985 (1979,1992)
*BCDDP Follow-up Study [26]	2,347	5,994	65.1	1990 (1986,1994)
SOF [19]	373	1,516	76.4	1994 (1991,1998)
*Nurses' Health Study I [15]	7,116	28,835	64.5	1997 (1990,2001)
CPS-II Nutrition [28]	2,530	12,741	68.1	1998 (1995,2000)
Multiethnic Cohort [40]	2,404	18,014	69.1	1998 (1996,2002)
NIH-AARP [46]	3,156	13,392	65.8	1999 (1997,2001)
WHI-Observational Study [49]	2,906	11,612	67.8	2000 (1998,2002)
California Teachers [53]	3,557	13,368	67.0	2002 (1998,2007)
PLCO [51]	907	4,027	70.4	2003 (2000,2006)
Nurses' Health Study II [52]	470	2,213	52.6	2004 (2001,2006)
**All prospective studies, North America**	**25,913**	**111,987**	**66.3**	**1999 (1995,2002)**

Table S2 (continued)

	Number of cases	Number of controls	Mean age (years) at diagnosis of cases	Median (IQR) year of diagnosis of cases
**10 retrospective studies in Europe (including one study in Australia)**				
*Vessey [3‡]	449	518	54.8	1981 (1981,1982)
*Ewertz [38]	752	692	60.6	1983 (1983,1983)
*CRC/ICRF [39]	108	129	51.0	1991 (1990,1991)
*La Vecchia [16‡]	1,520	1,653	61.5	1992 (1992,1993)
*Levi [20‡]	152	328	62.5	1993 (1992,1994)
GESBC [24]	82	118	46.2	1994 (1993,1994)
Magnusson [23]	2,613	2,964	63.3	1994 (1993,1994)
Polish Breast Cancer Study [41]	1,264	1,580	61.8	2001 (2000,2002)
MARIE [47]	3,173	6,510	62.9	2003 (2002,2004)
CLEAR [55††]	817	607	64.1	2009 (2007,2010)
**All retrospective studies, Europe**	**10,930**	**15,099**	**62.0**	**1994 (1993,2003)**
**24 retrospective studies in North America**				
*Leisure World [1]	106	224	67.6	1975 (1973,1976)
*BCDDP Case-Control Study [2]	1,293	1,623	60.5	1977 (1975,1978)
*Nomura [6]	198	219	62.4	1978 (1976,1979)
*CASH [8]	602	787	50.3	1981 (1981,1982)
*Clarke [12]	308	621	59.7	1984 (1983,1985)
*Long Island Study [14]	732	758	62.5	1984 (1984,1985)
Asian American I [21]	106	251	51.6	1985 (1984,1986)
Baltzell [34]	405	788	60.9	1985 (1983,1998)
Western New York [11]	429	482	62.9	1987 (1987,1988)
*Daling [5]	20	31	39.3	1988 (1987,1989)
Ross/Pike [25]	1,711	1,636	61.3	1989 (1988,1992)
*Stanford/Habel [18]	364	394	58.6	1989 (1988,1989)
*Yang/Gallagher [13]	597	603	63.2	1989 (1988,1989)
*WISH [22]	61	62	50.1	1991 (1991,1992)
4-State Study [17]	4,758	4,832	66.8	1992 (1992,1993)
CARE [30]	2,096	2,205	56.8	1996 (1995,1997)
Friedenreich [37]	645	646	64.7	1996 (1995,1996)
Kreiger [29]	1,833	1,732	62.6	1997 (1996,1997)
LIBCSP [44]	814	918	65.4	1997 (1996,1997)
Newcomb [27]	3,572	4,303	60.3	1997 (1996,1998)
San Francisco Bay Area [31]	1,331	1,595	63.1	1997 (1996,1999)
Asian American II [45]	523	480	61.3	1998 (1996,2000)
PACE [32]	970	1,005	71.9	1998 (1997,1998)
WISE [43]	836	1,331	64.5	2000 (2000,2001)
**All retrospective studies, North America**	**24,310**	**27,526**	**62.5**	**1995 (1990,1997)**

* Indicates studies also included in the 1997 publication by this collaboration[70]
‡ Indicates retrospective studies with hospital controls; all other retrospective studies used population controls
† Primary care database[92]
†† This study was done in Australia

References to epidemiological studies, randomised trials, and methods

1. Ross RK, Paganini-Hill A, Gerkins VR, et al. A case-control study of menopausal estrogen therapy and breast cancer. *JAMA* 1980;**243**:1635-1639.
2. Brinton LA, Hoover RM, Szklo M, Fraumeni JF. Menopausal estrogen use and risk of breast cancer. *Cancer* 1981;**47**:2517-22.
3. Vessey M, Baron J, Doll R, McPherson K, Yeates D. Oral contraceptives and breast cancer: final report of an epidemiological study. *Br J Cancer* 1983;**47**:455-462.
4. Hiatt RA, Bawol R, Friedman GD, Hoover R. Exogenous estrogen and breast cancer after bilateral oophorectomy. *Cancer* 1984;**54**:139-144.
5. McDonald JA, Weiss NS, Daling JR, Francis AM, Polissar L. Menopausal estrogen use and the risk of breast cancer. *Breast Cancer Res Treat* 1986;**7**:193-99.
6. Nomura AMY, Kolonel LN, Hirohata T, Lee J. The association of replacement estrogens with breast cancer. *Int J Cancer* 1986;**37**:49-53.
7. Ernster VL, Wrensch MR, Petrakis NL, et al. Benign and malignant breast disease: initial study results of serum and breast fluid analyses of endogenous estrogens. *JNCI* 1987;**79**:949-60.
8. Wingo PA, Layde PM, Lee NC, Rubin G, Ory HW. The risk of breast cancer in postmenopausal women who have used estrogen replacement therapy. *JAMA* 1987;**257**:209-15.
9. Bergkvist L, Adami H-O, Persson I, Hoover R, Schairer C. The role of breast cancer after estrogen and estrogen-progestin replacement. *N Engl J Med* 1989;**321**:293-297.
10. Vessey MP, McPherson K, Villard-Mackintosh L, Yeates D. Oral contraceptives and breast cancer: latest findings in a large cohort study. *Br J Cancer* 1989;**59**:613-17.
11. Graham S, Hellmann R, Marshall J, et al. Nutritional epidemiology of postmenopausal breast cancer in western New York. *Am J Epidemiol* 1991;**134**:552-66.
12. Palmer JR, Rosenberg L, Clarke EA, Miller DR, Shapiro S. Breast cancer risk after estrogen replacement therapy: results from the Toronto Breast Cancer Study. *Am J Epidemiol* 1991;**134**:1386-95.
13. Yang CP, Daling JR, Band PR, Gallagher RP, White E, Weiss NS. Noncontraceptive hormone use and risk of breast cancer. *Cancer Causes Control* 1992;**3**:475-79.
14. Weinstein AL, Mahoney MC, Nasca PC, Hanson RL, Leske MC, Varma AO. Oestrogen replacement therapy and breast cancer risk: a case-control study. *Int J Epidemiol* 1993;**22**:781-89.
15. Colditz GA, Hankinson SE, Hunter DJ, et al. The use of estrogens and progestins and the risk of breast cancer in postmenopausal women. *N Engl J Med* 1995;**332**:1589-93.
16. La Vecchia C, Negri E, Franceschi S et al. Hormone replacement treatments and breast cancer risk: a co-operative Italian study. *Br J Cancer* 1995;**72**:244-8
17. Newcomb PA, Longnecker MP, Storer BE, et al. Long-term hormone replacement therapy and risk of breast cancer in postmenopausal women. *Am J Epidemiol* 1995;**142**:788-95.
18. Stanford JL, Weiss NS, Voigt LF, Daling JR, Habel LA, Rossing MA. Combined estrogen and progestin hormone replacement therapy in relation to risk of breast cancer in middle-aged women. *JAMA* 1995;**274**:137-42.
19. Cauley JA, Lucas FL, Kuller LH, Vogt MT, Browner WS, Cummings SR. Bone mineral density and risk of breast cancer in older women: the study of osteoporotic fractures. Study of Osteoporotic Fractures Research Group. *JAMA* 1996;**276**:1404-8.
20. Levi F, Lucchini F, Pasche C, La Vecchia C. Oral contraceptives, menopausal hormone replacement treatment and breast cancer risk. *Eur J Cancer Prev* 1996;**5**:259-266.
21. Wu AH, Ziegler RG, Pike MC, et al. Menstrual and reproductive factors and risk of breast cancer in Asian-Americans. *Br J Cancer* 1996;**73**:680-6.
22. Brinton LA, Gammon MD, Malone KE, Schoenberg JB, Daling JR, Coates RJ. Modification of oral contraceptive relationships on breast cancer risk by selected factors among younger women. *Contraception* 1997;**55**:197-203.
23. Magnusson C, Baron JA, Correia N, Bergstrom R, Adami H-O, Persson I. Breast cancer risk following long-term oestrogen- and oestrogen-progestin-replacement therapy. *Int J Cancer* 1999;**81**:339-344.
24. Chang-Claude J, Eby N, Kiechle M, Bastert G, Becher H. Breastfeeding and breast cancer risk by age 50 among women in Germany. *Cancer Causes Control* 2000;**11:**687-695.
25. Ross RK, Paganini-Hill A, Wan PC, Pike MC. Effect of hormone replacement therapy on breast cancer risk: estrogen versus estrogen plus progestin. *J Natl Cancer Inst* 2000;**92**:328-332.
26. Schairer C, Lubin J, Troisi R, Sturgeon S, Brinton L, Hoover R. Menopausal estrogen and estrogen-progestin replacement therapy and breast cancer risk. *JAMA* 2000;**283**:485-491.
27. Trentham-Dietz A, Newcomb PA, Egan KM, et al. Weight change and risk of postmenopausal breast cancer (United States). *Cancer Causes Control* 2000;**11**:533-42.
28. Calle EE, Rodriguez C, Jacobs EJ, et al. The American Cancer Society Prevention Study II Nutrition Cohort: rationale, study design, and baseline characteristics. *Cancer* 2002;**94**:2490-2501.
29. Kirsh V, Kreiger N. Estrogen and estrogen-progestin replacement therapy and risk of postmenopausal breast cancer in Canada. *Cancer Causes Control* 2002;**13**:583-590.

30. Weiss LK, Burkman RT, Cushing-Haugen KL, et al. Hormone replacement therapy regimens and breast cancer risk. *Obstet Gynecol* 2002;**100**:1148-1158.
31. John EM, Horn-Ross PL, Koo J. Lifetime physical activity and breast cancer risk in a multiethnic population: the San Francisco Bay Area breast cancer study. *Cancer Epidemiol Biomarkers Prev* 2003;**12**:1143-1152.
32. Li CI, Malone KE, Porter PL, et al. Relationship between long durations and different regimens of hormone therapy and risk of breast cancer. *JAMA* 2003;**289**:3254-3263.
33. Olsson HL, Inqvar C, Bladstrom A. Hormone replacement therapy containing progestins and given continuously increases breast carcinoma risk in Sweden. *Cancer* 2003;**97**:1387-1392.
34. Wrensch M, Chew T, Farren G, et al. Risk factors for breast cancer in a population with high incidence rates. *Breast Cancer Res* 2003;**5**:R88-102.
35. Braaten T, Weiderpass E, Kumle M, Adami H-O, Lund E. Education and risk of breast cancer in the Norwegian-Swedish Women's lifestyle and health cohort study. *Int J Cancer* 2004;**110**:579-83.
36. Stahlberg C, Pedersen AT, Lynge E, et al. Increased risk of breast cancer following different regimens of hormone replacement therapy frequently used in Europe. *Int J Cancer* 2004;**109**:721-7.
37. Csizmadi I, Friedenreich CM, Bryant HE, Courneya KS. An analysis of the effect of selection bias on the association of hormone replacement therapy and breast cancer risk. *Chronic Dis Can* 2005;**26**:73-79.
38. Ewertz M, Mellemkjaer L, Poulsen AH, et al. Hormone use for menopausal symptoms and risk of breast cancer. A Danish cohort study. *Br J Cancer* 2005;**92**:1293-1297.
39. Magnusson CM, Roddam AW, Pike MC, et al. Body fatness and physical activity at young ages and the risk of breast cancer in premenopausal women. *Br J Cancer* 2005;**93**:817-24.
40. Lee S, Kolonel L, Wilkens L, Wan P, Henderson B, Pike M. Postmenopausal hormone therapy and breast cancer risk: the Multiethnic Cohort. *Int J Cancer* 2006;**118**:1285-91.
41. Park SK, Garcia-Closas M, Lissowska J, et al. Intrauterine environment and breast cancer risk in a population-based case-control study in Poland. *Int J Cancer* 2006;**119**:2136-41.
42. Lund E, Bakken K, Dumeaux V, et al. Hormone replacement therapy and breast cancer in former users of oral contraceptives - The Norwegian women and cancer study. *Int J Cancer* 2007;**121**:645-648.
43. Rebbeck TR, Troxel AB, Norman S, et al. Pharmacogenetic modulation of combined hormone replacement therapy by progesterone-metabolism genotypes in postmenopausal breast cancer risk. *Am J Epidemiol* 2007;**166**:1392-9.
44. Shantakumar S, Terry MB, Paykin A, et al. Age and menopausal effects of hormonal birth control and hormone replacement therapy in relation to breast cancer risk. *Am J Epidemiol* 2007;**165**:1187-1198.
45. Wu AH, Yu MC, Tseng CC, Pike MC. Body size, hormone therapy and risk of breast cancer in Asian-American women. *Int J Cancer* 2007;**120**:844-52.
46. Brinton LA, Richesson D, Leitzmann MF, et al. Menopausal Hormone Therapy and Breast Cancer Risk in the NIH-AARP Diet and Health Study Cohort. *Cancer Epidemiol Biomarkers Prev* 2008; **17**:3150-3160.
47. Flesch-Janys D, Slanger T, Mutschelknauss E, et al. Risk of different histological types of postmenopausal breast cancer by type and regimen of menopausal hormone therapy. *Int J Cancer* 2008;**123**:933-41.
48. Herrett E, Gallagher AM, Bhaskaran K, et al. Data Resource Profile: Clinical Practice Research Datalink (CPRD). *Int J Epidemiol* 2015:827–836 (CPRD data were also included in Opatrny L, Dell'Aniello S, Assouline S, Suissa S. Hormone replacement therapy use and variations in the risk of breast cancer. *BJOG* 2008;**115**:169-175; and Schneider C et al. Risk of gynecological cancers in users of estradiol/dydrogesterone or other HRT preparations. *Climacteric* 2009;**12**:514-524)
49. Prentice R, Chlebowski RT, Stefanick ML, et al. Estrogen Plus Progestin Therapy and Breast Cancer in Recently Postmenopausal Women. *Am J Epidemiol* 2008;**167**:1207-1216
50. Bakken K, Fournier A, Lund E, et al. Menopausal hormone therapy and breast cancer risk: impact of different treatments. The European Prospective Investigation into Cancer and Nutrition. *Int J Cancer* 2011;**128**:144-156.
51. Lacey JV Jr, Kreimer AR, Buys SS, et al. Breast cancer epidemiology according to recognized breast cancer risk factors in the Prostate, Lung, Colorectal and Ovarian (PLCO) Cancer Screening Trial Cohort. *BMC Cancer* 2009;**9**:84.
52. Hunter DJ, Colditz GA, Hankinson SE, et al. Oral contraceptive use and breast cancer: a prospective study of young women. *Cancer Epidemiol Biomarkers Prev* 2010;**10**:2496-502.
53. Saxena T, Lee E, Henderson KD, et al. Menopausal hormone therapy and subsequent risk of specific invasive breast cancer subtypes in the California Teachers Study. *Cancer Epidemiol Biomarkers Prev* 2010;**19**:2366-2378.
54. Beral V, Reeves G, Bull D, Green J, for the Million Women Study Collaborators. Breast cancer risk in relation to the interval between menopause and starting hormone therapy. *J Natl Cancer Inst* 2011;**103**:296–305

55. Salagame U, Banks E, Sitas F, Canfell K. Menopausal hormone therapy use and breast cancer risk in Australia: Findings from the New South Wales Cancer, Lifestyle and Evaluation of Risk study. *Int J Cancer* 2016;**138**:1905–1914.
56. Jones ME, Schoemaker MJ, Wright L, et al. Menopausal hormone therapy and breast cancer: what is the true size of the increased risk? *Br J Cancer* 2016;**115**:607-15.
57. Román M, Graff-Iversen S, Weiderpass E, et al. Post-menopausal hormone therapy and breast cancer prognostic characteristics. A linkage between nation-wide registries. *Cancer Epidemiol Biomarkers Prev* 2016;**25**:1464-1473
58. Kjartansdottir OJ, Sigurdardottir LG, Olafsdottir EJ, Jonasson JG, Ursin G, Tryggvadottir L. Estrogen-progestin use and breast cancer characteristics in lean and overweight postmenopausal women. *Breast Cancer Res Treat* 2017;**163**:363-373
59. Dinger JC, Heinemann LA, Möhner S, Thai do M, Assmann A. Breast cancer risk associated with different HRT formulations: a register-based case-control study. *BMC Women's Health* 2006;**6**:13.
60. Writing Group for the Women's Health Initiative Investigators. Risks and benefits of estrogen plus progestin in healthy postmenopausal women: principal results from the Women's Health Initiative randomized controlled trial. *JAMA* 2002;**288**:321-33.
61. Anderson GL, Limacher M, Assaf AR, et al.; Women's Health Initiative Steering Committee. Effects of conjugated equine estrogen in postmenopausal women with hysterectomy: the Women's Health Initiative randomized controlled trial. *JAMA* 2004;**291**:1701–1712.
62. Manson JA, Chlebowski RT, Stefanick ML et al. Menopausal hormone therapy and health outcomes during the intervention and extended poststopping phases of the Women's Health Initiative randomised trials. *JAMA* 2013;**310**:1353-68.
63. The Writing Group for the PEPI Trial. Effects of estrogen or estrogen/progestin regimens on heart disease risk factors in postmenopausal women. *JAMA* 1995;**273**:199-208.
64. Hully S et al. Randomised trial of estrogen plus progestin for secondary prevention of coronary heart disease in postmenopausal women. *JAMA* 1998:**280**:605-613.
65. Herrington DM et al. Effects of estrogen replacement on the progression of coronary-artery atherosclerosis. *NEJM* 2000:**343**:522-9.
66. Viscoli C et al. A clinical trial of estrogen-replacement therapy after ischemic stroke. *NEJM* 2001:**345**:1243-9.
67. Vickers M et al. Main morbidities recorded in the women's international study of long duration oestrogen after menopause (WISDOM): a randomised controlled trial of hormone replacement therapy in postmenopausal women *BMJ* 2007. doi:10.1136/bmj.39266.425069.
68. Schierbeck LL et al. Effect of hormone replacement therapy on cardiovascular events in recently postmenopausal women: randomised trial. *BMJ* 2012;**345**:e6409.
69. Cherry N et al. Long-term safety of unopposed estrogen used by women surviving myocardial infarction: 14-year follow-up of the ESPRIT randomised scontrolled trial *BJOG* 2014;**121**:700-705.
70. Collaborative Group on Hormonal Factors in Breast Cancer. Breast cancer and hormone replacement therapy: collaborative reanalysis of data from 51 epidemiological studies of 52,705 women with breast cancer and 108,411 women without breast cancer. *Lancet* 1997;**350**:1047-59.
71. Lyytinen H, Pukkala E, Ylikorkala O, Breast Cancer Risk in Postmenopausal Women Using Estradiol–Progestogen Therapy. *Obstet Gynecol* 2009;**113**:65-73.
72. Kerlikowske K, Miglioretti DL, Buist DS, Walker R, Carney PA. Declines in invasive breast cancer and use of postmenopausal hormone therapy in a screening mammography population. *J Natl Cancer Inst* 2007;**99:**1335-9.
73. Banks E. Hormone replacement therapy and the sensitivity and specificity of breast cancer screening: a review. *J Med Screen* 2001;**8**:29-34.
74. Collaborative Group on Hormonal Factors in Breast Cancer. Menarche, menopause, and breast cancer risk: individual participant meta-analysis, including 118 964 women with breast cancer from 117 epidemiological studies. *Lancet Oncology* 2012;**13**:1141–1151.
75. Kennedy DL, Baum C, Forbes MB. Noncontraceptive estrogens and progestins. Use patterns over time. *Obstet Gynecol* 1985;**65**:441-6.
76. Wysowski DK, Golden L, Burke L. Use of menopausal estrogens and medroxyprogesterone in the United States, 1982-1992. *Obstet Gynecol* 1995;**85**:6-10.
77. Wysowski DK, Governale LA. Use of menopausal hormones in the United States, 1992 through June, 2003. *Pharmacoepidemiol Drug Safe* 2005;**14**:171-176.
78. Ettinger B, Wang SM, Leslie RS et al. Evolution of post-menopausal hormone therapy between 2002 and 2009. *Menopause* 2012;**19**;610-615
79. Steinkellner AR, et al. A decade of postmenopausal hormone therapy prescribing in the United States. *Menopause* 2012;**19**:616-21.
80. Jewett P et al. Trends of postmenopausal estrogen plus progestin prevalence in the United States between 1970 and 2010. *Obstet Gynecol* 2014;**124**(4):727-33.
81. Townsend J. Hormone replacement therapy: Assessment of present use, costs and trends. *Br J Gen Pract* 1998;**48**:955-8.

82. Watson J, Wise L, Green J. Prescribing of hormone therapy for menopause, tibolone, and bisphosphonates in women in the UK between 1991 and 2005. *Eur J Clin Pharmacol* 2007;**63**:843-49.
83. https://digital.nhs.uk/data-and-information/publications/statistical/prescriptions-dispensed-in-the-community/prescriptions-dispensed-in-the-community-statistics-for-england-2006-2016 (Accessed 19 May 2019).
84. De P, Neutel CI, Olivotto I, Morrison H. Breast Cancer Incidence and Hormone Replacement Therapy in Canada. *J Natl Cancer Inst* 2010;**102**:1489-1495.
85. IARC, Evaluation of Carcinogenic Risks to Humans. Vol72: Hormonal contraception and postmenopausal hormone therapy (Lyon, France 1999).
86. EPIC Working Group on Reproductive and Hormonal Factors. Use of hormonal therapy for the menopause in 9 European countries. pp301-03. Riboli E, Lambert R (eds). IARC Sci Publ No 156. Lyon: 2002.
87. Canfell K et al. Decrease in breast cancer incidence following a rapid fall in use of hormone replacement therapy in Australia. *Med J Aust* 2008;**188**:641-44.
88. Collaborative Group on Epidemiological Studies of Ovarian Cancer. Menopausal hormone use and ovarian cancer risk: Individual participant meta-analysis of 52 epidemiological studies. *Lancet* 2015;**385**:1835-1842.
89. Costas L, et al. Hormonal contraception and postmenopausal hormone therapy in Spain: time trends and patterns of use. *Menopause* 2015;**22**(10):1138-46.
90. Baladé Martínez L et al. Utilization of hormone replacement therapy in Spain: Trends in the period 2000-2014. *Med Clin (Barc)* 2016;**147**(7):287-92.
91. Farmer R, Fenton A. Time trends in breast cancer and menopause hormone therapy use in New Zealand. *Climacteric* 2016;**19**(1):42-48.
92. Strongman H, Williams R, Meeraus W et al. Limitations of health research with restricted data collection from UK primary care. *Pharmacoepidemiol Drug Saf*. 2019:1-11.
93. Goss PE, Ingle JN, Ales-Martinez JE, et al. Exemestane for breast cancer prevention in postmenopausal women. *New Engl J Med* 2011:**364**:2381-91.
94. Cuzick J, Sestak I, Forbes JF, et al. Anastrozole for prevention of breast cancer in high-risk postmenopausal women (IBIS-II): an international, double-blind, randomised placebo-controlled trial. *Lancet* 2014;**383**:1041-48.
95. Cuzick J, Sestak I, Bonanni B, et al. Selective oestrogen receptor modulators in prevention of breast cancer: an updated meta-analysis of individual participant data. *Lancet* 2013;**381**:1827-34.
96. Early Breast Cancer Trialists' Collaborative Group (EBCTCG). Relevance of breast cancer hormone receptors and other factors to the efficacy of adjuvant tamoxifen: patient-level meta-analysis of randomised trials. *Lancet* 2011;**378**:771-84.

Trends in MHT use in Western countries

Over the last 50 years the prevalence of MHT use has varied considerably.[75-83] Statistics on trends in use over this time period are available for the USA[75-80] and the UK.[81-83] For other Northern European countries, Canada, Australia and New Zealand some data on trends in MHT use are available, often just for the last few decades[84-91] (see pp14-15 for the references).

USA (Figure A): MHT use increased in the early 1970s but declined in the late 1970s following reports of increased risks of endometrial cancer associated with use of oestrogen-only preparations. In the late 1980s use began to increase again and continued to do so during the 1990s, but halved abruptly in the early 2000s. It stabilized in the 2010s, with about 5 million users each year.

UK (Figure B): There was little use of MHT until the late 1980s. Use increased rapidly during the 1990s, but halved abruptly in the early 2000s. It stabilised in the 2010s, with about one million users each year.

Other Western countries: In Canada trends in MHT use appear broadly similar to those in the USA.[84] In Northern and Western Europe, Australia and New Zealand trends in MHT use are broadly similar to those in the UK.[85-91]

Use of MHT in Western countries since 1970
Overall in Western countries there have been about 600 million person-years of MHT use in the 50 years since 1970 (Figure 1 and Appendix p27). In the 2010s there were about 12 million users each year.

Trends in HT use in the USA and UK since 1970.

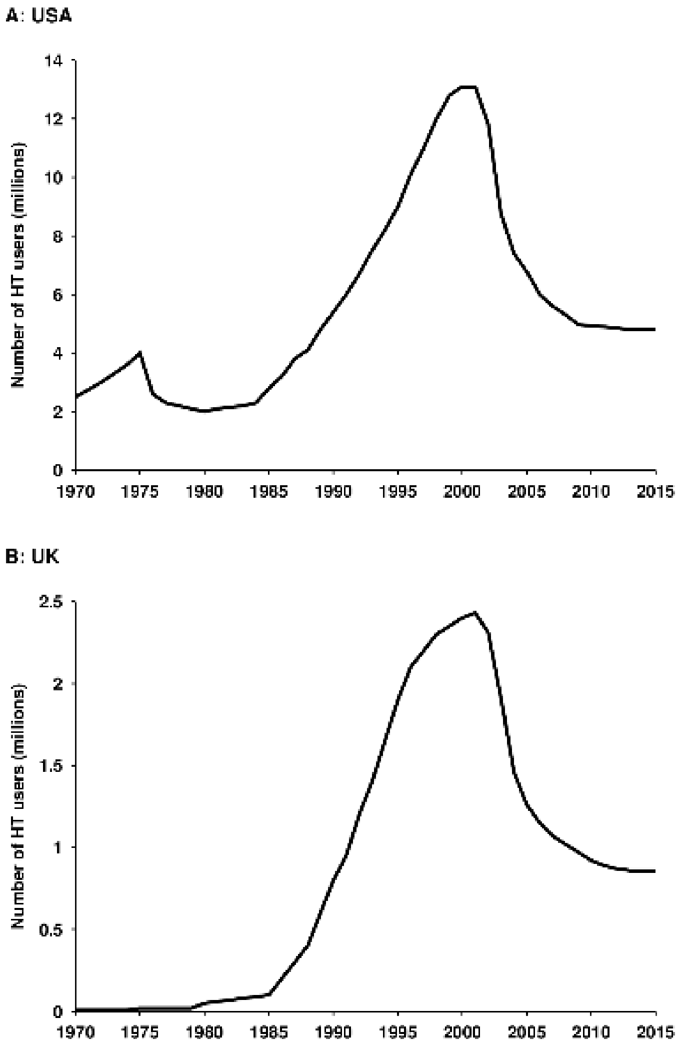

Breast cancer risk in never-users of MHT

We used age-specific breast cancer registration rates in England in 2015† to estimate breast cancer risk, by age, expressed as risk per 100 women over a 5 year period. The rates in England are typical of rates in Northern Europe, Western Europe, North America, Australia, and New Zealand.‡ Hence, among all women of average weight in Western countries, breast cancer risk (ie., the percent diagnosed with breast cancer) between age 50 and 54 is 1.4%; between age 50 and 59 is 2.8%; between age 55 and 64 is 3.1%; and between age 50 and 69 is 6.6% (Table S3).

The attributable fraction of breast cancer associated with MHT use in 2015 was estimated by combining data on MHT use with the RRs associated with current and past use (figure 2). In 2015 the attributable fraction in each age group was approximately 5%, and this value was used to estimate breast cancer risk, by age, in never-users of MHT.

Among never-users of MHT of average weight in Western countries, the estimated breast cancer risk between age 50 and 54 is 1.3%, between age 50 and 59 is 2.7%, between age 55 and 64 is 3.0%, and between age 50 and 69 is 6.3% (Table S3). These rates were assumed to apply to never-users of mean BMI 27 kg/m^2 (overweight women). Based on the data in figure 6 the breast cancer incidence rates in lean never-users (BMI <25 kg/m^2) were taken to be 20% lower than in overweight women and the rates in obese women (BMI ≥30 kg/m^2) were taken to be 14% higher than in overweight women.

The rates in Table S3 would apply to those aged about 50 years at menopause, as this is the average age at menopause found here for women in Western countries. Some estimates of breast cancer incidence were made for women whose age at menopause was 45 years and 55 years, respectively. Breast cancer risk increases by a factor of 1.029 for every year older at menopause,[74] and breast cancer incidence rates at every age in never-users with a menopausal age of 45 were assumed to be 13% (ie., $1\text{-}1.029^{-5}$) lower than the average, and in never-users with a menopausal age of 55 were assumed to be 13% higher than the average. (In 2015 breast cancer incidence rates from age 45 to 49 years in all never-users was taken to be 1.1%†.)

Table S3: Estimated breast cancer risk for women of average weight in Western countries in 2015

	**Breast cancer risk over 5 years**			
	**50-54**	**55-59**	**60-64**	**65-69**
Breast cancer risk in all women	1.40%	1.40%	1.71%	2.09%
Estimated risk in never-users of MHT	1.33%	1.33%	1.63%	1.99%

† https://www.ons.gov.uk/peoplepopulationandcommunity/healthandsocialcare/conditionsanddiseases/datasets/cancerregistrationstatisticscancerregistrationstatisticsengland

‡ ci5.iarc.fr/CI5plus/Pages/graph2_sel.aspx

Calculating absolute excess risk of breast cancer in MHT users

All the prospective studies that were analysed were of women in Western countries. The absolute excess risks in such countries that would nowadays be associated with particular patterns of MHT use can be calculated by combining the relative risks among current users and past users in these prospective studies with estimates of the absolute breast cancer rates that would nowadays be seen in never-users of MHT. These never-user rates can be estimated either from the rates in never-users in the prospective studies themselves or, yielding similar estimates, from the breast cancer incidence rates typically seen at ages 50-69 in cancer registries in Western countries for women of average weight (by subtracting 5% from the overall rates to allow approximately for the fraction attributable to MHT use: Appendix p17).

Among women in the prospective studies who used MHT the mean age at menopause was 50 years and the mean age at starting MHT was also 50 years. As the duration of MHT use for the current users in the prospective studies was typically about 10 years, starting at about the time of menopause, we present calculations for 10 years use of oestrogen-progestagen MHT, starting at age 50. But, as shorter durations of MHT use might now be more usual we also present calculations for 5 years use of oestrogen-progestagen MHT, again starting at age 50. As there is limited information on the residual excess risk more than 15 years after cessation, our calculations are of the excess at ages 50-69 years. For these calculations we use the relative risks for women who started oestrogen-progestagen MHT at age 45-54 years that are presented on p41 of this appendix. The relative risks for current users during years <1, 1-4 and 5-9 are 1.2, 1.7 and 2.0, respectively, and the relative risks for past users who had stopped after 5 years or 10 years of use are 1.18 and 1.36, respectively. The results of these calculations (Appendix Tables S11 and S12, pp24-25) are for any oestrogen-progestagen MHT.

Figure 4 shows that the risks for intermittent and for daily progestagens were, respectively, 1/6 less and 1/6 more than the average for all oestrogen-progestagen MHT. For statistical stability, the absolute risks for oestrogen-plus-intermittent-progestagen and for oestrogen-plus-daily-progestagen utilise this, and are estimated as being 1/6 less and 1/6 more than the absolute risk for any oestrogen-progestagen MHT and results are shown in figure 7.

The excess risks for oestrogen-progestagen MHT do not depend much on adiposity (figure 6, Appendix p48), but the excess risks for oestrogen-only MHT depend strongly on adiposity (mimicking the effects of obesity on breast cancer incidence). Among obese women, therefore, oestrogen-only MHT has little additional effect on breast cancer risk, whereas among lean women (BMI <25 kg/m^2, mean 22 kg/m^2) it increases their breast cancer risk to approximately that of obese women. Although these are the patterns suggested by the relative risks, for statistical stability the excess absolute risks from oestrogen-only MHT were estimated as being one-third of the excess absolute risks for oestrogen-progestagen MHT (figure 7).

The excess absolute risks of breast cancer to age 70 years were also estimated for women whose age at menopause and age at starting any oestrogen-progestagen MHT was 45, and for those whose age at menopause and age at starting any oestrogen-progestagen MHT was 55.

Table S4: Prospective study findings compared with retrospective study findings – RRs for particular durations of current use of particular MHT types versus never-use of MHT

Fully adjusted relative risks for current MHT users versus never-users

	Prospective studies RR (95%CI)	Retrospective studies RR (95%CI)	Ratio of RRs (95%CI): retrospective versus prospective studies
**Current use of oestrogen-only MHT, by duration of use**			
<1 year	1.08 (0.86-1.35)	0.84 (0.69-1.02)	0.78 (0.58-1.05)
1-4 years	1.17 (1.09-1.25)	0.93 (0.85-1.03)	0.80 (0.71-0.90)
5-9 years	1.22 (1.16-1.28)	1.15 (1.06-1.26)	0.94 (0.85-1.04)
10-14 years	1.43 (1.37-1.50)	1.20 (1.09-1.31)	0.84 (0.75-0.93)
15+ years	1.58 (1.50-1.67)	1.18 (1.09-1.27)	0.74 (0.68-0.82)
**Unweighted average**	**1.30**	**1.06**	**0.8**
**Current use of oestrogen-progestagen MHT, by duration of use**			
<1 year	1.20 (1.01-1.43)	0.81 (0.65-1.01)	0.68 (0.51-0.89)
1-4 years	1.60 (1.52-1.69)	1.11 (1.01-1.21)	0.69 (0.62-0.77)
5-9 years	1.97 (1.89-2.04)	1.68 (1.54-1.84)	0.86 (0.78-0.94)
10-14 years	2.26 (2.16-2.36)	2.06 (1.85-2.29)	0.91 (0.81-1.02)
15+ years	2.51 (2.34-2.68)	2.07 (1.84-2.34)	0.83 (0.72-0.95)
**Unweighted average**	**1.91**	**1.55**	**0.8**

Table S5: Sensitivity analyses in prospective studies – effects on fully adjusted relative risks of additional adjustment for five other factors

	Relative risk (95%CI) for 5-14 (mean=9) years current use versus never-use of MHT	
	Oestrogen-only	Oestrogen-progestagen
**Stratified by study, centre within study, age and BMI, adjusted by parity, age at first birth, alcohol, and breast cancer family history, and allowing for age at menopause (as in the main analyses)**	**1.33 (1.28-1.37)**	**2.08 (2.02-2.15)**
As in the main analyses, but with additional adjustment for:		
Ethnic origin	1.28 (1.23-1.33)	2.06 (1.99-2.13)
Years of education	1.32 (1.28-1.37)	2.08 (2.02-2.14)
Age at menarche	1.32 (1.28-1.37)	2.08 (2.02-2.15)
Height	1.32 (1.28-1.37)	2.08 (2.02-2.15)
Past oral contraceptive use	1.33 (1.28-1.38)	2.09 (2.02-2.15)
All of the above	1.28 (1.23-1.33)	2.06 (2.00-2.13)

Table S6: Sensitivity analyses in prospective studies – effect of restricting analyses to studies with no missing values for the adjustment variables, and effects in these restricted analyses of additional adjustment for five other factors

	Relative risk (95%CI) for 5-14 (mean=9) years current use versus never-use of MHT	
	Oestrogen-only	Oestrogen-progestagen
**Stratified by study, centre within study, age and BMI, adjusted by parity, age at first birth, alcohol, and breast cancer family history, and allowing for age at menopause (as in the main analyses)**	**1.32 (1.27-1.38)**	**2.03 (1.96-2.10)**
As in the main analyses, but with additional adjustment for:		
Ethnic origin	1.32 (1.27-1.38)	2.03 (1.96-2.10)
Years of education	1.32 (1.27-1.38)	2.03 (1.96-2.10)
Age at menarche	1.32 (1.27-1.38)	2.03 (1.96-2.10)
Height	1.32 (1.27-1.38)	2.03 (1.96-2.10)
Past oral contraceptive use	1.33 (1.27-1.38)	2.04 (1.97-2.11)
All of the above	1.32 (1.27-1.37)	2.03 (1.97-2.10)

Table S7: Sensitivity analyses in prospective studies – effects of various cut-offs between last recorded MHT use and index date in current users

Fully adjusted relative risks for current MHT users versus never-users.

	Numbers of cases never-use / oestrogen-only / oestrogen-progestagen	Relative risk (95%CI) for 5-14 (mean=9) years current use vs never-use of MHT	
		Oestrogen-only	Oestrogen-progestagen
No cut-off	53072 / 6972 / 12666	1.28 (1.24-1.32)	1.90 (1.85-1.95)
Cut-off at 6 years	53072 / 5281 / 9250	1.32 (1.27-1.36)	2.06 (2.00-2.12)
**Cut-off at 5 years (as in main analyses)**	**53072 / 4869 / 8318**	**1.33 (1.28-1.37)**	**2.08 (2.02-2.15)**
Cut-off at 4 years	53072 / 4421 / 7481	1.36 (1.31-1.41)	2.18 (2.11-2.25)
Cut-off at 3 years	53072 / 3702 / 6184	1.33 (1.28-1.38)	2.15 (2.08-2.23)
Cut-off at 2 years	53072 / 2926 / 4846	1.33 (1.27-1.39)	2.19 (2.11-2.28)

Table S8: Sensitivity analyses in prospective studies – effects of various exclusions

Fully adjusted relative risks for current MHT users versus never-users.

	Relative risk (95%CI) for 5-14 (mean=9) years current use vs never-use of MHT	
	Oestrogen-only	Oestrogen-progestagen
**Main analyses**	**1.33 (1.28-1.37)**	**2.08 (2.02-2.15)**
Excluding cancers in the first year after last record about MHT use	1.31 (1.26-1.37)	2.05 (1.98-2.12)
Excluding the study with most cases[54]	1.32 (1.26-1.38)	2.03 (1.94-2.11)
Excluding the 10 studies with <500 cases[4,7,9,10,19,33,35,36,52,58]	1.33 (1.28-1.38)	2.08 (2.02-2.14)

Table S9: Results in prospective studies by dose of oral oestrogen-only constituents

Fully adjusted relative risks for current MHT users versus never-users.

	**Exposed cases (n)**	**Relative risk (95%CI) for 5-14 years current use of MHT versus never-use**
**Equine oestrogen**		
300 mcg/day	69	1.94 (1.45-2.58)
625 mcg/day	1154	1.34 (1.25-1.43)
>625 mcg/day	491	1.32 (1.19-1.47)
**Oestradiol**		
1 mg/day	246	1.34 (1.15-1.57)
2 mg/day	162	1.29 (1.07-1.55)

Table S10: Results in prospective studies for oestrogen-progestagen MHT, by hormonal constituents

Fully adjusted relative risks for current MHT users versus never-users.

	**Relative risk (95%CI) for 5-14 (mean=9) years current use of MHT versus never-use**		
	**Levonorgestrel**	**Norethisterone acetate**	**Medroxyprogesterone acetate**
**By frequency of addition of progestagen**			
Daily<br>*%obese/%daily*	insufficient	2.43 (2.28-2.59)<br>*12/100*	2.24 (2.10-2.40)<br>*14/100*
Intermittent<br>*%obese/%daily*	2.12 (2.00-2.26)<br>*11/0*	1.81 (1.64-1.99)<br>*11/0*	1.69 (1.50-1.91)<br>*12/0*
**By region**			
USA<br>*%obese/%daily*	insufficient	insufficient	1.83 (1.67-2.01)<br>*15/53*
Europe, except Scandinavia<br>*%obese/%daily*	2.14 (2.01-2.27)<br>*12/<1*	2.21 (2.10-2.33)<br>*13/70*	2.23 (2.07-2.39)<br>*13/87*
Scandinavia<br>*%obese/%daily*	insufficient	2.19 (1.79-2.69)<br>*7/53*	insufficient
**By oestrogenic type**			
Equine oestrogen<br>*%obese/%daily*	2.18 (2.04-2.33)<br>*12/0*	insufficient	2.11 (1.99-2.24)<br>*14/80*
Oestradiol<br>*%obese/%daily*	1.99 (1.73-2.28)<br>*10/3*	2.20 (2.09-2.33)<br>*12/70*	1.64 (1.34-2.01)<br>*13/24*

Table S11: Estimated excess incidence of breast cancer before age 70 in Western countries with 5 years use of any oestrogen-progestagen MHT, starting at age 50 years

This table illustrates the *absolute* excess risks that would correspond to the excess *relative* risks associated with 5 years oestrogen-progestagen MHT in a population where never-users have breast cancer incidence 5% lower than in England in 2015. Absolute risks if never-users had slightly higher or lower rates than this should, correspondingly, be slightly higher or lower.

	**Background: 5-year incidence in never-users† (B)**	**Excess incidence of breast cancer associated with 5 years use of oestrogen-progestagen MHT, starting at age 50**		
		**Relative risk, current or ex- vs never-users‡ (RR)**	**Proportional excess risk (RR-1)**	**Absolute excess incidence, (B) times (RR-1)**
50 to 54 (current user)	1.33 %	1.60	0.60	0.80 %
55 to 59 (past user)	1.33 %	1.18	0.18	0.24 %
60 to 64 (past user)	1.63 %	1.18	0.18	0.29 %
65 to 69 (past user)	1.99 %	1.18	0.18	0.36 %
**50 to 69 (current + past)**				**1.7 % (about 1 in 60)**

For methods, see Appendix p18.

† Age-specific breast cancer risks in never-users of MHT (Appendix p17; the incidence rate in never-users from age 45 to 49 is taken to be 1.1%, but this is not used in Table S11).

‡ RRs are estimated from the findings for women who had started using oestrogen-progestagen at ages 45-54 (Appendix p41). The RR for current users with 1-4 years of use was 1.66 (95% CI 1.55-1.78), and a RR of 1.6 is used in Table S11 for ages 50-54. The RR for past users with 3-7 (mean=5) years of use was 1.18 (1.12-1.24), and a RR of 1.18 is used in Table S11 for ages 55-69.

The calculations in Table S11 are directly relevant to the excess breast cancer incidence before age 70 associated with 5 years of oestrogen-progestagen MHT following menopause at age 50. Similar calculations can be performed of the excess breast cancer incidence before age 70 associated with 5 years of MHT following menopause at age 45 or at age 55, again using RR=1.6 in current users and RR=1.18 in past users, although the incidence rates in never-users must be decreased by 13% or increased by 13%, respectively, to allow for the effects of earlier or later age at menopause (Appendix p17). With these assumptions, the excess breast cancer risk before age 70 associated with 5 years of MHT is 1.6% for menopause and starting at 45, 1.7% for menopause and starting at 50 (Table S11) and 1.7% for menopause and starting at 55. Thus, there is little difference in the absolute excess incidence by age 70 associated with starting 5 years of MHT use at ages 45, 50 or 55.

Table S12: Estimated excess incidence of breast cancer before age 70 in Western countries with 10 years use of any oestrogen-progestagen MHT, starting at age 50 years

This table illustrates the *absolute* excess risks that would correspond to the excess *relative* risks associated with 10 years oestrogen-progestagen MHT in a population where never-users have breast cancer incidence 5% lower than in England in 2015. Absolute risks if never-users had slightly higher or lower rates than this should, correspondingly, be slightly higher or lower.

	**Background: 5-year incidence in never-users[†] (B)**	**Excess incidence of breast cancer associated with 10 years use of oestrogen-progestagen MHT, starting at age 50**		
		**Relative risk, current or ex- vs never-users[‡] (RR)**	**Proportional excess risk (RR-1)**	**Absolute excess incidence, (B) times (RR-1)**
50 to 54 (current user)	1.33 %	1.60	0.60	0.80 %
55 to 59 (current user)	1.33 %	1.96	0.96	1.28 %
60 to 64 (past user)	1.63 %	1.36	0.36	0.59 %
65 to 69 (past user)	1.99 %	1.36	0.36	0.72 %
**50 to 69 (current + past)**				**3.4 % (about 1 in 30)**

For methods, see Appendix p18.

‡ Age-specific breast cancer risks in never-users of MHT (Appendix p17).

‡ RRs are estimated from the findings for women who had started using oestrogen-progestagen at ages 45-54 (Appendix p41). The RR for current users with 1-4 years of use was 1.66 (95% CI 1.55-1.78), and a RR of 1.6 is used in Table S12 for ages 50-54. The RR for current users with 5-9 years of use was 1.96 (95% CI 1.87-2.05), and a RR of 1.96 is used in Table S12 for ages 55-59. The RR for past users with 8-12 (mean=10) years of use was 1.36 (1.29-1.44), and a RR of 1.36 is used in Table S12 for ages 60-69.

Table S13: Data used for figure 7 on the effect of 5 years or 10 years MHT use, starting from age 50, and of adiposity on 20-year breast cancer incidence rates from 50 to 69 (%)

Panel A: Oestrogen-plus-daily-progestagen

	**Breast cancer incidence (%) from age 50 years to:**			
	**age 54**	**age 59**	**age 64**	**age 69**
Never-use	1.33	2.66	4.29	6.29
Duration of MHT use				
5 years	2.26	3.87	5.84	8.25
10 years	2.26	5.15	7.43	10.3

Panel B: Oestrogen-plus-intermittent-progestagen

	**Breast cancer incidence (%) from age 50 years to:**			
	**age 54**	**age 59**	**age 64**	**age 69**
Never-use	1.33	2.66	4.29	6.29
Duration of MHT use				
5 years	2.02	3.55	5.43	7.73
10 years	2.02	4.49	6.62	9.23

Panel C: Oestrogen-only (no progestagen)

	**Breast cancer incidence (%) from age 50 years to:**			
	**age 54**	**age 59**	**age 64**	**age 69**
Never-use	1.33	2.66	4.29	6.29
Duration of MHT use				
5 years	1.60	3.01	4.73	6.84
10 years	1.60	3.37	5.20	7.43

Panel D: Never-users, by body mass index (BMI)

	**Breast cancer incidence (%) from age 50 years to:**			
	**age 54**	**age 59**	**age 64**	**age 69**
Lean (BMI<25)	1.08	2.15	3.47	5.09
Overweight (BMI 25-29)	1.33	2.66	4.29	6.29
Obese (BMI ≥30)	1.52	3.03	4.89	7.17

Table S14: Relevance of age to the effects of obesity on breast cancer incidence in postmenopausal never-users of MHT

**Age at diagnosis**	**Relative risk (95%CI), obese (BMI ≥30 kg/m^2) vs lean (BMI <25 kg/m^2) †, in never-users of MHT**
**50-59**	1.26 (1.19-1.34)
**60-64**	1.36 (1.29-1.43)
**65-69**	1.44 (1.37-1.52)
**70-74**	1.56 (1.47-1.66)

† Mean BMI in these two BMI categories: obese 34.3 kg/m^2 and lean 22.6 kg/m^2

Table S15: Estimated person-years of MHT use in 21 Western countries, 1970-2019, and eventual (past + future) number of extra cases of breast cancer from this before age 70

Period	Person-years of MHT use (millions)		
	Any MHT	Oestrogen-only	Oestrogen-progestagen
1970-74	11	11	0
1975-79	10	9	1
1980-84	14	12	2
1985-89	30	23	7
1990-94	70	35	35
1995-99	136	68	68
2000-04	143	66	77
2005-09	66	37	29
2010-14	60	31	29
2015-19 *	60	31	29
**Total**	**600**	**323**	**277**
**Estimated extra breast cancer cases before age 70 years resulting from MHT use during 1970-2019 ****			
**Past + future**	**1,264,000**	**323,000**	**941,000**

Based on the data in figure 1, there were about 600 million person-years of MHT use in 1970-2019. Table S15 shows the total person-years of use in each 5-year period over the last 50 years and distributes this total between oestrogen-only and oestrogen-progestagen, based on ratios among controls of the 2 types of MHT.

The excess risks in figure 7 and Table S11 (appendix p24) for 5 years MHT use imply breast cancer excesses at ages 50-69 of about 3.4 per 1000 person-years use of oestrogen-progestagen MHT (1.7% divided by 5) and about 1.0 per 1000 person-years use of oestrogen-only MHT (0.5% divided by 5). Applying these excess risks to the person-years of use of each type suggests that MHT use from 1970 to 2019 would result in an estimated 1.3 million extra breast cancers, of which about one million would already have occurred, almost all since 1990. By comparison there would have been a total of about 20 million breast cancers among women in western countries since 1990. Oestrogen-progestagen MHT accounts for almost half the person-years of MHT use, but about three-quarters of the extra cases of cancer.

MHT usage halved in the early 2000s, but still about 6 million use oestrogen-only and 6 million use oestrogen-progestagen MHT. If this level of use were to continue, it would cause about 26,000 extra breast cancers per year at ages 50-69.

* 2015-19 is taken as similar to 2010-14.

** Estimates assume 1 extra case per 1000 person-years oestrogen-only MHT use and 3.4 extra cases per 1000 person-years oestrogen-progestagen MHT use.

Table S16: Meta-analysis of the randomised trials of breast cancer prevention in postmenopausal women by an aromatase inhibitor or by tamoxifen

A: 3-5 years of aromatase inhibitor vs placebo (2 AI trial reports: MAP-3 [93] & IBIS-II [94])

B: ~ 5 years of tamoxifen vs placebo (4 trials, individual-person-data meta-analysis; results for age 55+ from pers. comm. by J Cuzick with permission from trialists [95])

C: ~ 5 years of tamoxifen vs control (20 trials, individual-person-data meta-analysis [96])

Anti-oestrogen regimen evaluated	Characteristics of participants	Outcome analysed	Meta-analysis * (RR and 99% CI) in treatment period
**A: Aromatase inhibitor for 3-5 years, for primary prevention**	Postmenopausal (mean age 61), no breast cancer	Invasive ER+ breast cancer	27 vs 74<br>RR = 0.36 (0.22-0.61)<br>z = -4.5; P <0.00001<br>-23.5; 25.2 **
**B: Tamoxifen for 5 years, for primary prevention**	Age 55+ years, no breast cancer	Invasive ER+ breast cancer	76 vs 136<br>RR = 0.56 (0.39-0.80)<br>z = -4.1; P <0.0001<br>-30.0; 53.2 **
**C: Tamoxifen for 5 years, as adjuvant therapy**	Age 55+ years, with stage I/II ER+ breast cancer	New invasive cancer (with any ER status) in contralateral breast	39 vs 69<br>RR = 0.51 (0.31-0.85)<br>z = -3.5; P <0.001<br>-17.6; 26.5 †
**A, B, C (meta-analysis): Aromatase inhibitor or tamoxifen**	Postmenopausal, or age 55+ years	New invasive ER+ or contralateral breast cancer	142 vs 279<br>RR = 0.51 (0.39-0.65)<br>z = -6.9; P <10^{-10}<br>-71.1; 104.7 §

* All these trials were evenly randomised. Format of results: numbers of events by allocated treatment, RR (and its 99% CI), z = (O-E) / √V, P-value based on z, and the binomial or logrank (O-E) and its variance V. The 99% confidence limits for RR are RR multiplied or divided by exp (2.575 / √V). Analyses are by intent to treat. In the tamoxifen trials, the events counted were those within 5 years of randomisation.

** Breast cancer incidence was low in the primary prevention trials, so binomial analyses suffice and RR is taken to be the simple ratio of the number of events.

† Logrank (O-E) and its variance V; as tamoxifen delayed recurrence of ER+ disease, lengthening the follow-up for contralateral breast cancer onset, the RR and its 99% CI were calculated from the *logrank* (O-E) and V, using $\log_e$ (RR) = (O-E) / V with variance 1 / V. (NB V is equivalent to "weight" elsewhere.)

§ Total (O-E) and V, used as in Section C to calculate the incidence rate ratio, anti-oestrogen versus not.

Table S17: Trials of oestrogen-only hormone therapy (O-only HT) versus placebo

Trial name and year published (refs on p14)	Mean age at entry	Approximate years in trial and later FU	Cancers in HT group	Cancers in control group	Relative risk, RR (95%CI) with 99% CI for total	Weight, 1/var of $\log_e$(RR)*	Weight times $\log_e$(RR)
PEPI 1995[63]	~55	3+0	1/175	1/174	-	0.5**	0.0**
ERA 2000[65]	66	3+0	1/100	0/104	-	0.2**	0.5**
WEST 2001[66]	72	3+0	5/337	5/327	1.0 (0.3-3.5)	2.5	0.0
ESPRIT 2014[69]	~63	2+11	7/513	15/504	0.47 (0.19-1.15)	5.5	-4.0
DOPS 2012[68] (open control)	50	10+6	6/95	9/97	0.63 (0.23-1.78)	3.6	-1.6
Subtotal / mean in smaller trials †	62	4+7	17/ 1220	28/ 1206	0.61 (0.34-1.09), z= -1.5; 2p=0.15	12.3	-5.1
WHI trial of O-only HT vs placebo[61, 62]	64 ‡	6.8+6 §	168/ 5310	216/ 5429	0.79 (0.65-0.97), z= -2.3; 2p=0.02	95.9	-22.6
WHI O-only trial plus the smaller trials	64	6.7+6	188/ 6530	246/ 6635	0.77 (0.64-0.93), z= -2.66; 2p=0.01; 99% CI 0.60-0.99	108.2	-27.7

* Let $\log_e$(RR) have standard error se and variance v = se*se. The 95% CI for $\log_e$(RR) runs from 1.96*se below $\log_e$(RR) to 1.96*se above $\log_e$(RR). So, this 95% CI has length 3.92*se.

If a published RR has 95% CI running from x to y, then se and v can be obtained from this, because the 95% CI for $\log_e$(RR) has length $\log_e$(y) – $\log_e$(x). As this is $\log_e$(y/x), 3.92*se = $\log_e$(y/x), and v = se*se.

To get an inverse-variance-weighted average (ie, a meta-analysis) of the values of $\log_e$(RR) in several trials, the weight for each trial is 1/v, ie, the inverse of the variance of $\log_e$(RR) in that one trial, and this weight is multiplied by $\log_e$(RR) for that trial. If these products sum to T and the weights sum to W, the weighted average $\log_e$(RR) is T/W. This has variance 1/W, so it has 95% confidence limits T/W ± 1.96/√W (and 99% confidence limits T/W ± 2.575/√W).

For trials giving RR and 95% CI, this is used as above to obtain weights. For a trial giving only numbers with breast cancer, we calculate (O–E) and its variance w, noting $\log_e$(RR) is well approximated by (O–E)/w with variance 1/w, so the weight is w and the product of w and $\log_e$(RR) is (O–E).

** These small trials were reported in terms of numbers of cases, not the RR and its 95% CI.

If a trial among N women allocated a proportion P to HT and (1-P) to control, with total n breast cancer cases, then if O is the Observed number of cases in the HT group and E the corresponding Expected number of cases, E = nP. The weight w given to that trial is the variance of (O–E), so w = nP (1-P) (N-n) / (N-1) and the contribution to the final column is simply (O–E).

† Mean in all small trials of oestrogen-only HT, weighted by numbers with breast cancer.

‡ In the WHI oestrogen-only trial, half the participants were at least 20 years after the menopause when randomised.

§ In the WHI trial there were more drop-outs from HT among those allocated O-only than drop-ins among those allocated placebo.[61] The difference between those allocated active and placebo in mean duration of HT use during the trial period was about 3.2 years for the 104 vs 135 cases arising during the trial period, 5.0 years for 64 vs 81 later cases, and 3.9 years for all 168 vs 216 cases: pers. comm. from Garnet Anderson for the WHI research group.

NB In DOPS, 53% of those allocated HT stopped HT during the treatment period. All other trials had placebo control.

Table S18: Trials of oestrogen-progestagen hormone therapy (O+P HT) versus placebo

Trial name and year published (refs on p14)	Mean age at entry	Approximate years in trial and later FU	Cancers in HT group	Cancers in control group	Relative risk, RR (95%CI) with 99% CI for total	Weight, 1/var of $\log_e$(RR)*	Weight times $\log_e$(RR)
PEPI 1995[63]	~55	3+0	6/526, in 3 groups	1/174	-	1.3**	0.8**
WISDOM 2007[67]	63	1+0	5/2196	7/2189	-	3.0**	-1.0**
HERS 1998[64]	67	4+0	32/1380	25/1383	1.30 (0.77-2.19)	14.1	3.7
DOPS 2012[68] (open control)	50	10+6	18/407	17/407	1.05 (0.54-2.04)	8.7	0.5
Subtotal / mean in smaller trials †	60	6+2	57/ 4158 ‡	50/ 4153	1.14 (0.78-1.65), z= 0.8; 2p=0.44	27.1	4.0
WHI trial of O+P HT vs placebo[60. 62]	63	5.6+8 §	434/ 8506	323/ 8102	1.28 (1.11-1.48), z= 3.4; 2p=0.001	185.7	45.8
WHI O+P trial plus the smaller trials	63	5.6+7	491/ 12,664	373/ 12,255	1.26 (1.10-1.45), z= 3.41; 2p=0.001; 99% CI 1.06-1.51	212.8	49.8

* Let $\log_e$(RR) have standard error se and variance v = se*se. The 95% CI for $\log_e$(RR) runs from 1.96*se below $\log_e$(RR) to 1.96*se above $\log_e$(RR). So, this 95% CI has length 3.92*se.

If a published RR has 95% CI running from x to y, then se and v can be obtained from this, because the 95% CI for $\log_e$(RR) has length $\log_e$(y) – $\log_e$(x). As this is $\log_e$(y/x), 3.92*se = $\log_e$(y/x), and v = se*se.

To get an inverse-variance-weighted average (ie, a meta-analysis) of the values of $\log_e$(RR) in several trials, the weight for each trial is 1/v, ie, the inverse of the variance of $\log_e$(RR) in that one trial, and this weight is multiplied by $\log_e$(RR) for that trial. If these products sum to T and the weights sum to W, the weighted average $\log_e$(RR) is T/W. This has variance 1/W, so it has 95% confidence limits T/W ± 1.96/√W (and 99% confidence limits T/W ± 2.575/√W).

For trials giving RR and 95% CI, this is used as above to obtain weights. For a trial giving only numbers with breast cancer, we calculate (O–E) and its variance w, noting $\log_e$(RR) is well approximated by (O–E)/w with variance 1/w, so the weight is w and the product of w and $\log_e$(RR) is (O–E).

** These small trials were reported in terms of numbers of cases, not the RR and its 95% CI.

If a trial among N women allocated a proportion P to HT and (1-P) to control, with total n breast cancer cases, then if O is the Observed number of cases in the HT group and E the corresponding Expected number of cases, E = nP. The weight w given to that trial is the variance of (O–E), so w = nP (1-P) (N-n) / (N-1) and the contribution to the final column is simply (O–E).

† Mean in all small trials of oestrogen-progestagen HT, weighted by numbers with breast cancer.

‡ To maintain balance, only 1/3 of the 6/526 in the 3 O+P groups in PEPI are included in the total for small trials.

§ In the WHI trial, there were more drop-outs from HT among those allocated O+P than drop-ins among those allocated placebo.[60] The difference between those allocated active and placebo in mean duration of HT use during the trial period was about 3.0 years for the 206 vs 155 cases arising during the trial period, 4.3 years for 228 vs 168 later cases, and 3.6 years for all 168 vs 216 cases: pers. comm. from Garnet Anderson for the WHI research group.

NB In DOPS, 53% of those allocated HT stopped HT during the treatment period. All other trials had placebo control.

Figure S1: Study-specific relative risks for breast cancer in ever vs never-users of MHT

Fully adjusted relative risks for MHT users versus never-users

	Number of cases (never/ever MHT use)	Relative risk (95% or 99% CI)
**European prospective studies**		
Persson	27 / 129	0.55 (0.26, 1.16)
Oxford FPA	9 / 2	1.28 (0.10, 16.7)
Danish Nurses	90 / 66	3.44 (1.99, 5.96)
EPIC	1798 / 1488	1.41 (1.26, 1.59)
Icelandic cohort	211 / 331	1.76 (1.36, 2.29)
NOWAC	303 / 310	1.74 (1.35, 2.25)
Southern Sweden	291 / 127	1.24 (0.88, 1.75)
Norway/Sweden Women's Health	91 / 121	1.25 (0.82, 1.91)
Million Women Study	20961 / 22061	1.28 (1.25, 1.32)
CPRD	17835 / 12286	1.25 (1.20, 1.30)
Generations Study	321 / 380	1.11 (0.89, 1.39)
Norwegian Breast Screening Study	1615 / 1881	1.31 (1.19, 1.45)
**All Europe**	**43552 / 39182**	**1.29 (1.27, 1.31)**
**North American prospective studies**		
Kaiser Permanente	19 / 80	1.06 (0.38, 2.93)
Baltzell	35 / 13	1.29 (0.35, 4.79)
BCDDP Follow-up Study	919 / 1428	1.03 (0.91, 1.17)
SOF	206 / 167	1.08 (0.80, 1.46)
Nurses' Health Study	2500 / 4616	1.26 (1.17, 1.35)
CPS-II Nutrition	967 / 1563	1.14 (1.01, 1.28)
Multiethnic Cohort	1101 / 1303	1.30 (1.16, 1.46)
NIH-AARP	1313 / 1843	1.42 (1.27, 1.58)
Women's Health Initiative Observational Study	771 / 2135	1.29 (1.14, 1.46)
PLCO	724 / 183	1.48 (1.14, 1.91)
California Teachers	846 / 2711	1.24 (1.10, 1.39)
Nurses' Health Study II	119 / 351	0.76 (0.55, 1.06)
**All North America**	**9520 / 16393**	**1.24 (1.20, 1.28)**
**All prospective studies**	**53072 / 55575**	**1.29 (1.27, 1.30)**
**European and Australasian retrospective studies**		
Vessey	393 / 56	1.20 (0.70, 2.07)
Ewertz	473 / 279	1.41 (1.04, 1.91)
CRC/ICRF	73 / 35	1.04 (0.48, 2.26)
La Vecchia	1344 / 176	1.23 (0.91, 1.67)
Levi	97 / 55	1.30 (0.68, 2.48)
GESBC	60 / 22	0.72 (0.27, 1.96)
Magnusson	1715 / 898	1.48 (1.25, 1.74)
Polish Breast Cancer Study	961 / 303	1.46 (1.13, 1.89)
MARIE	938 / 2235	1.40 (1.27, 1.55)
CLEAR	354 / 463	1.52 (1.13, 2.04)
**All Europe & Australasia**	**6408 / 4522**	**1.41 (1.33, 1.49)**
**North American retrospective studies**		
Leisure World	33 / 73	0.94 (0.48, 1.84)
BCDDP Case-Control Study	804 / 489	0.93 (0.75, 1.15)
Nomura	101 / 97	0.71 (0.41, 1.24)
CASH	336 / 266	1.06 (0.79, 1.43)
Clarke	251 / 57	0.95 (0.60, 1.51)
Long Island Study	570 / 162	1.14 (0.82, 1.59)
Asian American I	77 / 29	1.71 (0.74, 3.94)
Baltzell	211 / 194	0.72 (0.50, 1.03)
Western New York	330 / 99	0.93 (0.60, 1.43)
Daling	6 / 14	1.67 (0.23, 11.8)
Ross/Pike	800 / 911	1.13 (0.94, 1.36)
Stanford/Habel	179 / 185	0.87 (0.60, 1.28)
Yang/Gallagher	339 / 258	1.08 (0.79, 1.49)
WISH	31 / 30	2.09 (0.61, 7.11)
4-state Study	3182 / 1576	1.10 (0.98, 1.23)
CARE	716 / 1380	0.92 (0.78, 1.09)
Friedenreich	330 / 315	0.77 (0.57, 1.05)
Kreiger	1070 / 763	1.03 (0.86, 1.23)
LIBCSP	603 / 211	0.93 (0.69, 1.25)
Newcomb	1648 / 1924	1.28 (1.13, 1.45)
San Francisco Bay Area	580 / 751	1.08 (0.88, 1.31)
Asian American II	269 / 254	1.02 (0.72, 1.45)
PACE	267 / 703	1.24 (0.96, 1.60)
WISE	457 / 379	0.99 (0.78, 1.26)
**All North America**	**13190 / 11120**	**1.06 (1.02, 1.10)**
**All retrospective studies**	**19598 / 15642**	**1.15 (1.12, 1.19)**
**All studies**	**72670 / 71217**	**1.26 (1.24, 1.28)**

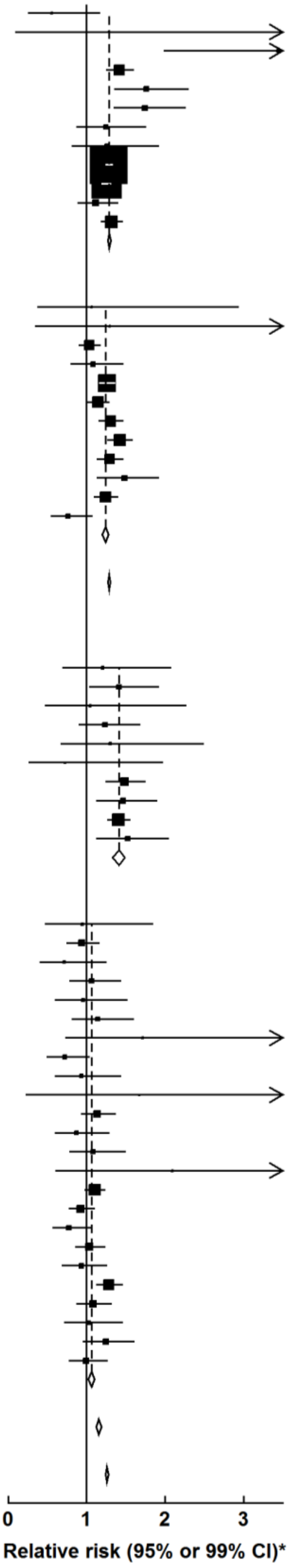

***99% CIs for study-specific results, 95% CIs for totals.**

Figure S2: Study-specific relative risks for breast cancer in ever versus never-users of MHT, in women with known MHT type last used, duration of use and time since last use

Fully adjusted relative risks for MHT users versus never-users

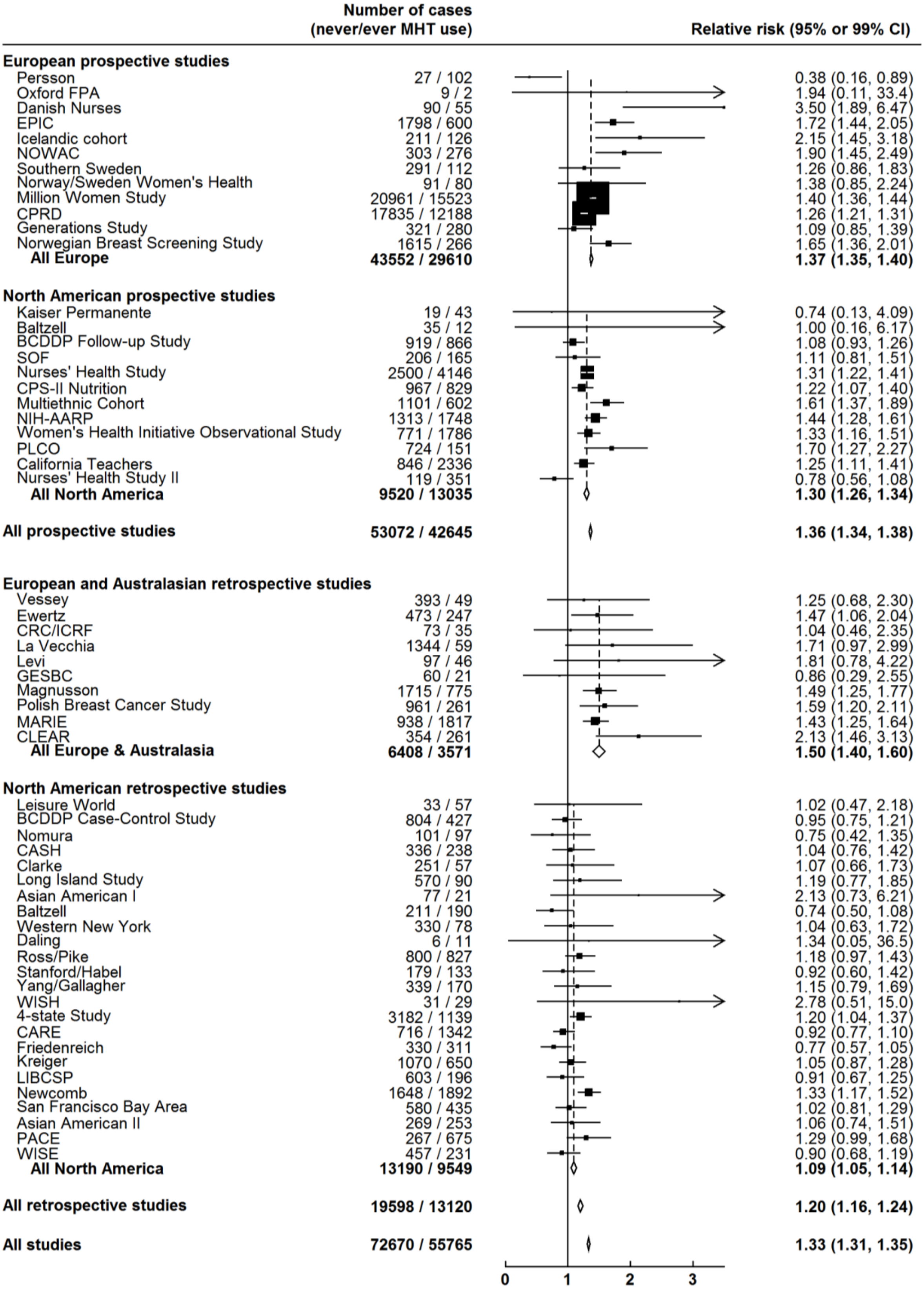

*99% CIs for study-specific results, 95% CIs for totals.

Figure S3: Study-specific relative risks of breast cancer in current users versus never-users of MHT, by type of MHT last used and study design

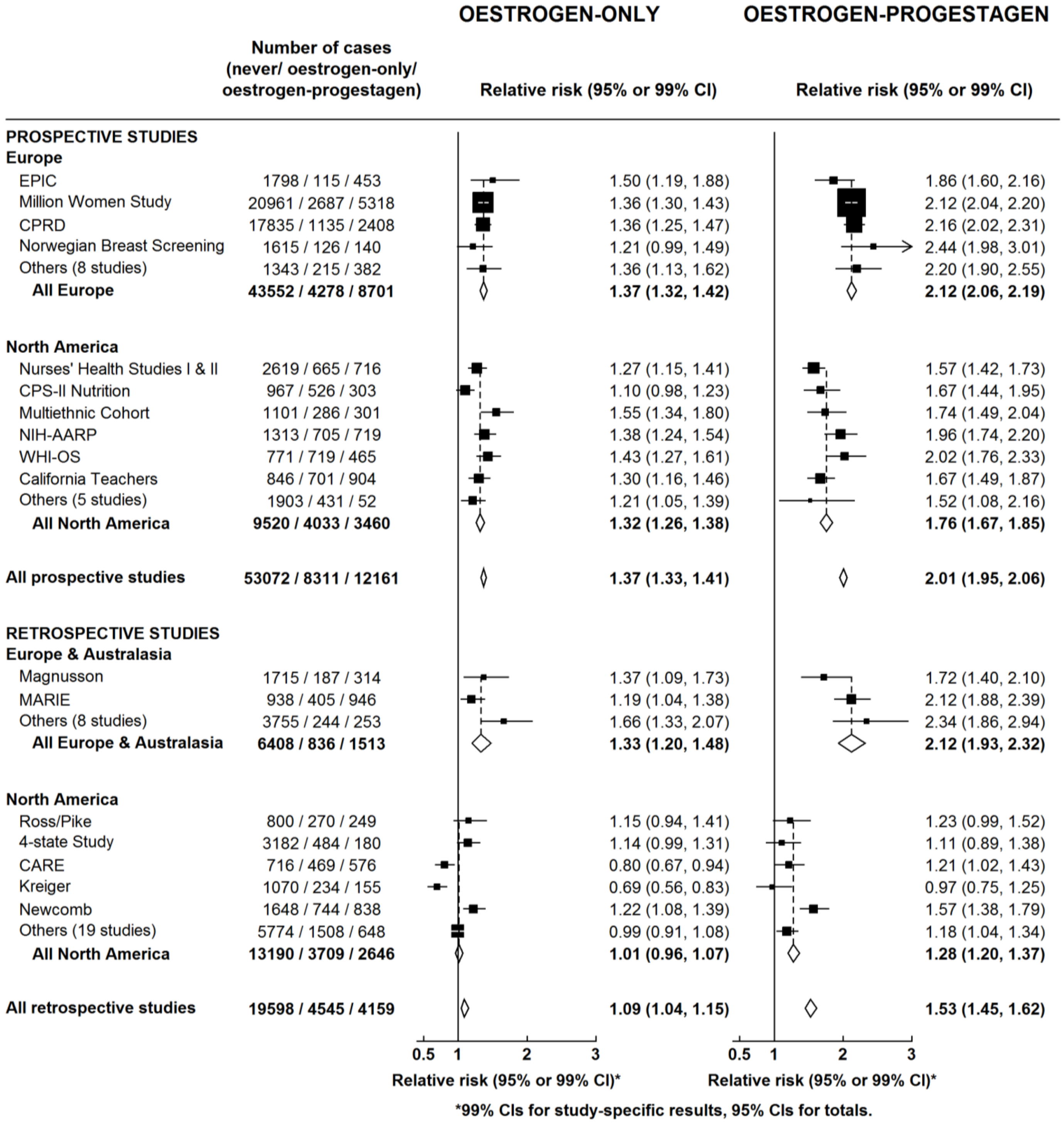

Heterogeneity, prospective vs retrospective studies: $p<0.0001$ for each type of MHT

Figure S4: Study-specific relative risks of breast cancer in current users of MHT during years 5-14 versus never-users, by type of MHT last used and study design

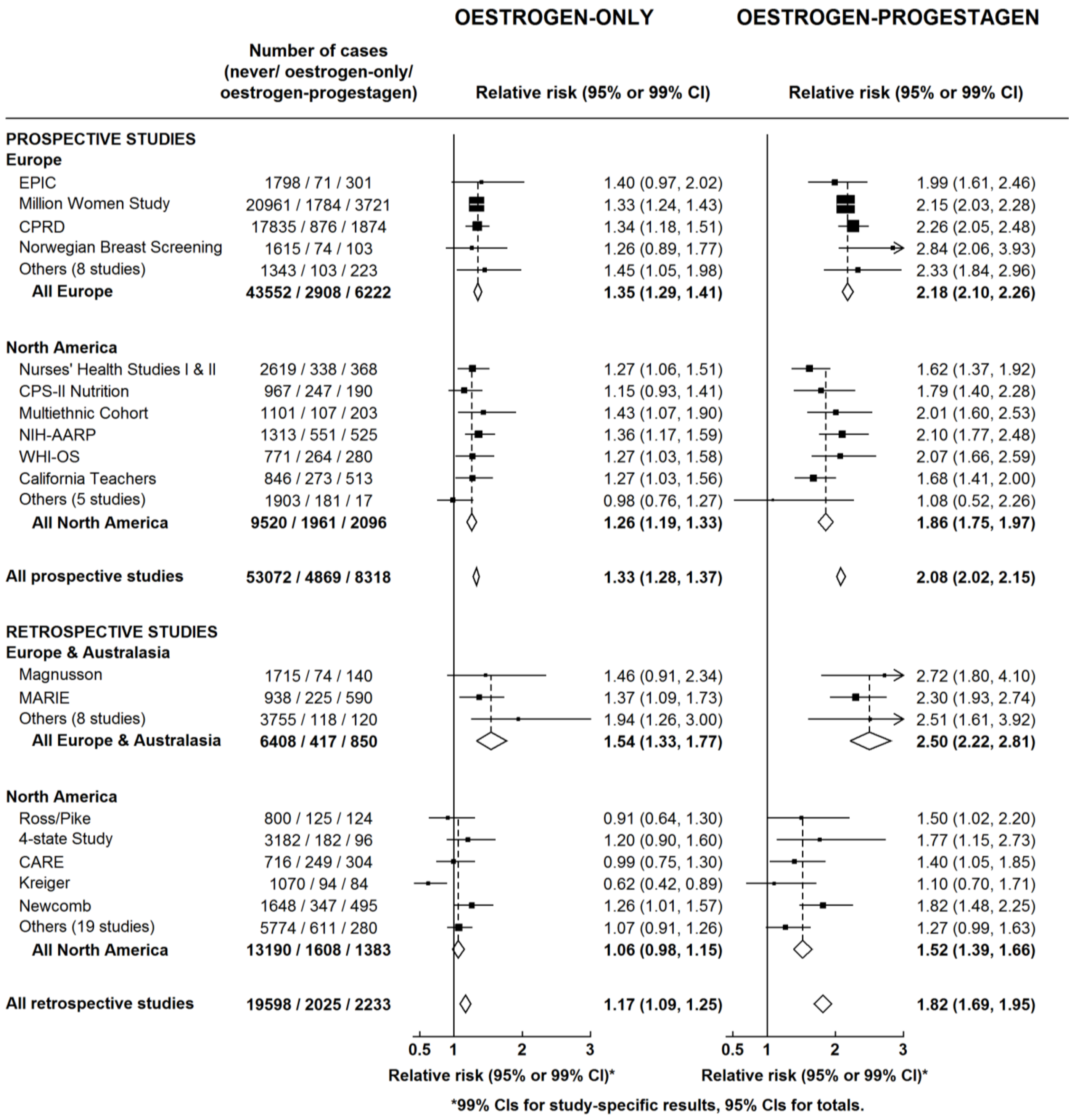

Heterogeneity, prospective vs retrospective studies: p<0.001 for each type of MHT

Figure S5: Relative risks in prospective studies for first use of MHT at age 40-44

Fully adjusted relative risks for current versus never-users of MHT

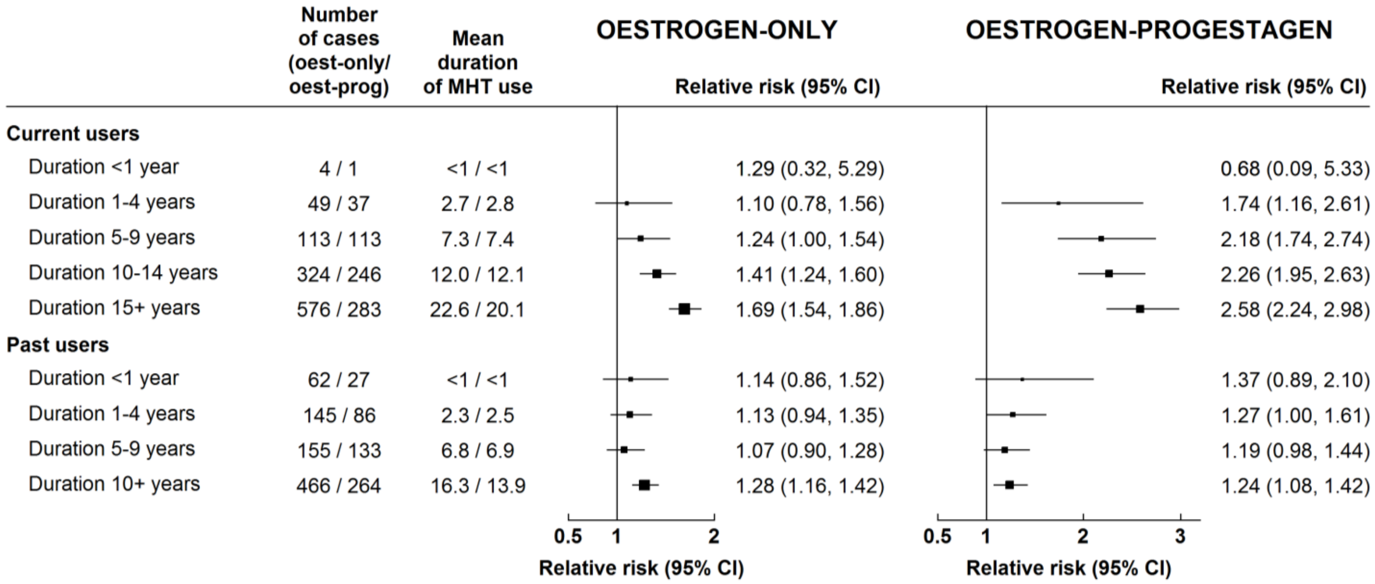

Figure S6: Relative risks in prospective studies for first use of MHT at age 45-49

Fully adjusted relative risks for current versus never-users of MHT

	Number of cases (oest-only/ oest-prog)	Mean duration of MHT use	OESTROGEN-ONLY Relative risk (95% CI)	OESTROGEN-PROGESTAGEN Relative risk (95% CI)
**Current users**				
Duration <1 year	13 / 10	<1 / <1	1.71 (0.86, 3.38)	0.88 (0.42, 1.83)
Duration 1-4 years	155 / 260	2.9 / 3.0	1.05 (0.86, 1.27)	1.52 (1.30, 1.77)
Duration 5-9 years	597 / 1123	7.2 / 7.1	1.36 (1.23, 1.49)	2.00 (1.86, 2.16)
Duration 10-14 years	682 / 894	11.7 / 11.6	1.40 (1.28, 1.52)	2.25 (2.07, 2.44)
Duration 15+ years	553 / 388	20.6 / 18.3	1.65 (1.49, 1.82)	2.82 (2.49, 3.20)
**Past users**				
Duration <1 year	103 / 134	<1 / <1	0.89 (0.71, 1.10)	0.95 (0.79, 1.15)
Duration 1-4 years	359 / 506	2.3 / 2.5	1.05 (0.94, 1.18)	1.09 (0.99, 1.20)
Duration 5-9 years	434 / 676	6.8 / 6.8	1.10 (0.99, 1.23)	1.15 (1.06, 1.25)
Duration 10+ years	692 / 725	14.3 / 12.6	1.26 (1.16, 1.37)	1.38 (1.27, 1.50)

Figure S7: Relative risks in prospective studies for first use of MHT at age 50-54

Fully adjusted relative risks for current versus never-users of MHT

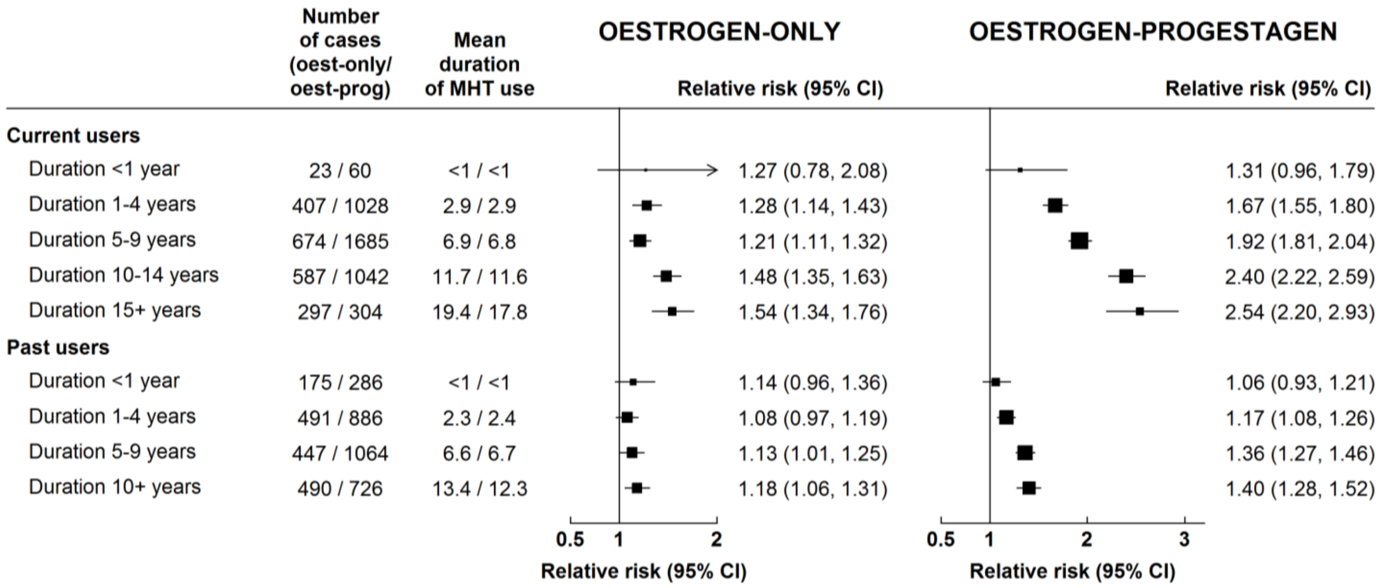

	Number of cases (oest-only/ oest-prog)	Mean duration of MHT use	OESTROGEN-ONLY Relative risk (95% CI)	OESTROGEN-PROGESTAGEN Relative risk (95% CI)
**Current users**				
Duration <1 year	23 / 60	<1 / <1	1.27 (0.78, 2.08)	1.31 (0.96, 1.79)
Duration 1-4 years	407 / 1028	2.9 / 2.9	1.28 (1.14, 1.43)	1.67 (1.55, 1.80)
Duration 5-9 years	674 / 1685	6.9 / 6.8	1.21 (1.11, 1.32)	1.92 (1.81, 2.04)
Duration 10-14 years	587 / 1042	11.7 / 11.6	1.48 (1.35, 1.63)	2.40 (2.22, 2.59)
Duration 15+ years	297 / 304	19.4 / 17.8	1.54 (1.34, 1.76)	2.54 (2.20, 2.93)
**Past users**				
Duration <1 year	175 / 286	<1 / <1	1.14 (0.96, 1.36)	1.06 (0.93, 1.21)
Duration 1-4 years	491 / 886	2.3 / 2.4	1.08 (0.97, 1.19)	1.17 (1.08, 1.26)
Duration 5-9 years	447 / 1064	6.6 / 6.7	1.13 (1.01, 1.25)	1.36 (1.27, 1.46)
Duration 10+ years	490 / 726	13.4 / 12.3	1.18 (1.06, 1.31)	1.40 (1.28, 1.52)

Figure S8: Relative risks in prospective studies for first use of MHT at age 55-59

Fully adjusted relative risks for current versus never-users of MHT

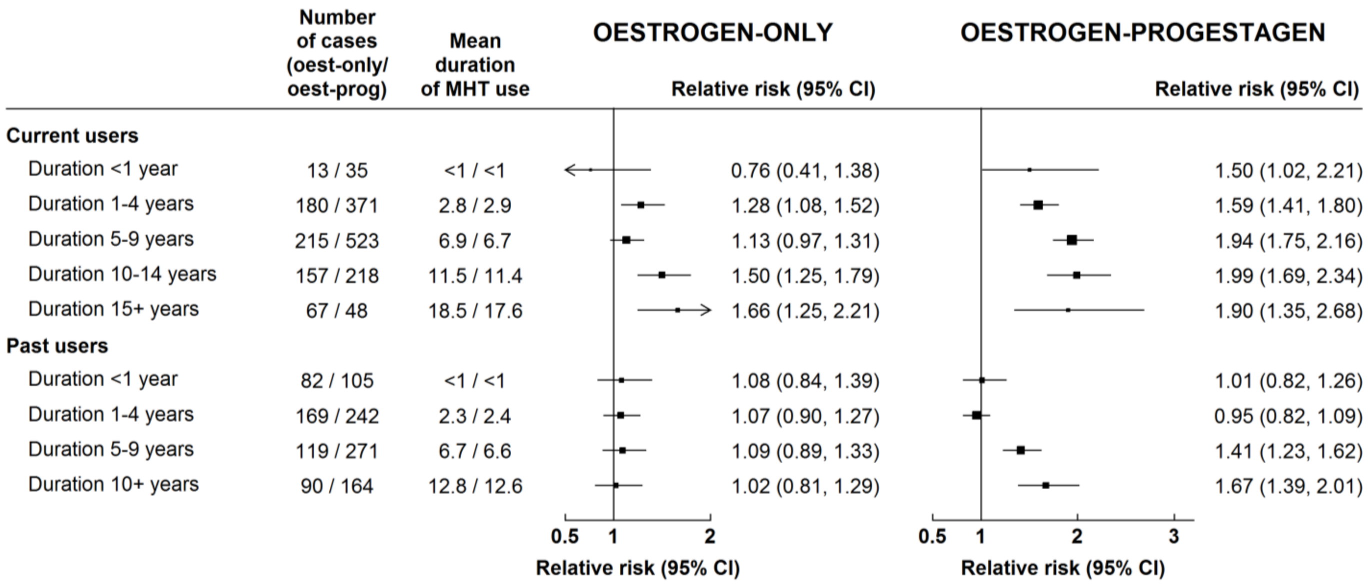

Figure S9: Relative risks in prospective studies for first use of MHT at age 60-69

Fully adjusted relative risks for current versus never-users of MHT

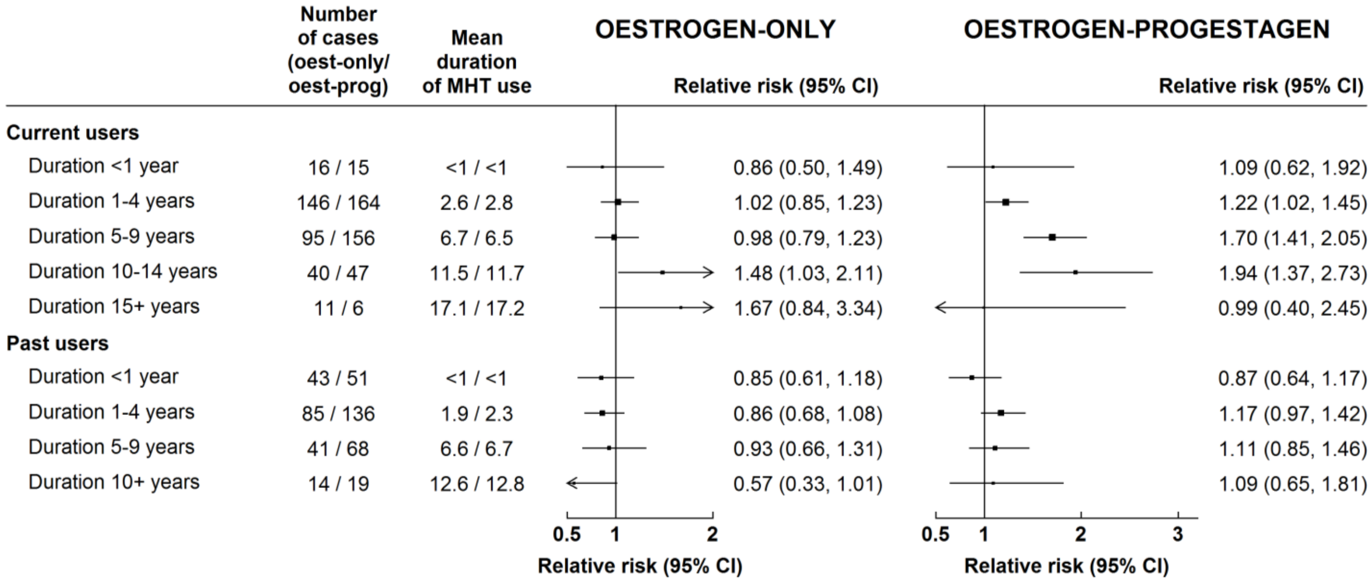

Figure S10: Relative risks in prospective studies by age at first use of MHT

Fully adjusted relative risks for current versus never-users of MHT

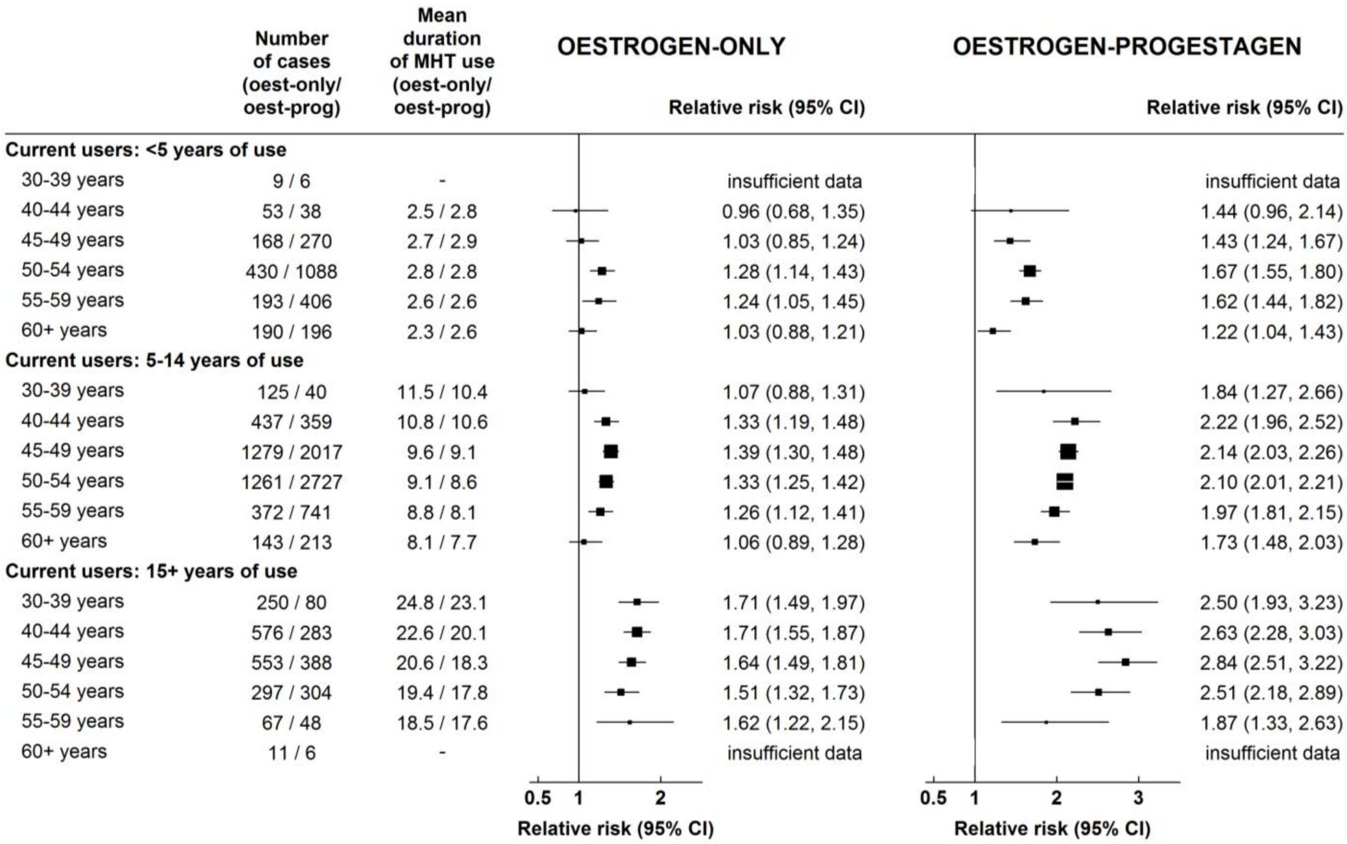

Figure S11: Relative risks in prospective studies for women who first used MHT at age 45-54

Fully adjusted relative risks for current versus never-users of MHT

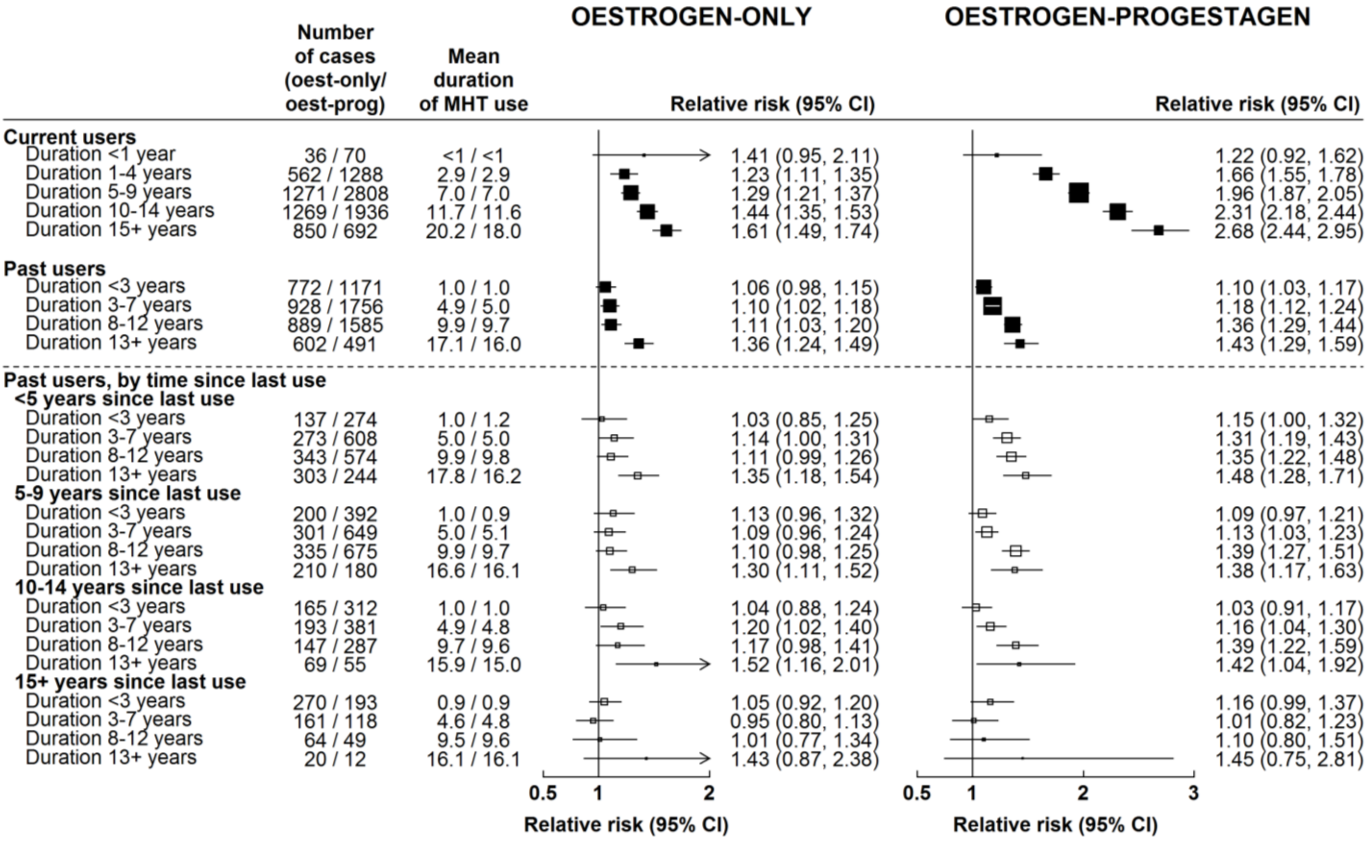

Figure S12: Relative risks in prospective studies by the time between menopause and first MHT use

Fully adjusted relative risks for current versus never-users of MHT

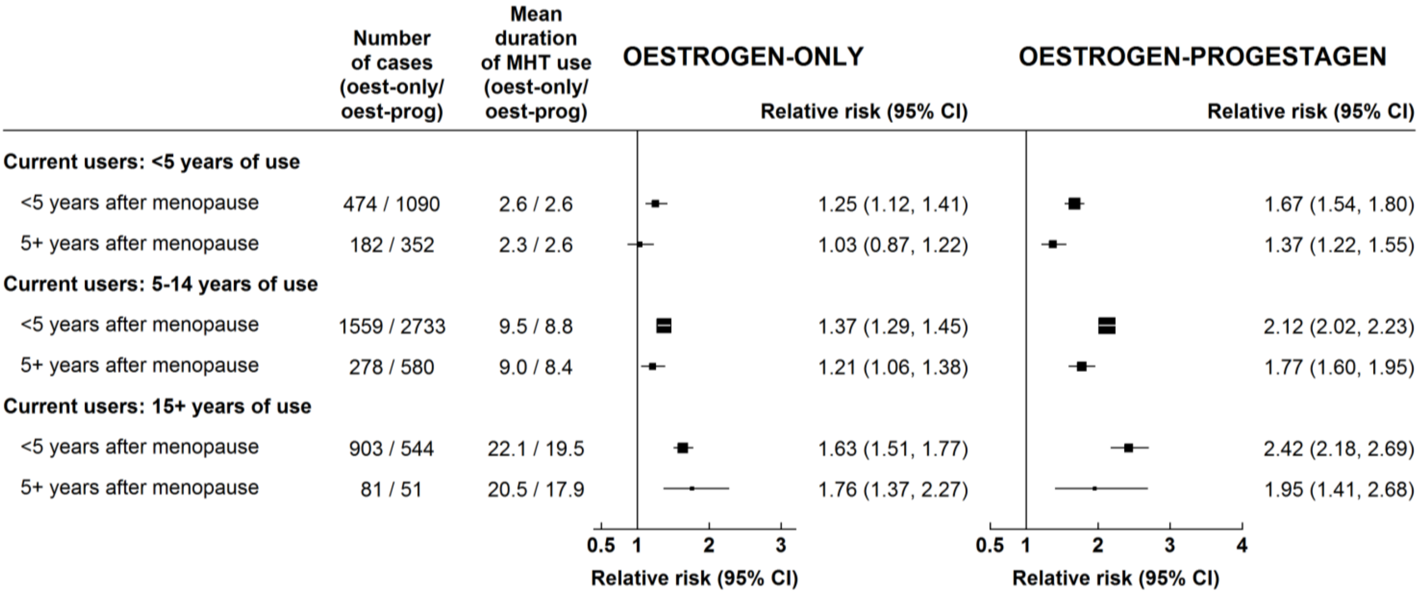

Figure S13: Sensitivity analyses in prospective studies: effect of restricting all women to those with <5 years between last report and index date

Fully adjusted relative risks for MHT users versus never-users

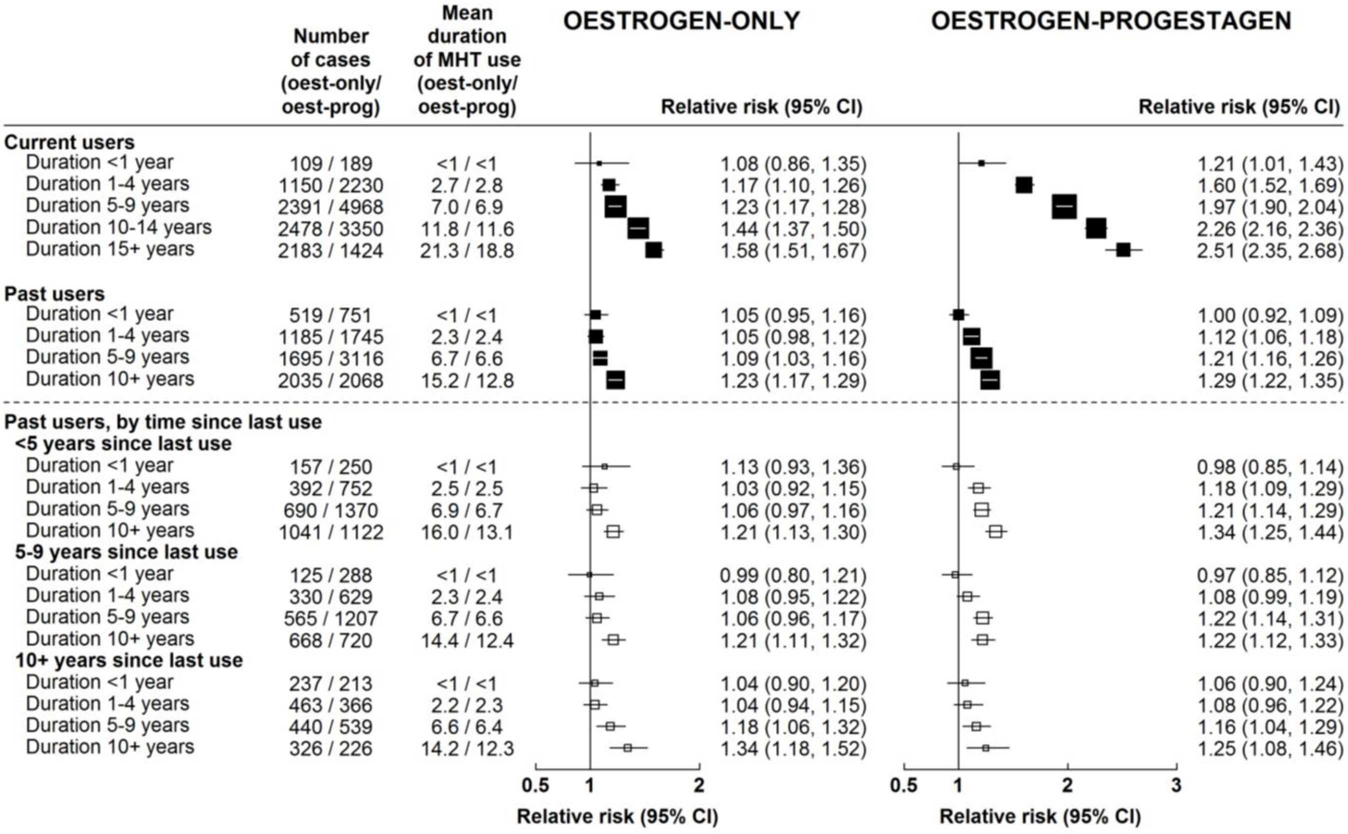

Figure S14: Sensitivity analyses in prospective studies: effect of various assumptions about continuation of MHT use in the period between last report and index date

Fully adjusted relative risks for current versus never-users of MHT

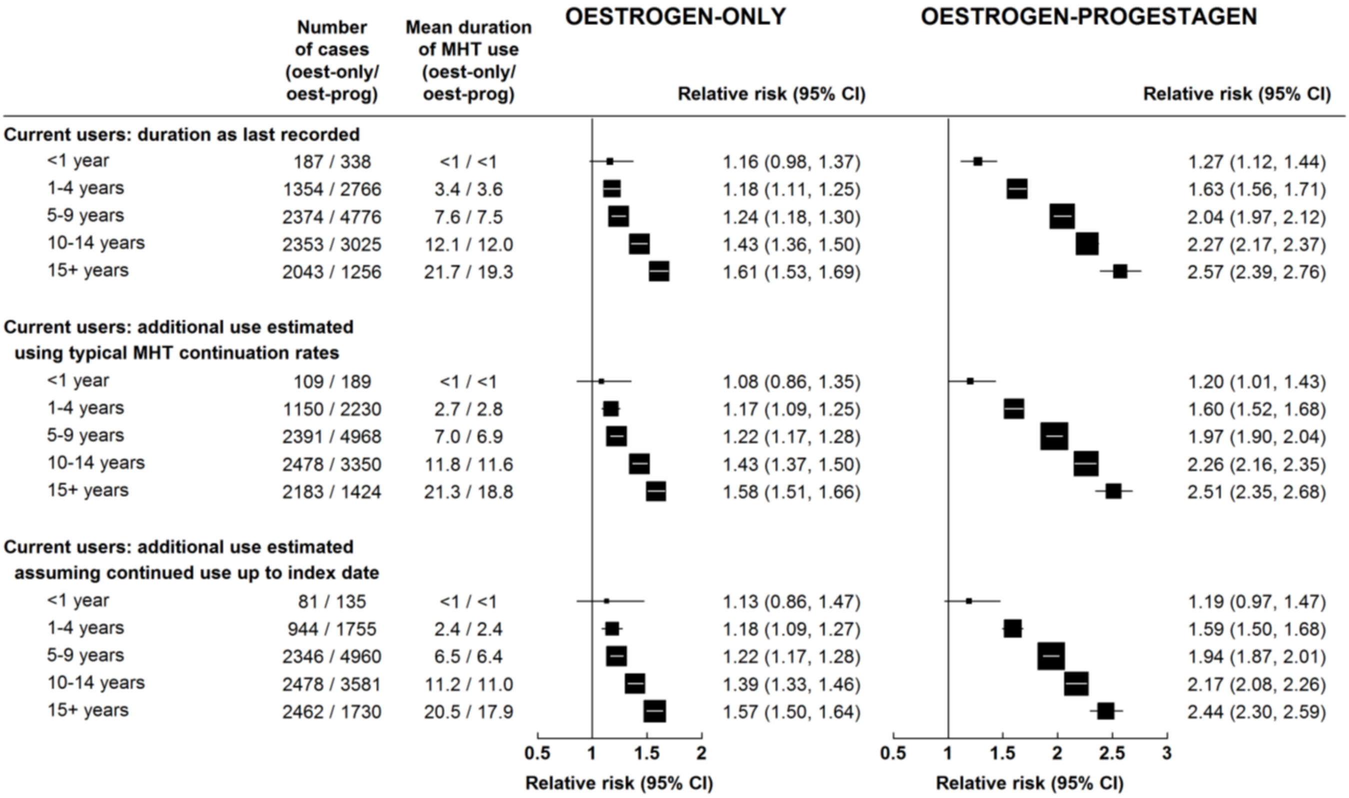

	Number of cases (oest-only/ oest-prog)	Mean duration of MHT use (oest-only/ oest-prog)	OESTROGEN-ONLY Relative risk (95% CI)	OESTROGEN-PROGESTAGEN Relative risk (95% CI)
**Current users: duration as last recorded**				
<1 year	187 / 338	<1 / <1	1.16 (0.98, 1.37)	1.27 (1.12, 1.44)
1-4 years	1354 / 2766	3.4 / 3.6	1.18 (1.11, 1.25)	1.63 (1.56, 1.71)
5-9 years	2374 / 4776	7.6 / 7.5	1.24 (1.18, 1.30)	2.04 (1.97, 2.12)
10-14 years	2353 / 3025	12.1 / 12.0	1.43 (1.36, 1.50)	2.27 (2.17, 2.37)
15+ years	2043 / 1256	21.7 / 19.3	1.61 (1.53, 1.69)	2.57 (2.39, 2.76)
**Current users: additional use estimated using typical MHT continuation rates**				
<1 year	109 / 189	<1 / <1	1.08 (0.86, 1.35)	1.20 (1.01, 1.43)
1-4 years	1150 / 2230	2.7 / 2.8	1.17 (1.09, 1.25)	1.60 (1.52, 1.68)
5-9 years	2391 / 4968	7.0 / 6.9	1.22 (1.17, 1.28)	1.97 (1.90, 2.04)
10-14 years	2478 / 3350	11.8 / 11.6	1.43 (1.37, 1.50)	2.26 (2.16, 2.35)
15+ years	2183 / 1424	21.3 / 18.8	1.58 (1.51, 1.66)	2.51 (2.35, 2.68)
**Current users: additional use estimated assuming continued use up to index date**				
<1 year	81 / 135	<1 / <1	1.13 (0.86, 1.47)	1.19 (0.97, 1.47)
1-4 years	944 / 1755	2.4 / 2.4	1.18 (1.09, 1.27)	1.59 (1.50, 1.68)
5-9 years	2346 / 4960	6.5 / 6.4	1.22 (1.17, 1.28)	1.94 (1.87, 2.01)
10-14 years	2478 / 3581	11.2 / 11.0	1.39 (1.33, 1.46)	2.17 (2.08, 2.26)
15+ years	2462 / 1730	20.5 / 17.9	1.57 (1.50, 1.64)	2.44 (2.30, 2.59)

Figure S15: Relative risks in prospective studies by specific types of oestrogenic and progestagenic constituents, including rare types

Fully adjusted relative risks for current versus never-users of MHT.

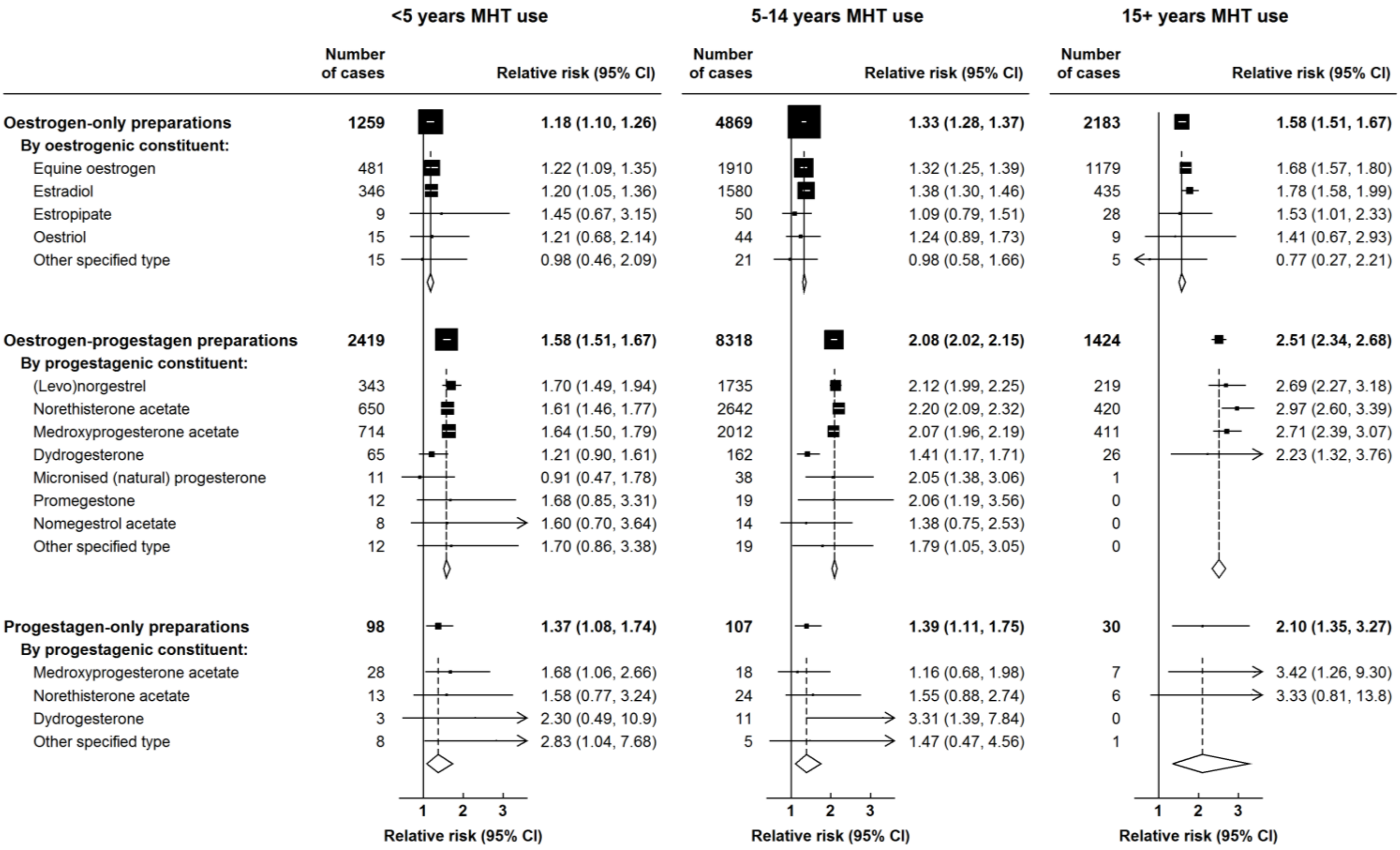

	<5 years MHT use: Number of cases	<5 years MHT use: Relative risk (95% CI)	5-14 years MHT use: Number of cases	5-14 years MHT use: Relative risk (95% CI)	15+ years MHT use: Number of cases	15+ years MHT use: Relative risk (95% CI)
**Oestrogen-only preparations**	**1259**	**1.18 (1.10, 1.26)**	**4869**	**1.33 (1.28, 1.37)**	**2183**	**1.58 (1.51, 1.67)**
**By oestrogenic constituent:**						
Equine oestrogen	481	1.22 (1.09, 1.35)	1910	1.32 (1.25, 1.39)	1179	1.68 (1.57, 1.80)
Estradiol	346	1.20 (1.05, 1.36)	1580	1.38 (1.30, 1.46)	435	1.78 (1.58, 1.99)
Estropipate	9	1.45 (0.67, 3.15)	50	1.09 (0.79, 1.51)	28	1.53 (1.01, 2.33)
Oestriol	15	1.21 (0.68, 2.14)	44	1.24 (0.89, 1.73)	9	1.41 (0.67, 2.93)
Other specified type	15	0.98 (0.46, 2.09)	21	0.98 (0.58, 1.66)	5	0.77 (0.27, 2.21)
**Oestrogen-progestagen preparations**	**2419**	**1.58 (1.51, 1.67)**	**8318**	**2.08 (2.02, 2.15)**	**1424**	**2.51 (2.34, 2.68)**
**By progestagenic constituent:**						
(Levo)norgestrel	343	1.70 (1.49, 1.94)	1735	2.12 (1.99, 2.25)	219	2.69 (2.27, 3.18)
Norethisterone acetate	650	1.61 (1.46, 1.77)	2642	2.20 (2.09, 2.32)	420	2.97 (2.60, 3.39)
Medroxyprogesterone acetate	714	1.64 (1.50, 1.79)	2012	2.07 (1.96, 2.19)	411	2.71 (2.39, 3.07)
Dydrogesterone	65	1.21 (0.90, 1.61)	162	1.41 (1.17, 1.71)	26	2.23 (1.32, 3.76)
Micronised (natural) progesterone	11	0.91 (0.47, 1.78)	38	2.05 (1.38, 3.06)	1	
Promegestone	12	1.68 (0.85, 3.31)	19	2.06 (1.19, 3.56)	0	
Nomegestrol acetate	8	1.60 (0.70, 3.64)	14	1.38 (0.75, 2.53)	0	
Other specified type	12	1.70 (0.86, 3.38)	19	1.79 (1.05, 3.05)	0	
**Progestagen-only preparations**	**98**	**1.37 (1.08, 1.74)**	**107**	**1.39 (1.11, 1.75)**	**30**	**2.10 (1.35, 3.27)**
**By progestagenic constituent:**						
Medroxyprogesterone acetate	28	1.68 (1.06, 2.66)	18	1.16 (0.68, 1.98)	7	3.42 (1.26, 9.30)
Norethisterone acetate	13	1.58 (0.77, 3.24)	24	1.55 (0.88, 2.74)	6	3.33 (0.81, 13.8)
Dydrogesterone	3	2.30 (0.49, 10.9)	11	3.31 (1.39, 7.84)	0	
Other specified type	8	2.83 (1.04, 7.68)	5	1.47 (0.47, 4.56)	1	

Figure S16: Relative risks in prospective studies for ER-positive breast cancer

Fully adjusted relative risks for MHT users versus never-users

	Number of cases (oest-only/ oest-prog)	Mean duration of MHT use (oest-only/ oest-prog)	OESTROGEN-ONLY Relative risk (95% CI)	OESTROGEN-PROGESTAGEN Relative risk (95% CI)
**Current users**				
Duration <5 years	582 / 1228	2.4 / 2.5	1.27 (1.16, 1.39)	1.84 (1.72, 1.96)
Duration 5-9 years	1227 / 2688	7.0 / 6.9	1.35 (1.27, 1.44)	2.30 (2.19, 2.41)
Duration 10+ years	2285 / 2631	16.9 / 13.9	1.61 (1.54, 1.69)	2.70 (2.58, 2.84)
**Past users**				
Duration <5 year	1084 / 1749	1.6 / 1.7	1.08 (1.01, 1.16)	1.18 (1.12, 1.24)
Duration 5+ years	2601 / 3742	11.5 / 9.1	1.21 (1.15, 1.26)	1.32 (1.27, 1.37)
**Past users, by time since last use**				
**<5 years since last use**				
Duration <5 years	291 / 536	1.8 / 1.9	1.09 (0.96, 1.25)	1.18 (1.07, 1.30)
Duration 5+ years	1011 / 1487	12.8 / 9.7	1.12 (1.04, 1.20)	1.29 (1.21, 1.37)
**5-9 years since last use**				
Duration <5 years	267 / 598	1.7 / 1.7	1.05 (0.92, 1.21)	1.10 (1.01, 1.21)
Duration 5+ years	906 / 1414	11.3 / 9.1	1.22 (1.13, 1.31)	1.32 (1.25, 1.41)
**10+ years since last use**				
Duration <5 years	526 / 615	1.4 / 1.5	1.09 (0.99, 1.20)	1.25 (1.14, 1.37)
Duration 5+ years	684 / 841	10.0 / 8.2	1.32 (1.21, 1.44)	1.36 (1.26, 1.48)

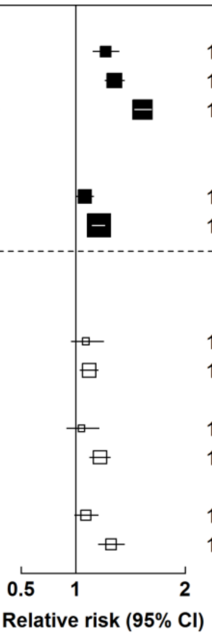

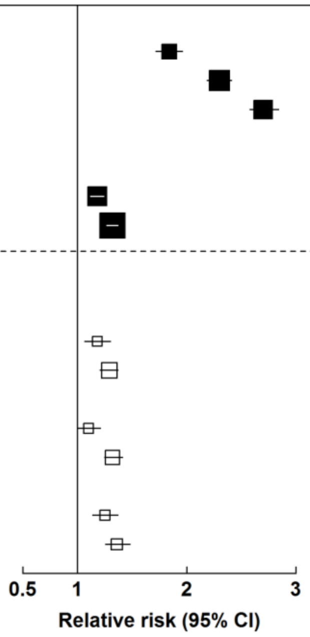

Figure S17: Relative risks in prospective studies for ER-negative breast cancer

Fully adjusted relative risks for users versus never-users of MHT

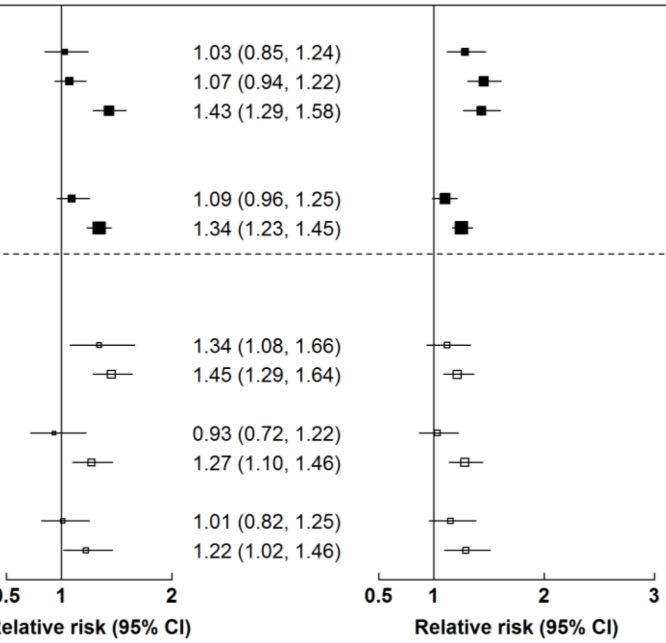

	Number of cases (oest-only/ oest-prog)	Mean duration of MHT use (oest-only/ oest-prog)	OESTROGEN-ONLY Relative risk (95% CI)	OESTROGEN-PROGESTAGEN Relative risk (95% CI)
**Current users**				
Duration <5 years	130 / 249	2.5 / 2.5	1.03 (0.85, 1.24)	1.28 (1.12, 1.47)
Duration 5-9 years	266 / 455	6.9 / 7.0	1.07 (0.94, 1.22)	1.45 (1.31, 1.61)
Duration 10+ years	443 / 353	16.0 / 13.7	1.43 (1.29, 1.58)	1.43 (1.27, 1.60)
**Past users**				
Duration <5 year	257 / 458	1.6 / 1.6	1.09 (0.96, 1.25)	1.10 (0.99, 1.21)
Duration 5+ years	677 / 940	10.9 / 8.7	1.34 (1.23, 1.45)	1.25 (1.17, 1.35)
**Past users, by time since last use**				
**<5 years since last use**				
Duration <5 years	100 / 158	1.9 / 1.9	1.34 (1.08, 1.66)	1.12 (0.94, 1.33)
Duration 5+ years	322 / 396	11.8 / 9.2	1.45 (1.29, 1.64)	1.21 (1.09, 1.36)
**5-9 years since last use**				
Duration <5 years	60 / 165	1.7 / 1.4	0.93 (0.72, 1.22)	1.03 (0.87, 1.22)
Duration 5+ years	218 / 355	10.5 / 8.3	1.27 (1.10, 1.46)	1.28 (1.14, 1.44)
**10+ years since last use**				
Duration <5 years	97 / 135	1.2 / 1.4	1.01 (0.82, 1.25)	1.15 (0.96, 1.38)
Duration 5+ years	137 / 189	9.2 / 8.6	1.22 (1.02, 1.46)	1.29 (1.10, 1.51)

Figure S18: Relevance of body mass index to ER+ and ER– breast cancer incidence at ages 55-64 years in never-users, and in current users during years 5-14 of MHT use

Adjusted relative risks from prospective studies for ER+ and for ER– breast cancer, taking never-users with BMI 25-29 kg/m^2 as the reference group, were multiplied by estimates of the incidence of ER+ and of ER– breast cancer in never-users aged 55-64 of average weight in Western countries (2.4% and 0.6% respectively; figure 6). Note that figure 6 includes the findings for all breast cancer, including cancers of unknown ER status.

Results for never-users are shown as solid lines; for oestrogen-only MHT as dotted lines; and for oestrogen-progestagen MHT as dashed lines.

Results for ER+ disease are shown in black and for ER– disease in grey.

BMI groups: <25 (lean), 25-30 (overweight), ≥30 (obese) kg/m^2, plotted against group means.

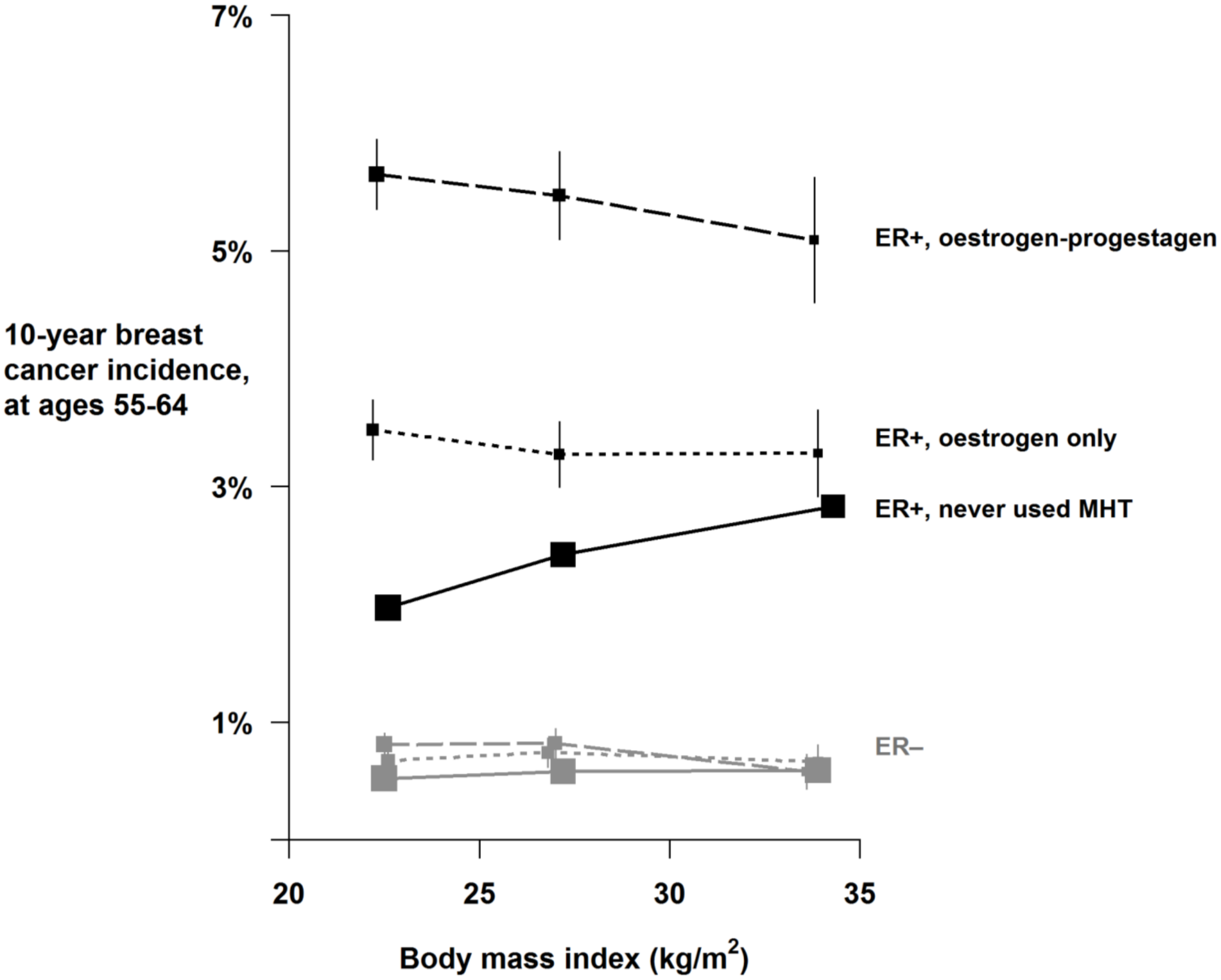

Figure S19: Relative risks in prospective studies for various subgroups of women

Fully adjusted relative risks for current versus never-users of MHT. Results in each row are calculated independently of those in any other row.

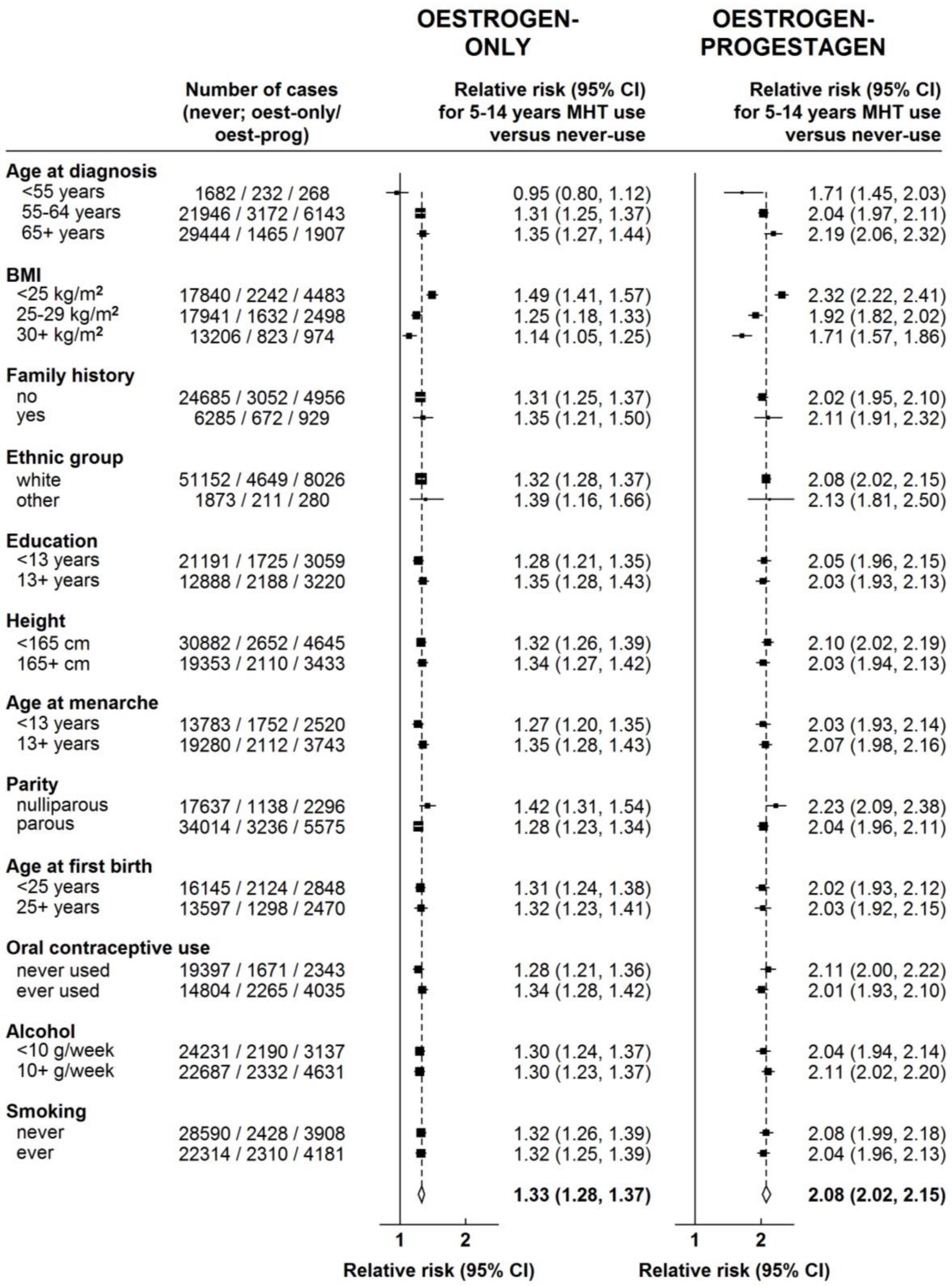

Figure S20: Relative risks in RETROSPECTIVE studies, ever versus never use of MHT

Fully adjusted relative risks for ever users versus never-users of MHT

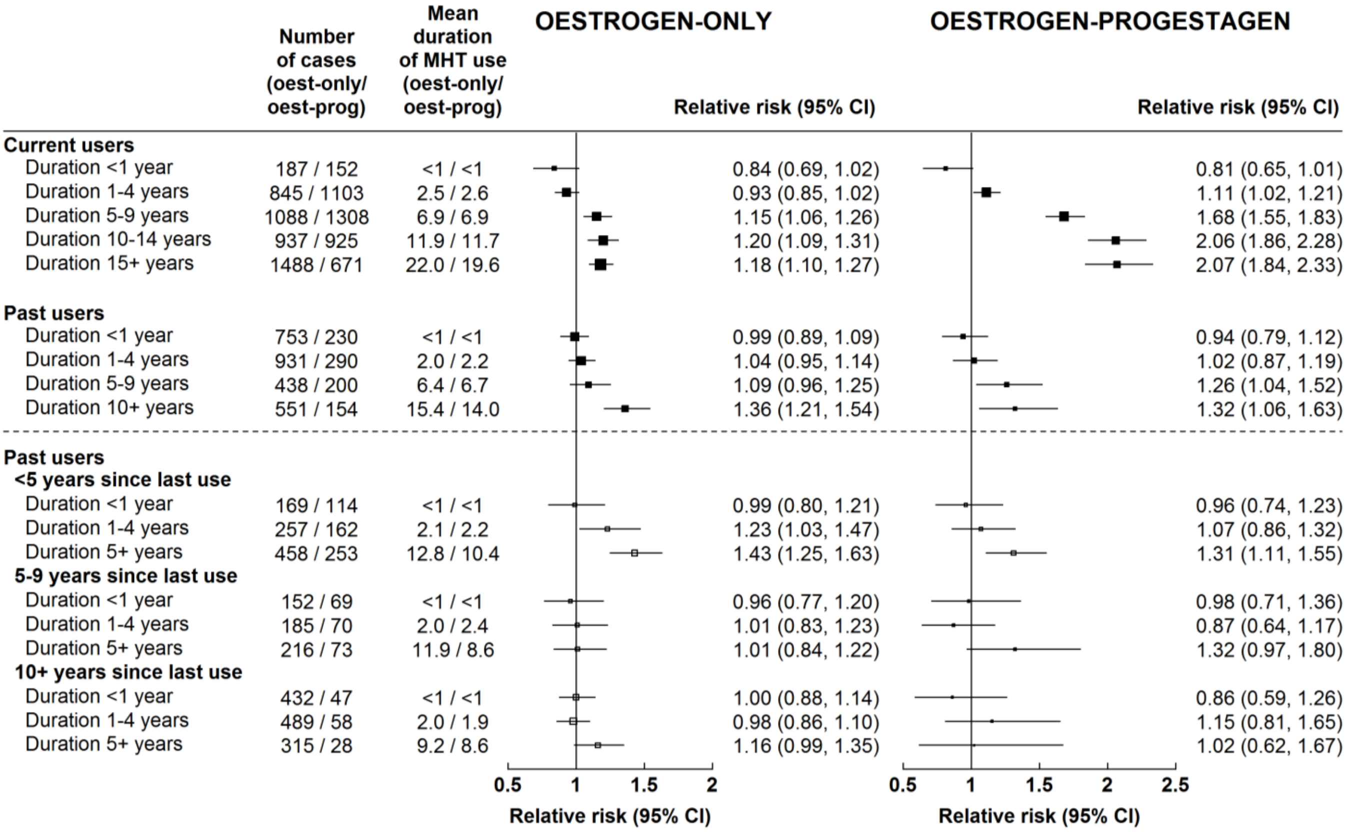

	Number of cases (oest-only/ oest-prog)	Mean duration of MHT use (oest-only/ oest-prog)	OESTROGEN-ONLY Relative risk (95% CI)	OESTROGEN-PROGESTAGEN Relative risk (95% CI)
**Current users**				
Duration <1 year	187 / 152	<1 / <1	0.84 (0.69, 1.02)	0.81 (0.65, 1.01)
Duration 1-4 years	845 / 1103	2.5 / 2.6	0.93 (0.85, 1.02)	1.11 (1.02, 1.21)
Duration 5-9 years	1088 / 1308	6.9 / 6.9	1.15 (1.06, 1.26)	1.68 (1.55, 1.83)
Duration 10-14 years	937 / 925	11.9 / 11.7	1.20 (1.09, 1.31)	2.06 (1.86, 2.28)
Duration 15+ years	1488 / 671	22.0 / 19.6	1.18 (1.10, 1.27)	2.07 (1.84, 2.33)
**Past users**				
Duration <1 year	753 / 230	<1 / <1	0.99 (0.89, 1.09)	0.94 (0.79, 1.12)
Duration 1-4 years	931 / 290	2.0 / 2.2	1.04 (0.95, 1.14)	1.02 (0.87, 1.19)
Duration 5-9 years	438 / 200	6.4 / 6.7	1.09 (0.96, 1.25)	1.26 (1.04, 1.52)
Duration 10+ years	551 / 154	15.4 / 14.0	1.36 (1.21, 1.54)	1.32 (1.06, 1.63)
**Past users**				
**<5 years since last use**				
Duration <1 year	169 / 114	<1 / <1	0.99 (0.80, 1.21)	0.96 (0.74, 1.23)
Duration 1-4 years	257 / 162	2.1 / 2.2	1.23 (1.03, 1.47)	1.07 (0.86, 1.32)
Duration 5+ years	458 / 253	12.8 / 10.4	1.43 (1.25, 1.63)	1.31 (1.11, 1.55)
**5-9 years since last use**				
Duration <1 year	152 / 69	<1 / <1	0.96 (0.77, 1.20)	0.98 (0.71, 1.36)
Duration 1-4 years	185 / 70	2.0 / 2.4	1.01 (0.83, 1.23)	0.87 (0.64, 1.17)
Duration 5+ years	216 / 73	11.9 / 8.6	1.01 (0.84, 1.22)	1.32 (0.97, 1.80)
**10+ years since last use**				
Duration <1 year	432 / 47	<1 / <1	1.00 (0.88, 1.14)	0.86 (0.59, 1.26)
Duration 1-4 years	489 / 58	2.0 / 1.9	0.98 (0.86, 1.10)	1.15 (0.81, 1.65)
Duration 5+ years	315 / 28	9.2 / 8.6	1.16 (0.99, 1.35)	1.02 (0.62, 1.67)

Figure S21: Relative risks in RETROSPECTIVE studies, by age at first use of MHT

Fully adjusted relative risks for current versus never-users of MHT

	Number of cases (oest-only/ oest-prog)	Mean duration of MHT use (oest-only/ oest-prog)	OESTROGEN-ONLY Relative risk (95% CI)	OESTROGEN-PROGESTAGEN Relative risk (95% CI)
**Current users: <5 years of use**				
30-39 years	33 / 5	-	insufficient data	insufficient data
40-44 years	61 / 30	2.4 / 2.6	0.90 (0.63, 1.29)	1.10 (0.66, 1.82)
45-49 years	216 / 247	2.4 / 2.4	1.28 (1.05, 1.55)	1.48 (1.21, 1.80)
50-54 years	346 / 561	2.2 / 2.3	1.06 (0.91, 1.23)	1.16 (1.03, 1.32)
55-59 years	184 / 269	1.8 / 2.2	0.81 (0.66, 0.98)	0.89 (0.75, 1.05)
60+ years	170 / 136	1.5 / 1.7	0.56 (0.46, 0.68)	0.71 (0.57, 0.89)
**Current users: 5-14 years of use**				
30-39 years	120 / 18	9.7 / 10.6	1.45 (1.12, 1.88)	1.47 (0.79, 2.75)
40-44 years	257 / 160	10.3 / 10.1	1.15 (0.97, 1.36)	1.87 (1.48, 2.36)
45-49 years	657 / 669	9.5 / 9.3	1.24 (1.11, 1.38)	1.91 (1.70, 2.15)
50-54 years	617 / 966	8.8 / 8.6	1.16 (1.03, 1.30)	1.99 (1.80, 2.20)
55-59 years	214 / 315	8.6 / 8.4	0.98 (0.81, 1.18)	1.43 (1.21, 1.69)
60+ years	94 / 69	7.7 / 7.3	0.96 (0.73, 1.28)	1.41 (0.98, 2.02)
**Current users: 15+ years of use**				
30-39 years	363 / 79	25.3 / 24.0	1.13 (0.99, 1.30)	2.40 (1.74, 3.32)
40-44 years	414 / 173	22.0 / 20.5	1.18 (1.03, 1.35)	2.12 (1.69, 2.64)
45-49 years	402 / 221	19.8 / 18.4	1.16 (1.01, 1.34)	2.25 (1.83, 2.77)
50-54 years	177 / 156	18.6 / 17.3	1.14 (0.92, 1.42)	1.89 (1.47, 2.43)
55-59 years	23 / 13	18.5 / 16.6	0.85 (0.48, 1.50)	1.15 (0.53, 2.49)
60+ years	3 / 3	-	insufficient data	insufficient data

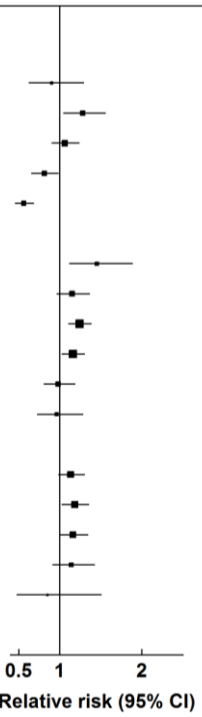

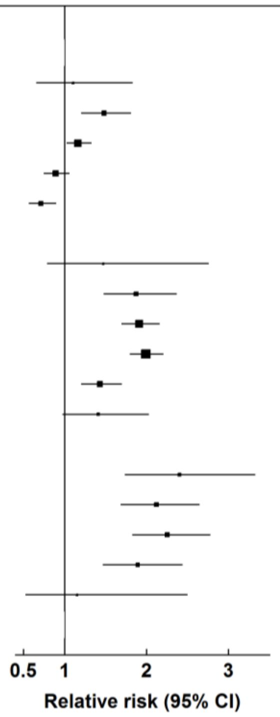

Figure S22: Relative risks in <u>RETROSPECTIVE</u> studies by the time between menopause and first use of MHT

Fully adjusted relative risks for current versus never-users of MHT

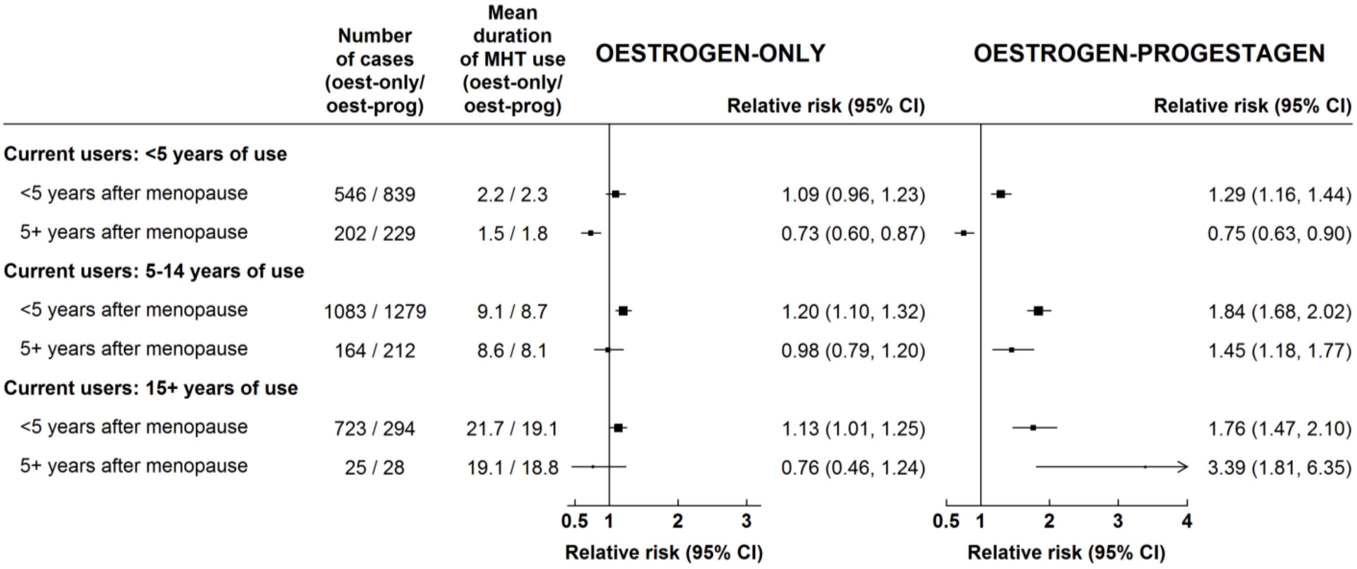

Figure S23: Relative risks in RETROSPECTIVE studies for different MHT preparations

Fully adjusted relative risks for current versus never-users of MHT

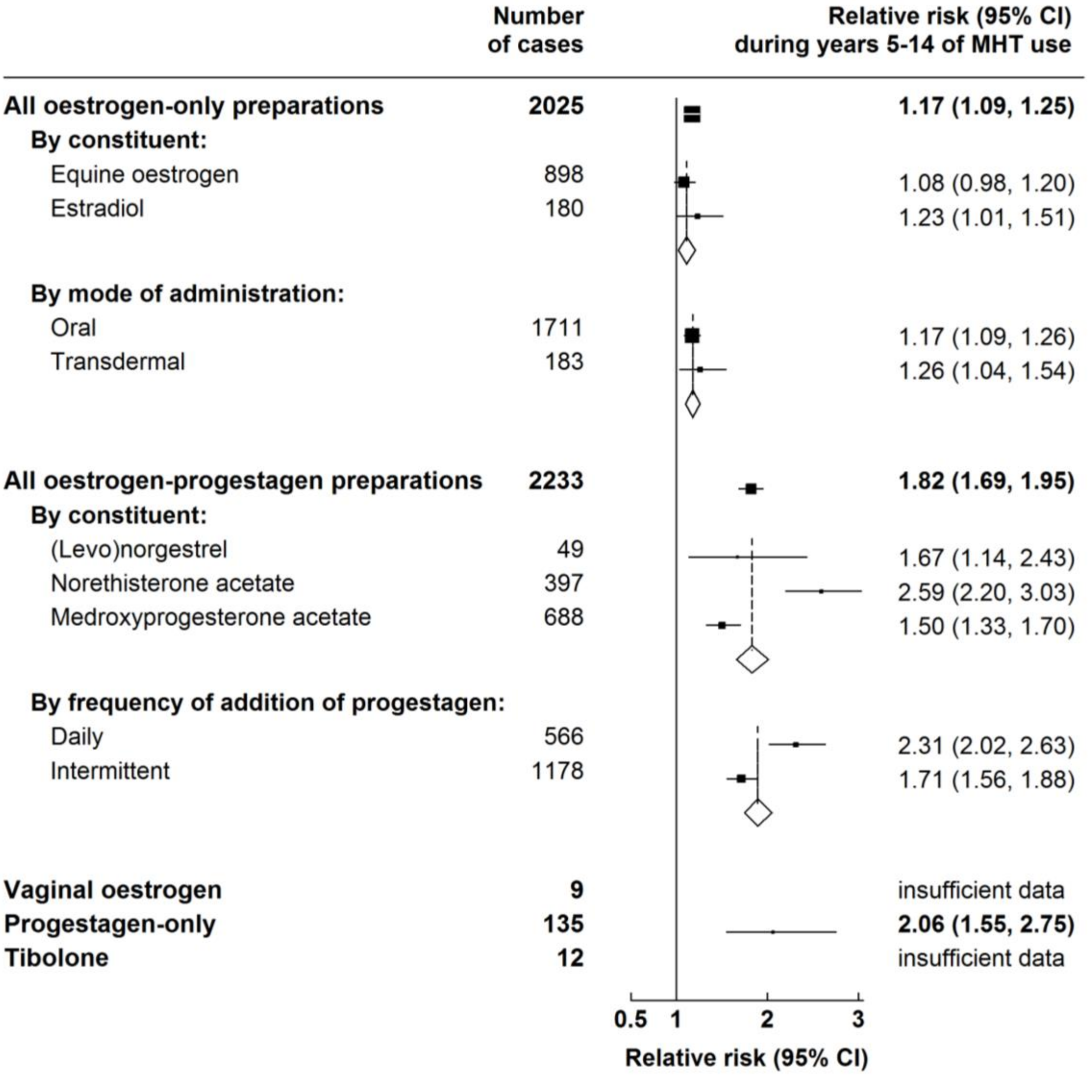

Figure S24: Relative risks in RETROSPECTIVE studies for various tumour characteristics

Fully adjusted relative risks for users versus never-users of MHT

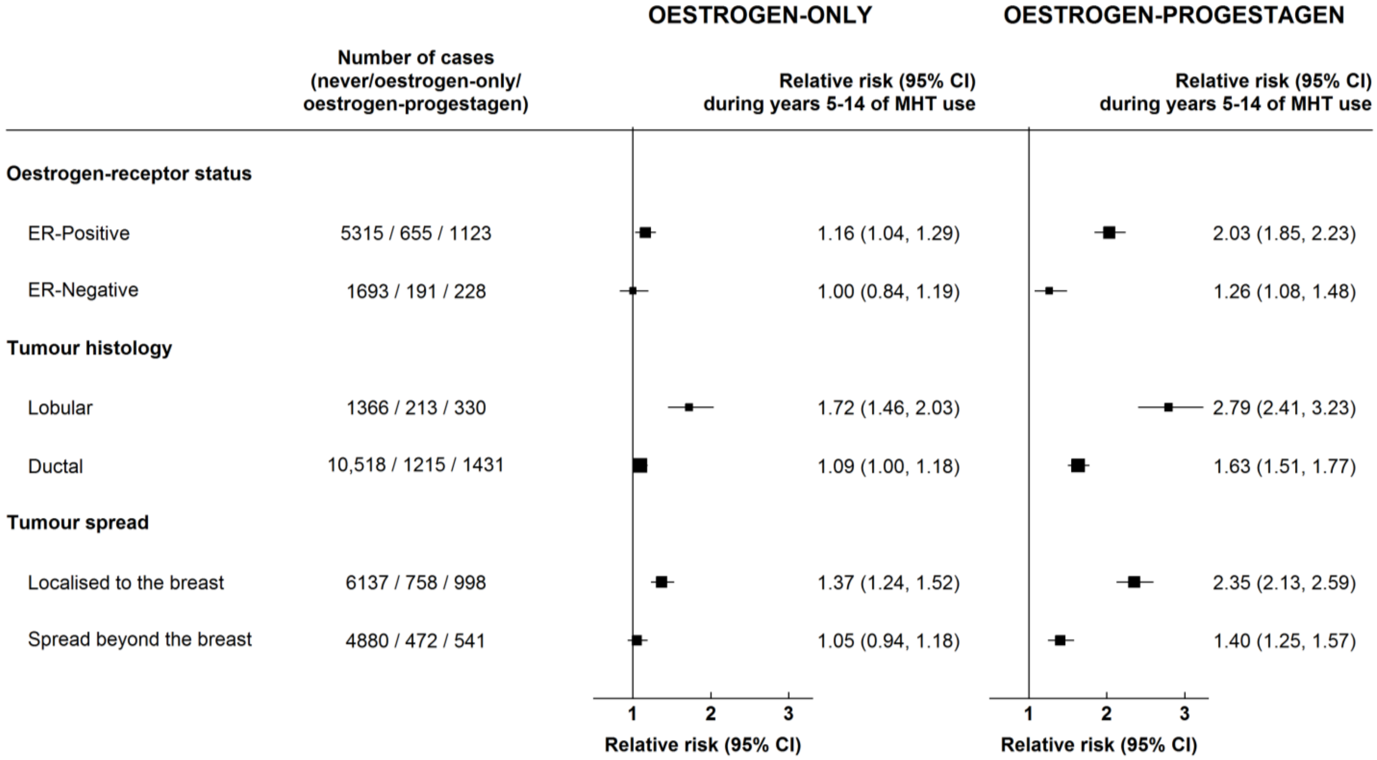

Figure S25: Relative risks in <u>RETROSPECTIVE</u> studies for various subgroups of women

Fully adjusted relative risks for current versus never-users of MHT. Results in each row are calculated independently of those in any other row.

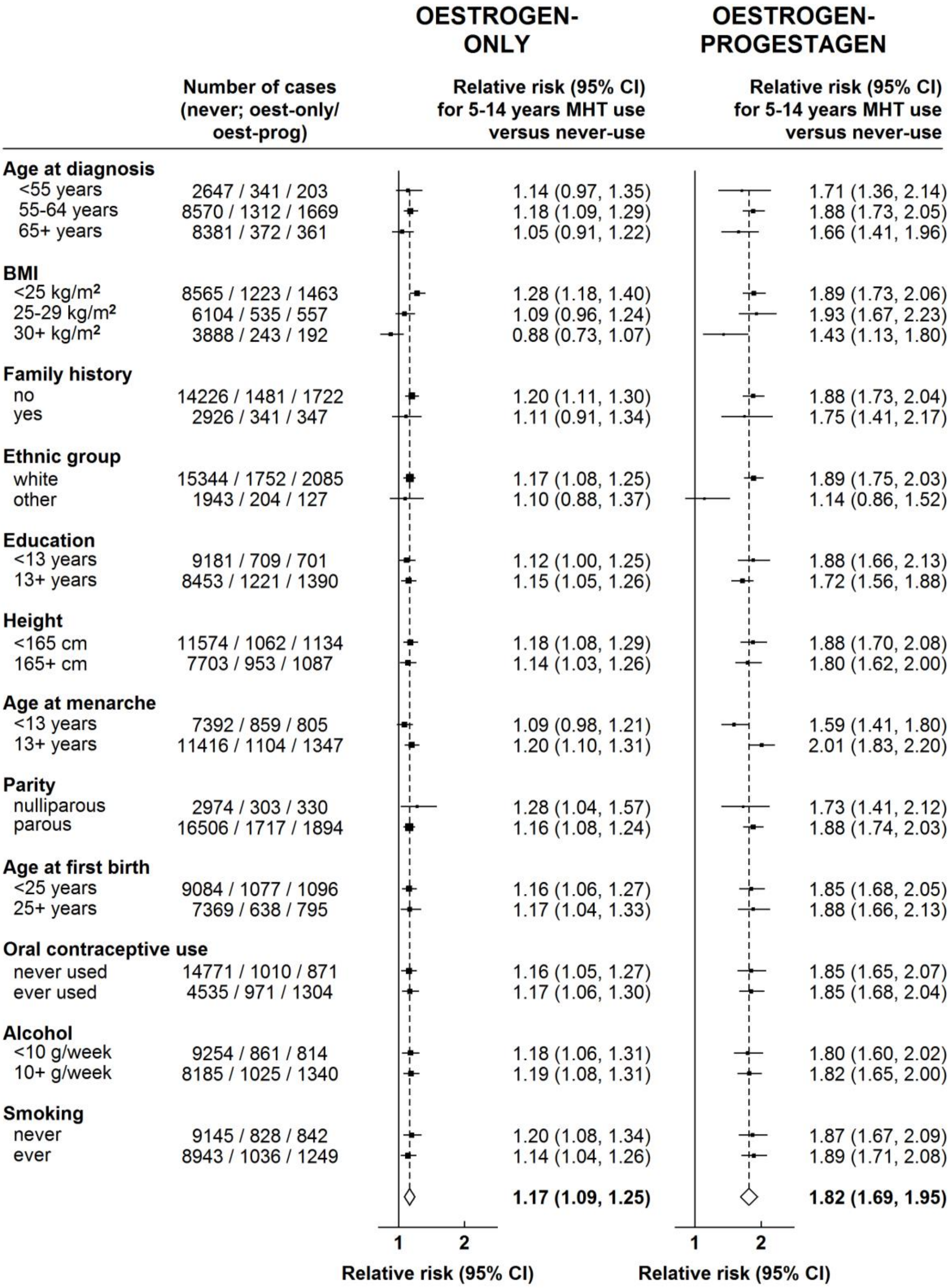

Menopausal hormone therapy and 20-year breast cancer mortality

(Research Letter, published online in the Lancet on 29 August 2019; doi.org/10.1016/ S0140-6736(19)31709-X)

The Collaborative Group on Hormonal Factors in Breast Cancer has brought together the worldwide evidence on menopausal hormone therapy (MHT) and the incidence of invasive breast cancer.[1] All types of MHT examined, except vaginal oestrogens, were associated with a significant excess incidence of breast cancer. Both among current users and among past users, the risks increased steadily with duration of MHT use. Risks were greater for oestrogen-progestagen than for oestrogen-only preparations, and some excess risk persisted for more than a decade after cessation of use. The collaboration collected no information, however, on breast cancer mortality. In this Research Letter we report the findings on MHT use at recruitment and 20-year breast cancer mortality from a large population-based prospective study, complementing the collaborative findings for incident breast cancer.

The Million Women Study (which also contributed information on incident breast cancer to the collaboration[1]) recruited 1·3 million women from 66 National Health Service breast screening centres in the UK in 1998 (range 1996–2001).[2] The present analyses are restricted to the 907 162 women who at recruitment were free from breast cancer and already postmenopausal. Among them, about a third were current users of MHT of known type, a sixth were past users (often of an unspecified type of MHT), and half were never-users. There was no material difference between these current, past, and never users in the age-standardised proportions who, about 3 years after recruitment, accepted their next mammographic breast screening invitation (90·9%, 90·5%, and 90·2%, respectively). Later follow-up showed that those who were current users at recruitment would on average continue MHT use for about a further 5 years, but that few of the past or never-users would use MHT after recruitment.

All study participants were followed up to Jan 1, 2018, about 20 years after recruitment, by electronic linkage to UK national death registers, which assign underlying causes and have negligible loss to follow-up. Cox regression (censored at the time of emigration from the UK, or death from another cause) related MHT use at recruitment to breast cancer mortality over the next 20 years, using adjustments similar to those in the collaborative analyses.[1] During follow-up, 7086/907 162 (0.8%) died from breast cancer (figure). Where the ER status of the tumour that caused death was known, three quarters had been ER positive.

Both for oestrogen-only and for oestrogen-progestagen preparations, women who had been current users at recruitment had significant excess breast cancer mortality risks ($p<0.0001$). These were greater the longer the duration of MHT use had been at recruitment. In each of the four categories of current user the eventual duration of MHT use would, on average, have been about 5 years longer than at recruitment: but, at a resurvey about 8 years after recruitment, there had been widespread cessation of use.[2] Women who at recruitment had been past users with <5 years prior use of MHT (mean: about 1 year) did not have a significant excess mortality from breast cancer (rate ratio 0.99, 95% CI 0·91-1.06). By contrast, past users with longer prior use of MHT (mean: about 8 years) did have a significant excess mortality from breast cancer over the next 20 years (rate ratio 1·24, 1·12-1·38, p=0·0005). These results for 20-year breast cancer mortality are consistent with the collaborative findings for the effects of current and past MHT use on breast cancer incidence.[1]

References

1. Collaborative Group on Hormonal Factors in Breast Cancer. Type and timing of menopausal hormone therapy and breast cancer risk: individual participant meta-analysis of the worldwide epidemiological evidence (ie, this report). Published online by the Lancet on 29 August 2019

2. Green J, Reeves G, Floud S et al. Cohort Profile: the Million Women Study. Int J Epi 2018: 1-7; doi: 10.1093/ije/dyy065

Figure S26: 20-year breast cancer mortality rate ratio in relation to MHT use at the time of recruitment into the Million Women Study

	Breast cancer deaths/ Population at the time of recruitment	Years of MHT use at recruitment* (mean and SD)	Breast cancer mortality rate ratio (95% CI)
**Never-user**	3523 / 476 902	0.0	1.00 (reference)
**Current user, oestrogen-only**			
<5 years use	231 / 31 996	2.4 (1.4)	1.15 (1.01, 1.32)
5+ years use	661 / 79 833	9.6 (4.3)	1.35 (1.24, 1.47)
**Current user, oestrogen-progestagen**			
<5 years use	557 / 65 188	2.3 (1.4)	1.39 (1.27, 1.53)
5+ years use	905 / 86 282	8.4 (3.2)	1.64 (1.52, 1.76)
**Past user**			
<5 years use	816 / 119 475	1.1 (1.3)	0.99 (0.91, 1.06)
5+ years use	393 / 47 516	8.0 (3.1)	1.24 (1.12, 1.38)

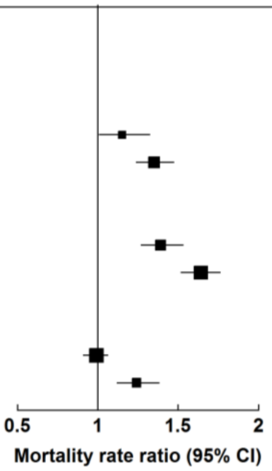

At recruitment (mean year: 1998), all were free from breast cancer and postmenopausal. Use would have continued for an average of about 5 years after recruitment in current users, but few who were past or never users at recruitment would have used MHT thereafter. Half the women were resurveyed about 8 years after recruitment, by which time there had been widespread cessation. The mean (SD) years of MHT use reported at resurvey by women in the above 7 categories of use at recruitment (which includes use before and use after recruitment) had become, respectively, 0.1 years for never-users, 7.0 (3.7) and 13.5 (5.5) years for those who had been using oestrogen-only MHT, 6.6 (3.4) and 11.8 (4.3) years for those who had been using oestrogen-progestagen MHT, and 1.2 (2.1) and 7.8 (4.4) years in those who had been past users. MHT=menopausal hormone therapy.
